# HEIWA: Hybrid Explicit–Implicit Water Attention Affects Force Field Accuracy in Structure-Based Protein Language Model ESM3

**DOI:** 10.1021/acs.jcim.6c01844

**Published:** 2026-10-23

**Authors:** Ngai Hei Ernest Ho, Chao Tung Yang, I-Son Ng, Jo-Shu Chang

**Affiliations:** † Department of Chemical Engineering, 34912 National Cheng Kung University, Tainan 701, Taiwan; ‡ Department of Computer Science, 34890 Tunghai University, Taichung 407, Taiwan; § Department of Medical Research, Kuang Tien General Hospital, Taichung 433, Taiwan; ∥ Research Center for Smart Sustainable Circular Economy, Tunghai University, Taichung 407, Taiwan; ⊥ Department of Chemical and Material Engineering, Tunghai University, Taichung 407, Taiwan; # Department of Chemical Engineering and Materials Science, Yuan Ze University, Chung-Li 302, Taiwan

## Abstract

Protein language models have achieved remarkable success in structure prediction and *de novo* design. However, their understanding of physical interactions remains limited. For structure-based models, we hypothesize that this limitation stems from the absence of explicit solvent modeling: water mediates hydrogen bonding, hydrophobic burial, and electrostatic interactions that govern protein thermodynamics and kinetics, yet current structure-based models treat proteins as if they exist in a vacuum. Using thermophilic protein classification as a proxy task for force-field awareness, per-residue logit mis-correlated with H-bonds as a destabilizing attribute and exhibits significant solvent-accessible surface area (SASA)-dependent error, despite high overall classification accuracy. These artifacts point to the water–protein interface as a source of systematic bias. To address this, we propose HEIWA (HybridExplicit-Implicit Water Attention), a novel attention module that injects solvent-mediated force-field information into static ESM3 residue embeddings via an all-query-to-all-key water–residue attention. Biophysics-guided model selection identifies configurations whose residue-level outputs are more consistent with the RMSF benchmark and selected biophysical attributes, while classification performance remains broadly comparable to the baseline. By forcing the model to predict the protein thermophilicity from the contributions of individual residues, HEIWA learns biophysical patterns that can be extended to SUPFAM absent from the training data set. HEIWA also largely preserves the embedding structure of ESM3. Molecular dynamics simulations show improved kinetic stability under thermophilic conditions, suggesting the potential to generate structures with greater kinetic stability. To our knowledge, this is the first study to incorporate explicit solvent encoding into a residue-resolution protein language model.

## Graphical abstract

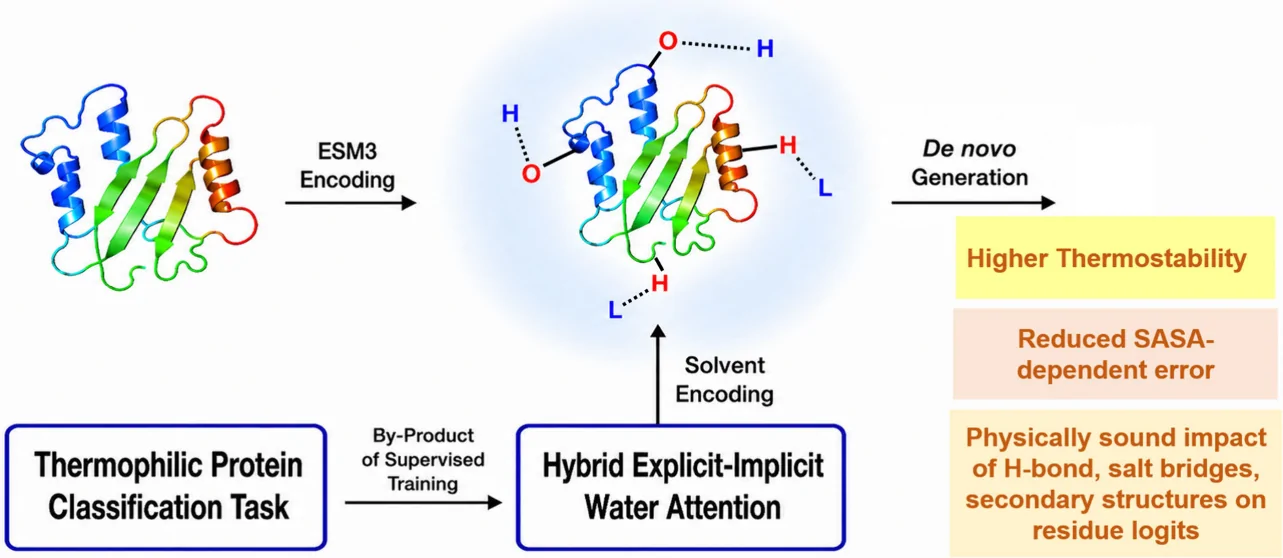

## Introduction

Machine learning has driven transformative advances across a range of scientific domains, from materials discovery,^1^ natural language processing, medical imaging, and genomics.^2^ Biochemical engineering, which uses proteins as biocatalysts, has been an attractive approach in chemical engineering due to their high substrate selectivity, mild reaction conditions, and excellent biocompatibility.^3^ Given the complexity of protein engineering, *in silico* modeling has been a desirable approach to enhancing the precision of protein design. Since protein structures involve complex interactions between residues and with the physicochemical environment, deep-learning models are increasingly used to learn these relationships and guide protein engineering. To date, transformers^4^ remain the cornerstone architecture for constructing protein deep learning models,^5–10^ as in natural language models. These protein language models have notably unlocked various groundbreaking achievements, such as protein structure prediction,^11–14^ protein–ligand structure prediction,^15,16^ protein–protein interactions,^17–20^ ligand generation from protein sequences,^21^ multiplex conformation sampling for single peptide sequences,^22–24^ genome design,^25^ and associations between sequence features and keyword prompts,^26–29^ customizable protein generation,^30–32^ exploration of novel nonribosomal peptide synthetase^33^ and enzymes.^34^

Despite these impressive advances, significant limitations remain in the current generation of the protein language models. While transformer architectures excel at learning sequence patterns from large databases, they still model physical molecular interactions with limited fidelity. To the best of our knowledge, open-source physics-based tools have not yet been replaced by transformer-based models.^33–35^ Open-source megamodels have yet to demonstrate limited robustness toward orphan sequences.^36–39^ These symptoms indicate a lack of true physical awareness, despite analyzing protein sequences as a language.

Fundamentally, protein sequences comprise far less token diversity than human languages, which makes positional embeddings disproportionately important when coordinate-informed geometric reasoning is absent. This limitation was recently demonstrated by Zhang et al.,^40^ who used a single-residue perturbation experiment to show that ESM2’s attention concentrated on evolutionarily conserved residues, where ESM2’s residue–residue contact predictions relied largely on recognizing local motifs. Although hydrophobic-residue conservation was observed, it was largely confined to conserved motifs,^41^ making its physical awareness questionable.

Recently, several generative protein structure prediction models encoded protein structure as all-atom graph representations,^15,42–44^ thereby allowing biophysical interactions, such as hydrogen bonds and salt bridges, to be tunable. However, these models either do not model solvent molecules or do not capture the causal relationship in solvent–protein codynamics due to the static, relaxed nature of PDB structures, although solvent critically shapes the energy landscape of protein folding and unfolding, and hence governs protein thermodynamics and kinetics.^45^

In this study, we investigate a critical question in computational biology: ***Does knowledge of solventless protein structure alone imply an understanding of protein physics in AI models?*** We postulate that the physical awareness of a protein model consists of two distinct components: geometry awareness and force field awareness (Figure 1a). Geometry awareness refers to knowledge of accurate atomic coordinates, either provided as input or estimated from the amino acid sequence identity and knowledge of the forces exerted by residues. Force-field awareness refers to knowledge of the forces acting on a residue as a result of its interactions with other atoms when the amino acid sequence identity and residue coordinates are given (Figure 1a). We chose ESM3 ^27^ as the target of study. ESM3 uses a coordinate-informed protein encoding to extend tokenization beyond individual amino acids to include secondary structure, solvent-accessible surface area (SASA), local residue cluster geometry, and externally supplied functions, allowing for a useful coexistence of multiple tracks of token unmasking. While its coordinate-informed residue encoding is designed to support geometry awareness (Figure 1d), we hypothesize that without explicit solvent modeling, it may have limited force-field awareness. This hypothesis is consistent with recent reports of poor performance of ESM3 in predicting protein melting temperatures, Gibbs free energy change by mutations, and multiple discussions of the apparent instability of ESM3-generated proteins.^46,47^

**Figure 1:**
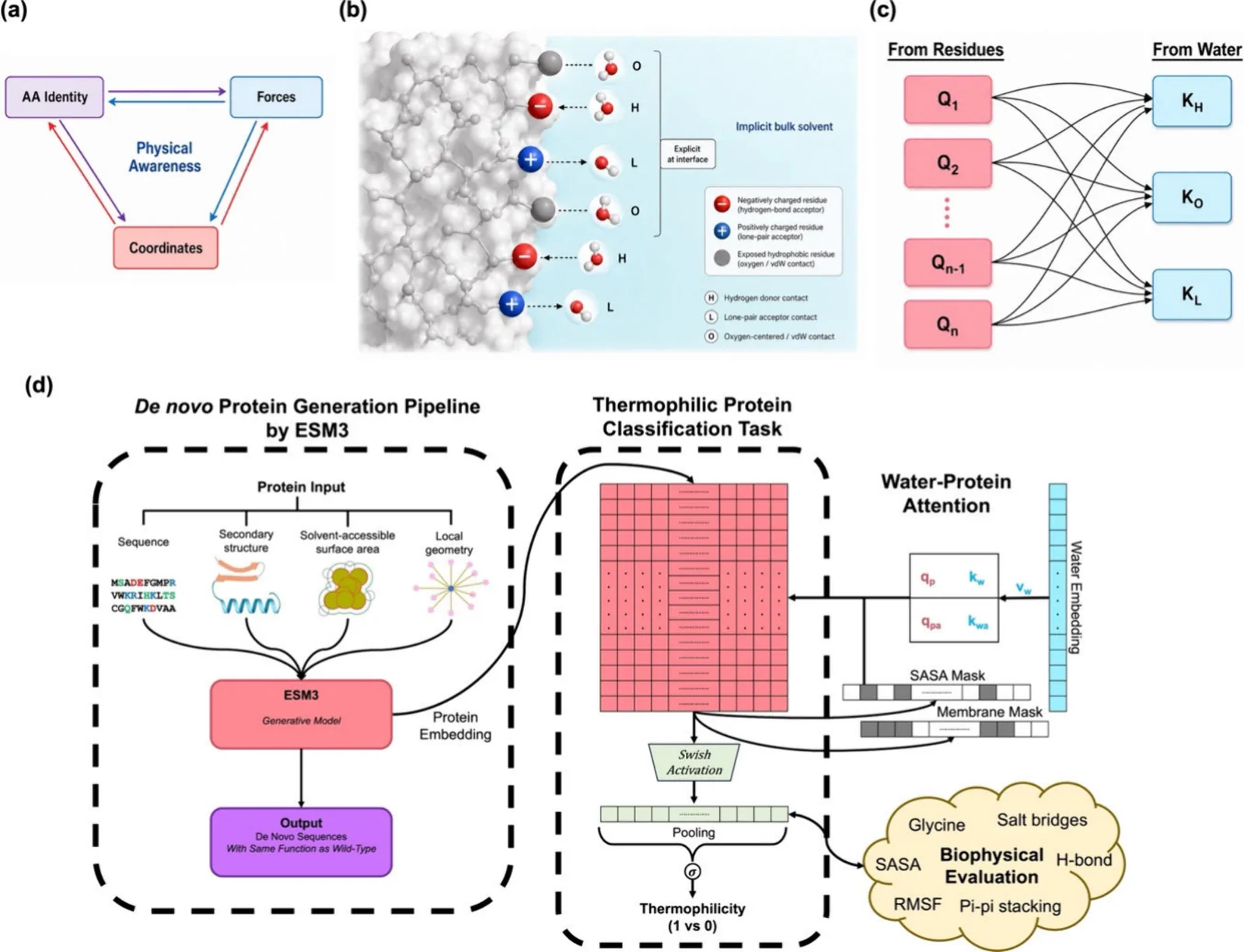
Conceptual overview. (a) A concept diagram illustrating that a self-contained physical model should capture force-field interactions between residues given the coordinates and residue identities. (b) Illustration of the proposed explicit-implicit water attention mechanism. (c) All-to-all water–protein attention head design. (d) Full HEIWA model architecture integrated into the thermophilic protein classification task and the ESM3 *de novo* protein generation pipeline.

In this study, we use a thermophilic protein classification as a proxy task to demonstrate the impact of vacuum bias, arising from the absence of explicit solvent modeling on the physical awareness of ESM3. Finally, we propose HEIWA (Hybrid Explicit-Implicit Water Attention), a novel attention module that injects solvent-mediated force-field information into ESM3 residue embeddings (Figure 1b–d). Molecular dynamics simulations demonstrate the positive impact of solvent correlations on ESM3-generated proteins. Herein, our results suggest that solvent encoding in a residue-resolution protein language model can improve its physical understanding.

## Results and Discussion

### Thermophilic Protein Classification Using ESM3 Embeddings Suffers from Imperfect Biophysical Causation and SASA-Dependent Error

We performed a thermophilic homologue classification task using the model architecture shown in Figure 1d. In brief, the embeddings of each residues, excluding the CLS and EOS, were nonlinearly processed by two Swish-activated^49^ feed-forward layers to produce a scalar logit per residue, and then average-pooled and passed through a sigmoid function to produce the final output. The scalar logit per residue represents each residue’s contribution to protein thermophilicity. The number of intermediate dimensions would be optimized as a hyperparameter. Since protein stability is sensitive to single-point mutations, we ensure all positive and negative samples have similar sequences by using a data set comprising 5125 homologous sequences, excluding acidophilic, alkaliphilic, piezophilic, and halophilic proteins, including both thermophilic and nonthermophilic proteins (see data filtering criteria in Table S1). The data were first split into training, validation, and test sets using SUPFAM stratification; and later repeated using a SUPFAM-grouped data set.

The rationale of this design is to force the model to predict protein thermophilicity based on the contribution of individual residues. This design creates a discrepancy between the backpropagation objective and the learning objective since only whole-protein thermophilicity is used as the label for backpropagation. Therefore, additional measures must be used to ensure the per-residue latent outputs are physically meaningful. Accordingly, in addition to evaluating model performance using metrics including accuracy, F1, sensitivity, specificity, and precision, we also perform biophysics-guided model selection based on the scalar logit per residue (Figure 1d). To this end, a root-mean-square fluctuation (RMSF) evaluation benchmark based on 70 mesophilic proteins (54 of which were not included in our data set), each from a unique SUPFAM, was constructed using molecular dynamics simulation at 310 K. For each structure, positions of H-bonds, salt bridges, secondary structure (B, E, G, and H), glycine, and π–π stacking are identified as described in Materials and Methods. This yielded an evaluation benchmark containing residue-wise RMSF values and their correlations with biophysical attributes. Since RMSFs are non-negative numbers while model logits can be negative, a nonparametric Spearman correlation would be suitable to measure the consistency between them. One potential caveat is that the per-residue logit produced by the model may represent the RMSF or stabilizing forces, or a statistical mix of both qualities. In the 70-protein benchmark, salt bridges and secondary structures are associated with significantly lower RMSF, while glycine is associated with significantly higher RMSF (Figure 2a). These findings are consistent with the stabilizing nature of salt bridges and secondary structures, and the flexibility-promoting properties of glycine. However, H-bonds, a stabilizing attribute, are not associated with a significant change in RMSF during the simulations, possibly due to the H-bond-mediated interaction with solvent molecules enhancing the mobility of surface residues (Figure 2c). In our evaluation, we excluded only model hyperparameters that incorrectly identified H-bonds as a destabilizing attribute. For each model whose logits showed correlation with biophysical attributes consistent with the RMSF benchmark, we further assessed the model logits’ correlation with RMSF and SASA.

**Figure 2:**
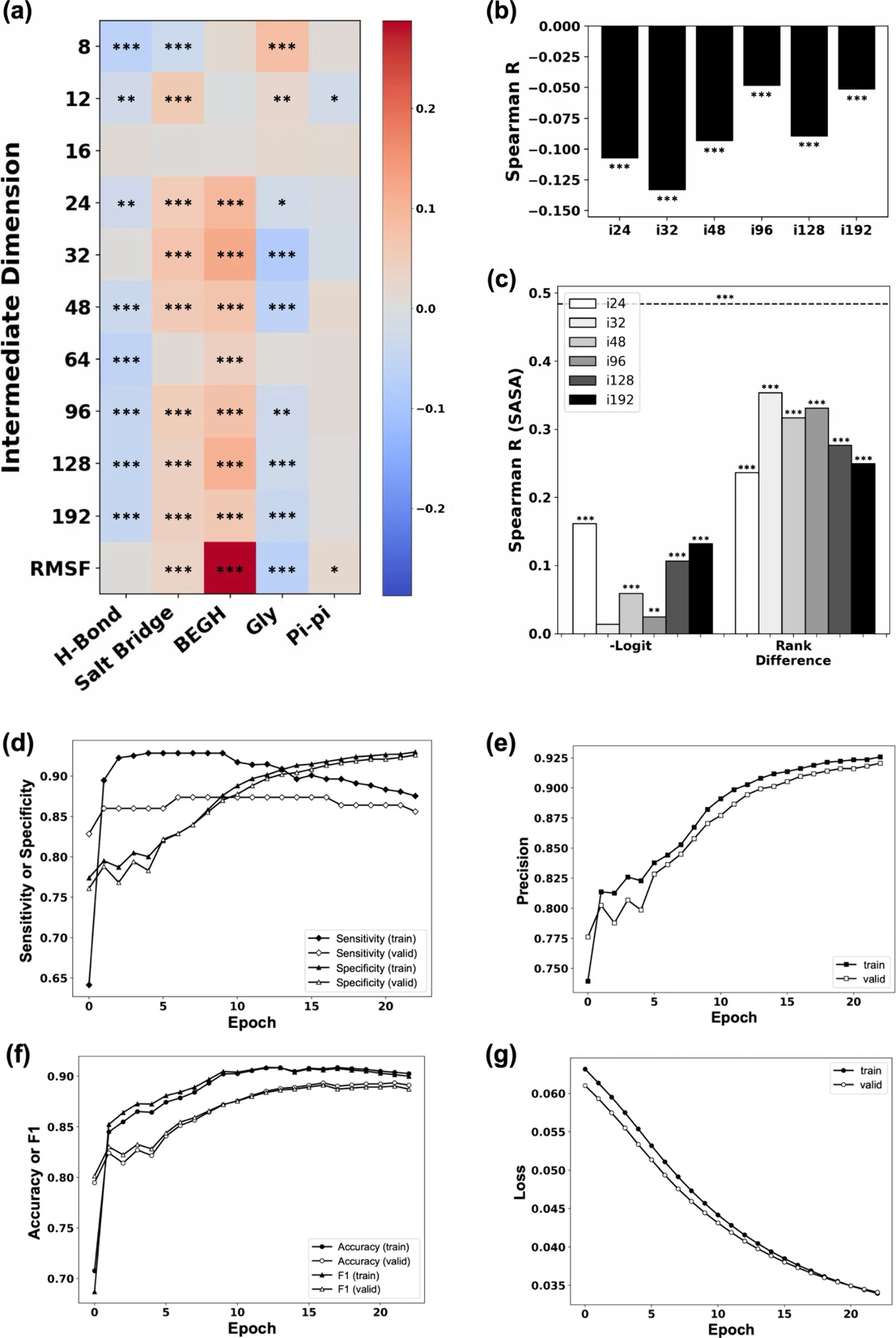
Performance of the baseline ESM3 thermophilic classifier. (a) Spearman correlations between per-residue logits and biophysical attributes (RMSF, H-bond count, SASA, hydrophobicity) with different intermediate dimensions. Red indicates a correlation that suggests a positive contribution to protein thermophilicity; blue indicates a correlation that suggests a negative contribution. (b) Logit-RMSF correlations are shown for hyperparameter configurations with biophysical consistent correlations, except for H-bonds. (c) SASA-RMSF correlation is indicated by a dotted line. The correlation between SASA and per-residue logits, along with the rank difference from SASA-RMSF, is shown. This SASA-dependent error reflects systematic overestimation of the stability of surface residues. (d–g) Training and validation learning curves of the selected 96-dimensional baseline. For statistical significance: *p < 0.05, **p < 0.01, ***p < 0.001.

These analyses were essential (Figure 2a) to reveal critical failures. First, regardless of intermediate dimension, H-bonds are systematically misidentified as destabilizing attributes, contrary to established biophysics and our RMSF benchmark. For models with fewer than 96 dimensions, glycine – a helix-destabilizing and flexibility-promoting residue – is misidentified as a stabilizing attribute. This helps disqualify the high F1 score of the 8-dim model (Table 1). Figure 2c demonstrates that prediction error strongly depends on SASA (solvent-accessible surface area): the mobility of surface residues, which are more mobile (elevated RMSF) due to codynamics with the highly mobile solvent molecules,^50,51^ is systematically underestimated.

**Table 1:** Thermophilic Classification Performance of ESM3 Baseline with and without the HEIWA Module for Hyperparameter Configurations That Exhibited Sound Biophysical Correlations^a^

Hyperparameter	Data set	Acc.	F1	Prec.	Sens.	Spec.	Loss
Baseline Model							
24-dim	Train	0.7803	0.8136	0.7063	0.9594	0.6011	0.0584
	Valid	0.7658	0.8013	0.6957	0.9447	0.5868	0.0568
	Test	0.7628	0.7975	0.6956	0.9346	0.5910	0.0521
32-dim	Train	0.8746	0.8763	0.8649	0.8880	0.8613	0.0596
	Valid	0.8557	0.8561	0.8539	0.8582	0.8531	0.0580
	Test	0.8603	0.8608	0.8574	0.8643	0.8563	0.0534
48-dim	Train	0.8536	0.8666	0.7960	0.9509	0.7563	0.0514
	Valid	0.8358	0.8498	0.7831	0.9290	0.7428	0.0499
	Test	0.8245	0.8367	0.7821	0.8994	0.7495	0.0455
96-dim	Train	0.9048	0.9040	0.9118	0.8964	0.9132	0.0394
	Valid	0.8890	0.8873	0.9013	0.8737	0.9043	0.0389
	Test	0.8842	0.8808	0.9077	0.8555	0.9130	0.0351
128-dim	Train	0.9070	0.9082	0.8968	0.9200	0.8941	0.0417
	Valid	0.8832	0.8830	0.8844	0.8816	0.8848	0.0408
	Test	0.8722	0.8700	0.8850	0.8555	0.8888	0.0369
192-dim	Train	0.9069	0.9052	0.9225	0.8886	0.9253	0.0352
	Valid	0.8909	0.8879	0.9130	0.8640	0.9177	0.0348
	Test	0.8905	0.8865	0.9198	0.8555	0.9254	0.0316
With HEIWA Module							
Head4_v32_vc0.4	Train	0.9183	0.9165	0.9371	0.8969	0.9398	0.0101
	Valid	0.8847	0.8846	0.8855	0.8837	0.8858	0.0144
	Test	0.8763	0.8748	0.8856	0.8643	0.8884	0.0132
Head4_v32_vc0.6	Train	0.8993	0.8968	0.9197	0.8750	0.9236	0.0118
	Valid	0.8829	0.8830	0.8823	0.8837	0.8821	0.0152
	Test	0.8771	0.8755	0.8870	0.8643	0.8899	0.0133
Head4_v128_vc0.8	Train	0.9115	0.9101	0.9252	0.8954	0.9276	0.0112
	Valid	0.8862	0.8859	0.8881	0.8837	0.8887	0.0176
	Test	0.8796	0.8778	0.8917	0.8643	0.8950	0.0150
Head4_v192_vc0.4	Train	0.9270	0.9256	0.9437	0.9082	0.9458	0.0103
	Valid	0.8796	0.8792	0.8825	0.8758	0.8834	0.0232
	Test	0.8811	0.8802	0.8874	0.8731	0.8892	0.0213
Head4_v192_vc0.8	Train	0.9183	0.9164	0.9382	0.8956	0.9410	0.0102
	Valid	0.8916	0.8907	0.8979	0.8837	0.8995	0.0191
	Test	0.8848	0.8824	0.9012	0.8643	0.9053	0.0166
Head5_v128_vc0.2	Train	0.9106	0.9084	0.9309	0.8870	0.9341	0.0111
	Valid	0.8717	0.8722	0.8686	0.8758	0.8676	0.0174
	Test	0.8852	0.8858	0.8810	0.8906	0.8797	0.0156

a All models were trained on the SUPFAM-stratified dataset.

On the basis of these observations, we hypothesize that the absence of solvent–protein interaction information is the main cause of prediction error: water mediates hydrophobic burial and electrostatic interactions at the protein surface; its omission may therefore introduce systematic errors at the protein–solvent interface.

### Encoding Water–Protein Interactions Using a Hybrid Explicit–Implicit Water Attention (HEIWA) Module

HEIWA is designed to inject solvent-mediated force field information into the ESM3 embedding through a protein–water attention pipeline as a trainable attention module (Figure 1d). Since solvent molecules interact with each residue over time from multiple directions and positions, we use the SE(3)-equivariant ESM3 embedding^27^ to train a static, learnable water embedding that represents a homogeneous field of solvent. Under this implicit assumption, we encode the impact of the solvent using single-shot message passing.

To capture the benefits of explicit water encoding without defining coordinates for individual solvent molecules, we define distinct points of interaction at the protein–solvent interface, while treating all solvent molecules beyond that implicitly (Figure 1b). Mathematically, we represent different types of solvent–protein interactions using distinct water key vectors with multiple attention heads to capture the benefits of explicit water encoding. Intuitively, we define at least three types of water keys: *k*_*wh*_ (hydrogen), *k*_*wl*_ (lone-pair), and *k*_*wo*_ (oxygen). Conceptually, the *k*_*wh*_ interacts with exposed lone-pair acceptors and negatively charged residues; the *k*_*wl*_ interacts with exposed hydrogen-bond donors and positively charged residues; and the *k*_*wo*_ mediates interactions with exposed hydrophobic residues. A sparsity constraint is applied to the attention scores of the: “*h*” and “*l*” heads, preventing them from attending to the same residue query, thereby enforcing an antagonistic relationship between H-bond donors and acceptors, consistent with water chemistry. The sparsity loss can be computed directly as the product of their attention scores.

The standard scaled dot-product attention^4^ governs intertoken message passing via a softmax-normalized score, which distributes attention weights competitively across all tokens such that each head is effectively forced to select a dominant token. Since solvent tokens are duplicated and the desired signal are dominated by the time-averaged solvent–protein attraction forces, we replace the softmax activation with a more linear alternative. Retaining only positive signals is therefore physically motivated. Following Wortsman et al.,^48^ we employ ReLU-activation, which sets neative scores to zero and allows a single solvent head to attend to multiple residues simultaneously, which is appropriate when a solvent molecule can interact with all surface residues at once. One practical benefit is that ReLU-based attention does not require the $\sqrt{d_{qk}}$ scaling that softmax needs for numerical stability.

Moreover, we explore models with 2 (*k*_*wo*_ removed), 3, 4 (two *k*_*wo*_ heads), and 5 solvent heads. In the five-head configuration, we introduce an additional antagonistic pair *k*_*wna*_ (sodium) and *k*_*wcl*_ (chloride). A bias term, analogous to that in the van’t Hoff isotherm, is applied to the attention scores of these heads, according to the concentration *c* of water (55.5 M) and physiological salt (0.15 M). The van’t Hoff isotherm is therefore simplified as follows:

$$a=\mathrm{ReLU}\left(q_{p}\cdot k_{w}+\ln\left(c\right)\right)$$

where ln­(*c*) is referred to as the Le Chatelier bias in the repository.

#### Rotational Attention

One issue to consider is that solvent molecules do not necessarily approach a residue at a fixed angle relative to the Gram-Schmidt coordinate frame of each residue.^27^ The solvent may need to rotate to achieve a favorable orientation for interaction with the residue. Since solvent molecules move stochastically and are influenced by multiple residues, the resulting orientation may not maximize the attractive forces. To this end, we introduce a rotational attention score to capture the angular dependence of interatomic forces: interactions are strongest at the optimal interaction angle and diminish as the geometry departs from it. The rotational attention *a*_*r*_ is calculated by the dot product between the 3D angle representation of the protein *q*_*pa*_ and the solvent field *k*_*wa*_, followed by a sigmoid activation, where *b*_*p*_ and *b*_*w*_ represent the atoms’ tolerance for deviation from the optimal contact angle.

$$a_{r}=1-\sigma \left(\frac{q_{pa}\cdot k_{wa}}{\sqrt{3}}+b_{p}+b_{w}\right)$$

The final attention score is calculated as the product of the ReLU-activated vanilla attention and the rotational attention score. A strong signal is produced only when both interaction strength and geometric compatibility are high.

$$a^{\prime }=a\cdot a_{r}$$

#### All-to-All Attention and Swish-Activated LoRA (SoRA) for Output Compression

As each residue generates a set of queries *q*_*p*_ and rotational queries *q*_*pr*_, representing the various interacting atoms within the residue, while every protein query should be free to interact with all possible water keys *k*_*w*_, we designed an all-*q*-to-all-*k* attention framework (Figure 1c). Using a DSSP-generated data set in which all exposed H-bond donors and acceptors were labeled, we predetermined the number of protein heads as 3 and fixed the dimension of *q*_*p*_ at 128. The all-to-all framework introduces substantial redundancy in the dimensions of the attention output, since many query–key combinations encode overlapping interaction types. To reduce this redundancy before the downstream SwiGLU feed-forward, we project the attention output *o* through a bottleneck we call SoRA (Swish-gated low-rank adaptation), drawing on two complementary ideas from the literature.

The Swish activation^49^ is a sigmoid-gated linear unit that introduces soft learnable selectivity into a projection. Recent work showed that applying a gated mechanism directly to the attention output projection can improve model training stability, alleviate the attention sink problem, tolerate higher learning rates, and improve robustness under model scaling.^52^

Swish(x)=Mx·σ(Nx)

On the other hand, low-rank adaptation (LoRA)^53^ demonstrated that large weight matrices can be decomposed into low-rank products to compress the adaptation updates, with the rank acting as a regularizer that controls how much new information is injected into a frozen representation.

$$\Delta W=ABx,\left(B\in R^{d\times r},A\in R^{r\times d},r<d\right)$$

SoRA combines these two ideas: the attention output is projected through a Swish-gated low-rank bottleneck. The value compression factor vc ∈ {0.2, 0.3, 0.4, 0.6, 0.8} controls the dimension of the water message injected into the ESM3 embedding. A smaller vc retains only the strongest, most consistent solvent signals; a larger vc permits a richer but potentially noisier injection.

$$\begin{array}{l} \mathrm{SoRA}\left(x\right)=\mathrm{Swish}\left(x\right),\left(M\in R^{d\times r},N\in R^{d\times r},r<d\right)\\ o=\mathrm{SoRA}\left(\mathrm{ReLU}\left(a^{\prime }\right)\cdot V\right)\\ \mathrm{message passing}=\mathrm{SwiGLU}\left(o\right) \end{array}$$

Finally, to mask solvent interactions with buried residues and membrane-phase residues-which are physically shielded from bulk solvent-we pretrained two classifiers: a SASA-based surface-identification classifier (Figure S1) and a membrane-phase identification classifier (Figure S2). These gates produce sigmoid-activated values that can selectively mask the final water message passing before the eventual modification of the ESM3 embedding. Unlike self-attention in language processing models, solvent-to-protein message passing occurs after the SwiGLU feed-forward layer; therefore, these gates are applied after the SwiGLU network.

### HEIWA Restores Biophysical Soundness after Hyperparameter Optimization

Using the 96-dim model as the baseline, we performed hyperparameter tuning of the HEIWA module, including the dimension of the value vector and the SoRA compression factor vc. Tuning was performed separately for four solvent heads counts (2, 3, 4, 5). Our data showed that models with 2 and 3 solvent heads are insufficient to resolve the H-bond destabilization and glycine stabilization problems. However, with 4 or 5 heads, both artifacts can be resolved (Figure 3a), a correction that was not achieved by the solvent-free baseline.

**Figure 3:**
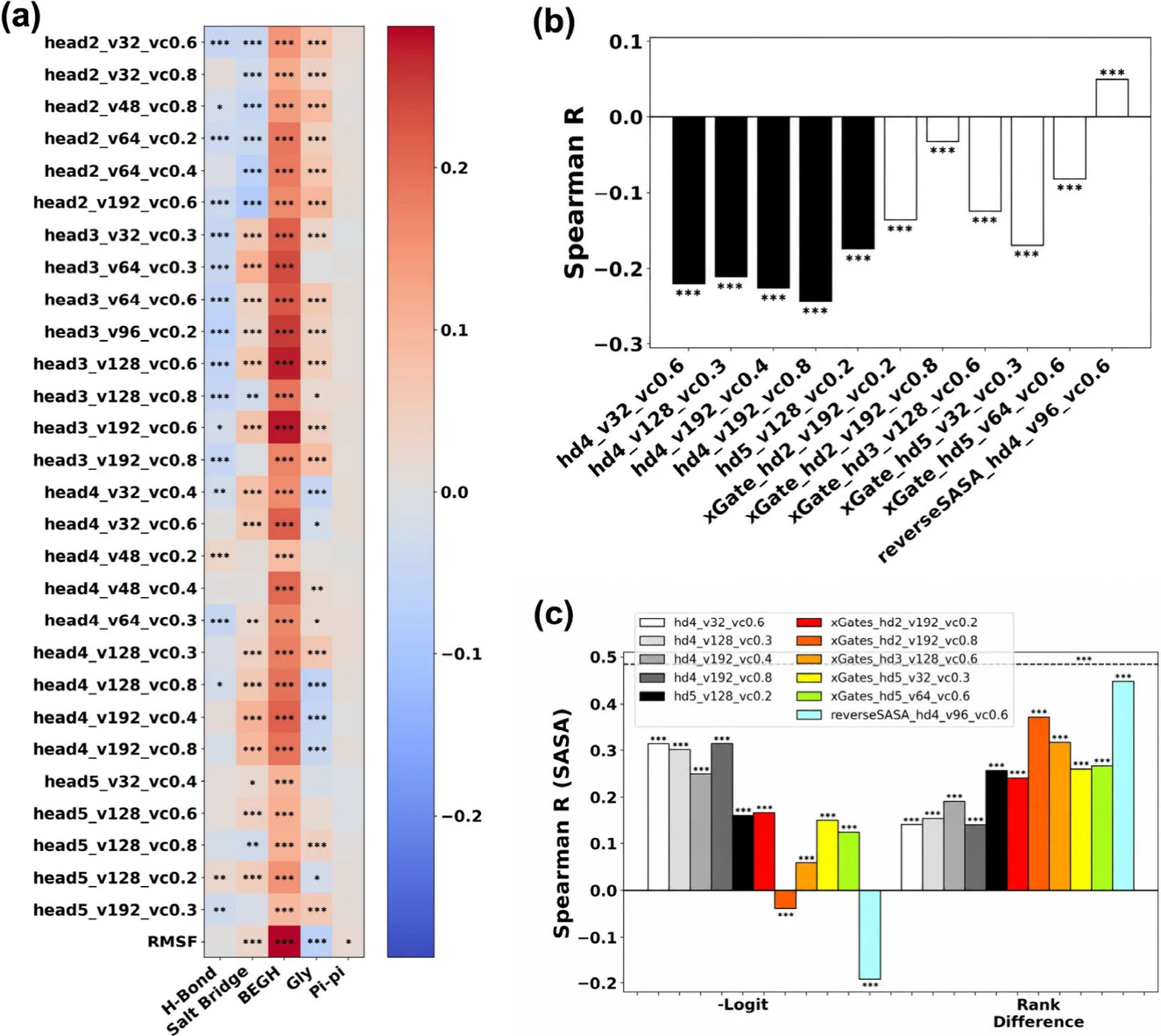
Performance of the thermophilic classifier with the HEIWA module. (a) Spearman correlations between per-residue logit and biophysical attributes (RMSF, H-bond count, SASA, hydrophobicity) across different hyperparameter configurations. Value-vector dimensions are indicated by “v”, and SoRA compression factor by “vc”. Red indicates a correlation that suggests a positive contribution to protein thermophilicity; blue indicates a correlation that suggests a negative contribution. (b) Logit-RMSF correlations are shown for hyperparameters configurations with biophysically consistent correlations. (c) SASA-RMSF correlation is indicated by a dotted line. The correlation between SASA and per-residue logits, along with the rank difference from SASA-RMSF, is shown. For statistical significance: *p < 0.05, **p < 0.01, ***p < 0.001.

Using the RMSF benchmark, we selected 4 out of 120 models that showed improved correlation between per-residue logit and RMSF (Figure 3b) and SASA (Figure 3c), indicating greater consistency with residue mobility. Based on this analysis, we selected the HEIWA hyperparameter of 4 solvent heads, *d*_*v*_ = 192, and vc = 0.8, with learning curves shown in Figure 4a–d. HEIWA yielded higher prediction confidence (Figure 4e), consistent with lower loss (Table 1). Overall, the biophysical consistency of ESM3 embedding has been significantly improved.

**Figure 4:**
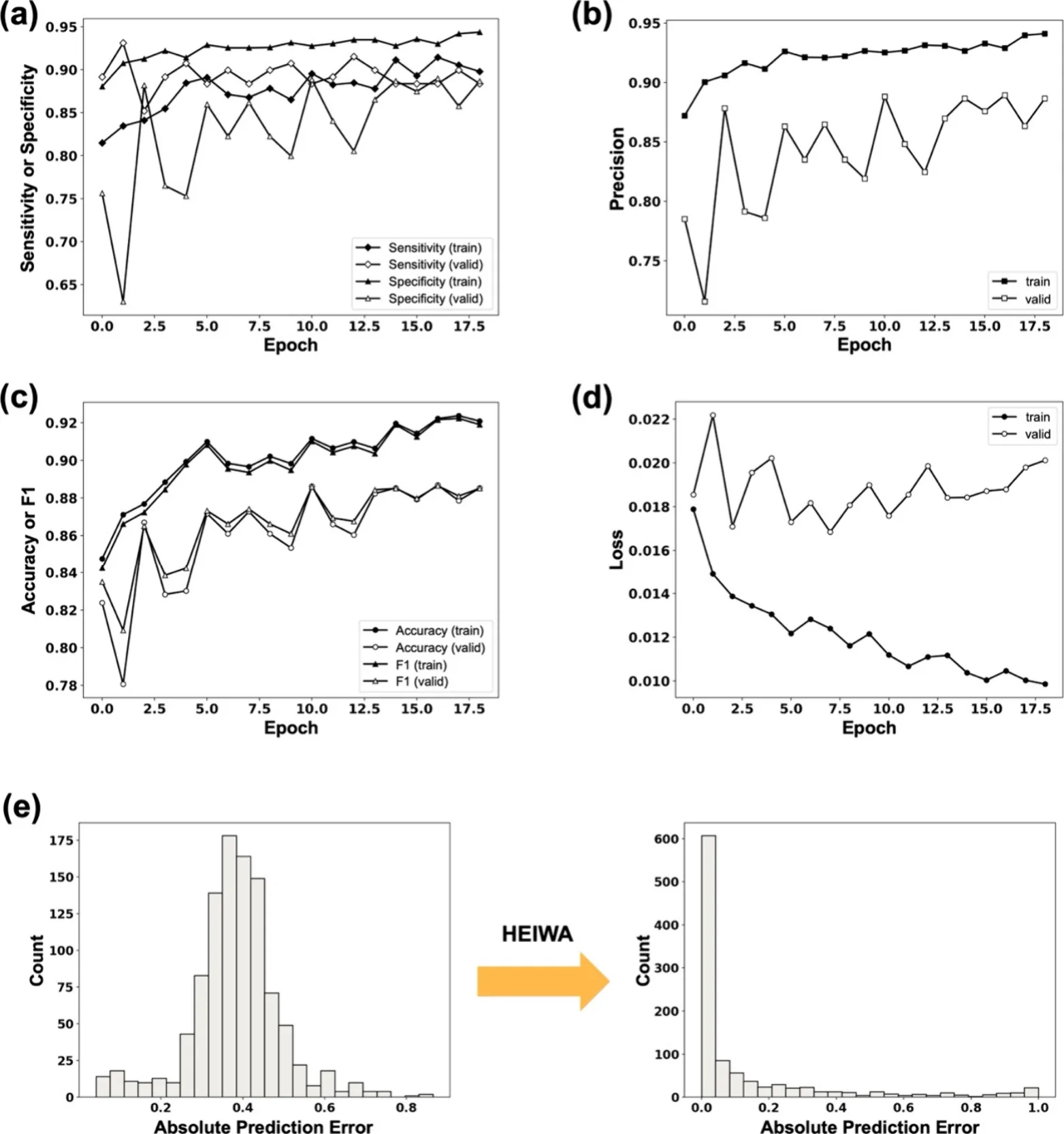
Model training and test performances of HEIWA. (a–d) Training and validation learning curves of the selected 96-dim baseline. (e) Comparison of the absolute prediction error on the test set between the 96-dim baseline and the HEIWA model. For statistical significance: *p < 0.05, **p < 0.01, ***p < 0.001.

Interestingly, switching to using the 48-dim baseline model yielded no hyperparameter configuration with sound biophysical behavior (Figure S3), suggesting that HEIWA increases the intermediate dimension requirement of the baseline model, such that 96 dimensions are needed to provide the capacity to encode both residue–residue and solvent–residue interactions.

### Strict Early-Stopping Criteria Can Be Used to Ensure Biophysical Soundness

Since the learning curves in Figure 4a–d appeared incomplete, we repeated the model training using a more permissive early stopping criterion: the minimum value of F1 and precision. This produced more complete learning curves (Figure 5c–f), but also disrupted biophysical understanding in baseline models with over 64 intermediate dimensions (Figure 5a). When HEIWA was added to the 96-dim model, no hyperparameter configuration exhibited sound biophysical understanding (Figure 5b). This is consistent with the discrepancy between protein thermophilicity as the training label and the contribution of individual residues as the learning objective. As shown in Table 1, the chosen HEIWA model “Head4_v192_vc0.8” showed no notable improvement in classification metrics compared to the 96-dim baseline, apart from the loss. This suggests that such a discrepancy can be managed with earlier stopping at the expense of the backpropagation objective.

**Figure 5:**
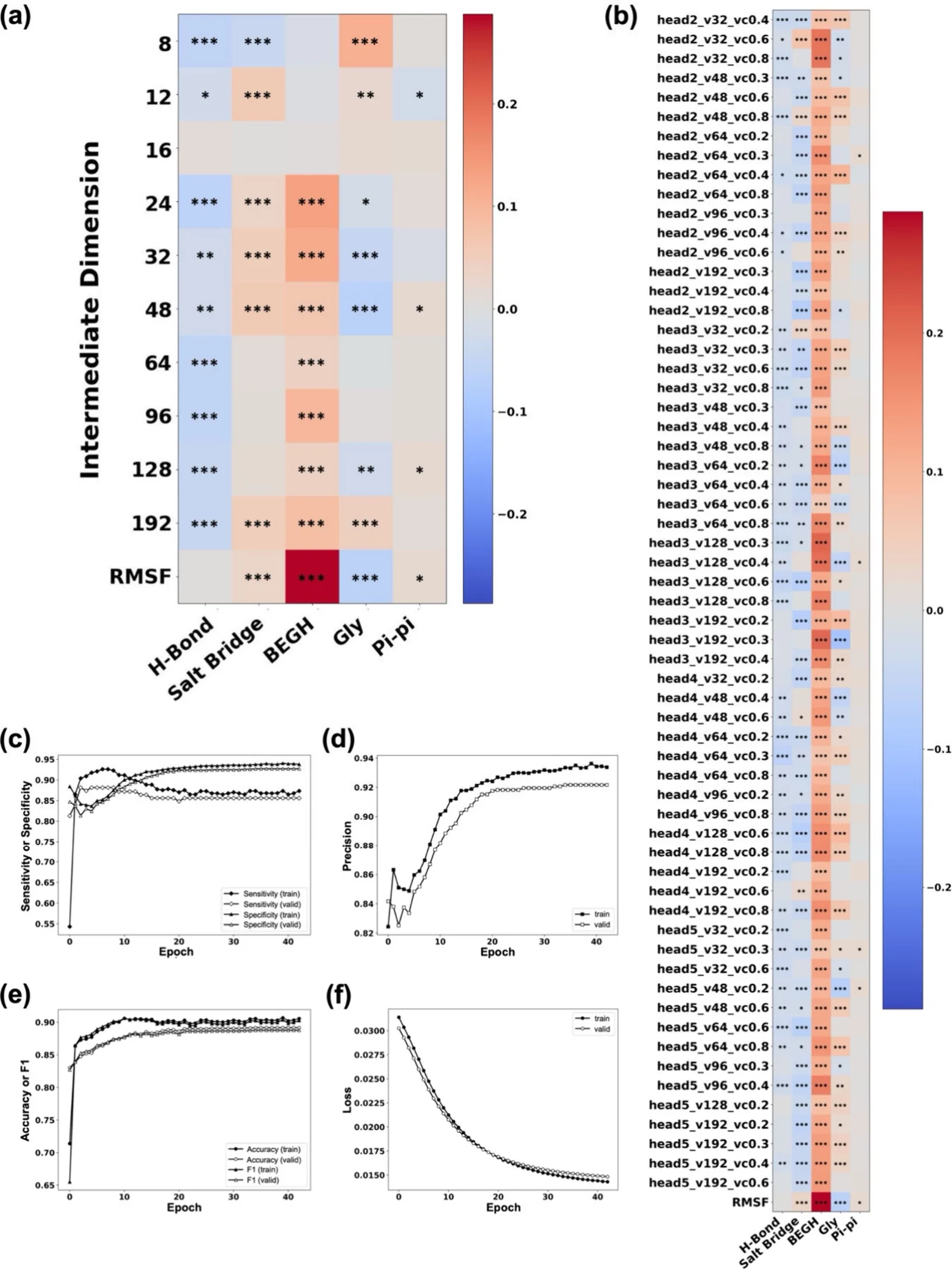
Using more permissive early stopping criteria using the minimum value of F1 and precision disrupted biophysical correlations. Compared with the baseline ESM3 thermophilic classifier (a), this disruption was more pronounced with the HEIWA module (b). (c–f) Learning curves for the 96-dim baseline model are shown. See the figure caption inFigure [Fig fig3].

### Ablation of SASA and Membrane Gates Results in Lower Correlation with RMSF and SASA

To test whether a solvent–protein attention module can best address the physical errors in the baseline thermophilic protein classifier models, we ablated the SASA gate and membrane gates. These gates ensure that only exposed aqueous-phase residues receive messages from the HEIWA module. Interestingly, 5 of 120 hyperparameters passed the biophysical correlation test (Figure S4a), including “Head5_v64_vc0.2”, “Head5_v32_vc0.3”, “Head3_v128_vc0.6”, “Head2_v192_vc0.8”, and “Head2_v192_vc0.2”. However, correlating the per-residue logit with RMSF (Figure 3b) and SASA (Figure 3c) showed consistently weaker performance than those of HEIWA models. With the SASA gate reversed, one hyperparameter configuration, “Head4_v96_vc0.6”, passed our biophysics test (Figure S4b); however, the logit-RMSF (Figure 3b) and logit-SASA correlation (Figure 3c) had reversed signs. Reversing the membrane gate yielded no biophysically sound configuration; in all cases, H-bonds were misidentified as a destabilizing attribute (Figure S4c), similar to the baseline model. These findings indicate that the improved biophysical soundness due to adding the HEIWA module is not solely the result of an increased number of model parameters, but also reflects the selective solvent-to-protein message passing to exposed aqueous residues.

### Replacing HEIWA with Gaussian Random Vector Removes Biophysical Soundness

Similarly, we replace message passing by the HEIWA module, which was masked by SASA and membrane gate, with Gaussian random vectors generated from three random seeds. This eliminated biophysical soundness: both H-bonds and salt bridges were identified as destabilizing attributes (Figure S4a). This indicates that message passing facilitated by the HEIWA module is distinct from that produced by the Gaussian random parameters.

### Learning of Individual-Residue Stability Can Be Extended to Unfamiliar SUPFAMs

We have shown that adding HEIWA to a 96-dim baseline thermophilic protein classifier can help correct biophysical inconsistencies arising from the absence of solvent–protein interaction encoding, but with a limitation. Since model training was guided by whole-protein labels, it requires careful evaluation to determine whether the model learned individual residues’ contributions or instead learned a SUPFAM-specific summation requires careful proof (Figure 1d). This distinction has important implications for the use of a SUPFAM-stratified data set (Table S1), where representatives from each SUPFAM are present in all data partitions, potentially leading to data leakage. To this end, we repeat the same training task using a SUPFAM-grouped data set containing 399 SUPFAMs with both thermophilic and mesophilic proteins (Table S1). The training set, validation set, and test set comprised 239 SUPFAMs (3134 unique samples), 80 (1026 unique samples), and 80 (1056 unique samples), respectively.

Training the baseline classifiers resulted in a slight disruption of biophysical understanding, with only the 32-dim and 96-dim models showing sound understanding of salt bridges, secondary structures, and glycine (Figure 6a), despite H-bonds being still identified as destabilizing. While model loss and prediction metrics generally improved as the number of intermediate dimensions increased, with no evidence of performance deterioration in the validation and test data sets (Table 2), this comes with underperformance in the correlations with RMSF (Figure 6b) and SASA (Figure 6c). Both the 32-dim and 96-dim models showed a reversal in the sign of the correlation between the per-residue logit and SASA. This indicates a trade-off between improving the classification metrics and biophysical consistency.

**Figure 6:**
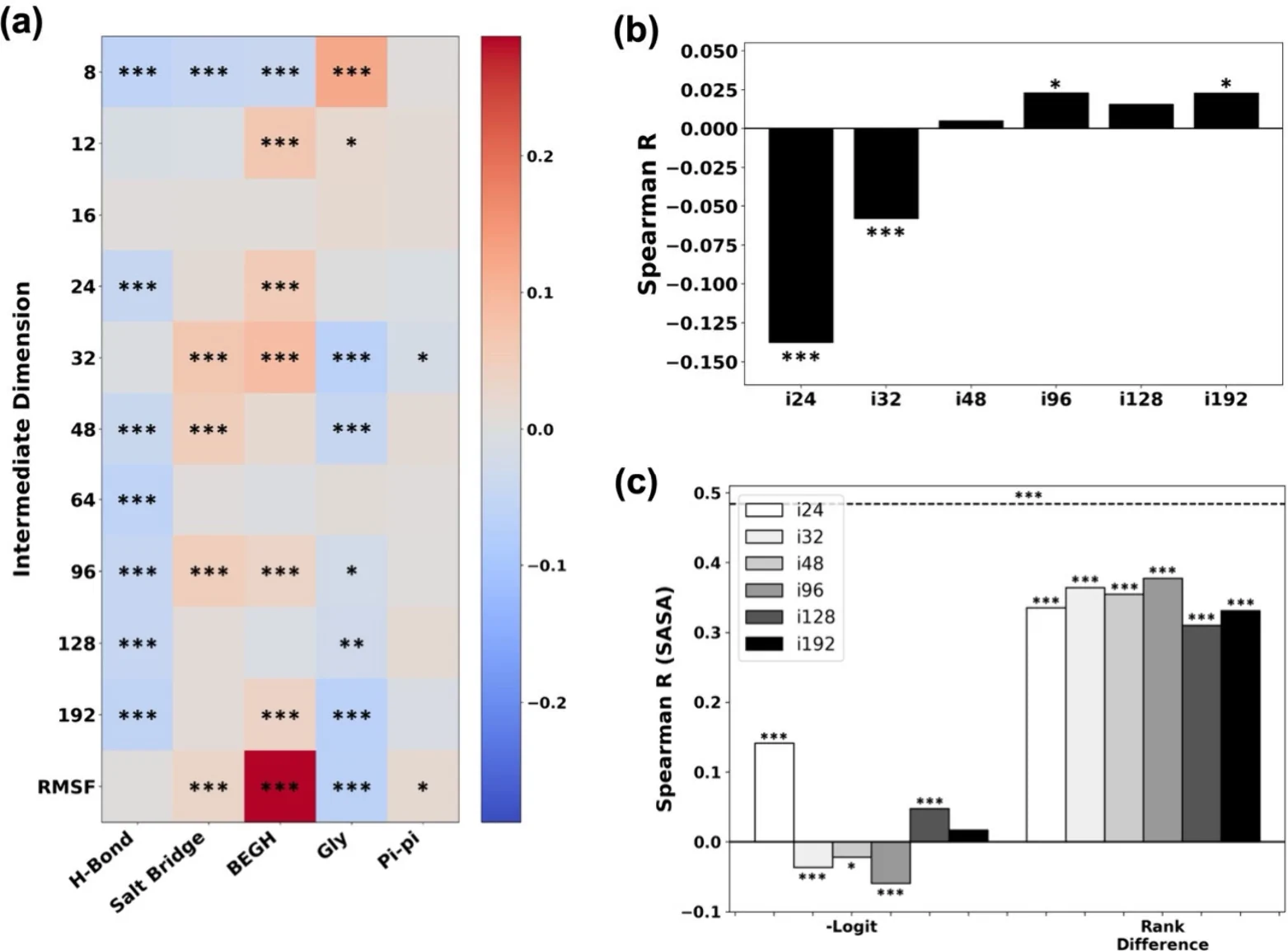
Performance of the baseline ESM3 thermophilic classifier trained on the SUPFAM-grouped data set. See the figure caption in Figure 2. Biophysical soundness was reduced relative to that shown in Figure 2a–c. As shown in Table 2, higher training performance in the models with more intermediate dimensions was associated with poorer correlation with (b) RMSF and (c) SASA.

**Table 2:** Thermophilic Classification Performance of ESM3 Baseline with and without the HEIWA Module for Hyperparameter Configurations That Exhibited Sound Biophysical Correlations^a^

Hyperparameter	Data set	Acc.	F1	Prec.	Sens.	Spec.	Loss
Baseline Model							
24-dim	Train	0.8043	0.8261	0.7431	0.93001	0.6784	0.0300
	Valid	0.8226	0.8392	0.7672	0.9262	0.7189	0.0333
	Test	0.7915	0.8174	0.7270	0.9335	0.64948	0.0314
32-dim	Train	0.8792	0.8776	0.8897	0.8658	0.8927	0.0301
	Valid	0.8856	0.8813	0.9154	0.8497	0.9214	0.0333
	Test	0.8834	0.8811	0.8990	0.8638	0.9030	0.0314
48-dim	Train	0.8388	0.8506	0.7925	0.9179	0.7597	0.0264
	Valid	0.8533	0.8601	0.8217	0.9023	0.8043	0.0292
	Test	0.8564	0.8658	0.8125	0.9266	0.7862	0.0275
96-dim	Train	0.8912	0.8909	0.8929	0.8889	0.8934	0.0219
	Valid	0.8935	0.8931	0.8967	0.8895	0.8976	0.0246
	Test	0.8947	0.8928	0.9085	0.8778	0.9116	0.0230
128-dim	Train	0.8785	0.8797	0.8711	0.8885	0.8686	0.0241
	Valid	0.8840	0.8839	0.8847	0.8830	0.8849	0.0267
	Test	0.8872	0.8869	0.8891	0.8847	0.8897	0.0251
192-dim	Train	0.8796	0.8802	0.8757	0.8848	0.8744	0.0230
	Valid	0.8832	0.8817	0.8935	0.8702	0.8962	0.0254
	Test	0.8841	0.8833	0.8890	0.8778	0.8904	0.0239
With HEIWA Module							
Head3_v128_vc0.4	Train	0.8999	0.8964	0.9288	0.8662	0.9336	0.0119
	Valid	0.8896	0.8878	0.9020	0.8740	0.9051	0.0162
	Test	0.8929	0.8907	0.9091	0.8731	0.9127	0.0140
Head4_v48_vc0.4	Train	0.9163	0.9144	0.9354	0.8943	0.9382	0.0108
	Valid	0.8905	0.8915	0.8837	0.8995	0.8816	0.0159
	Test	0.8780	0.8774	0.8817	0.8731	0.8828	0.0142
Head4_v64_vc0.6	Train	0.8912	0.8875	0.9192	0.8579	0.9246	0.0128
	Valid	0.8824	0.8786	0.9087	0.8504	0.9145	0.0153
	Test	0.8874	0.8837	0.9138	0.8555	0.9193	0.0129
Head4_v96_vc0.2	Train	0.9107	0.9086	0.9308	0.8875	0.9340	0.0122
	Valid	0.8894	0.8852	0.9208	0.8521	0.9268	0.0142
	Test	0.8950	0.8906	0.9288	0.8555	0.9344	0.0125

a All models were trained on the SUPFAM-grouped dataset.

Adding the HEIWA module restored biophysical understanding of 4 out of 120 hyperparameter combinations (Figure 7a), all of which produced per-residue logits that better correlate with RMSF (Figure 7b) and SASA (Figure 7c). Replacing HEIWA message passing with Gaussian random vectors resulted in no biophysical sound configuration among the hyperparameter combinations (Figure S5a). Ablating the SASA gates and membrane gates resulted in 4 of 120 hyperparameter combinations passing the biophysical test (Figure S5a), but these configurations consistently showed weaker correlations with RMSF (Figure 7b), and SASA (Figure 7c) consistently underperformed. Reversing the SASA gate or membrane gate resulted in no biophysically sound hyperparameter combinations (Figure S5b,c). Misidentification of H bonds as a destabilizing attribute was largely responsible for the physical unsoundness of models with the reversed membrane gate (Figure S5c).

**Figure 7:**
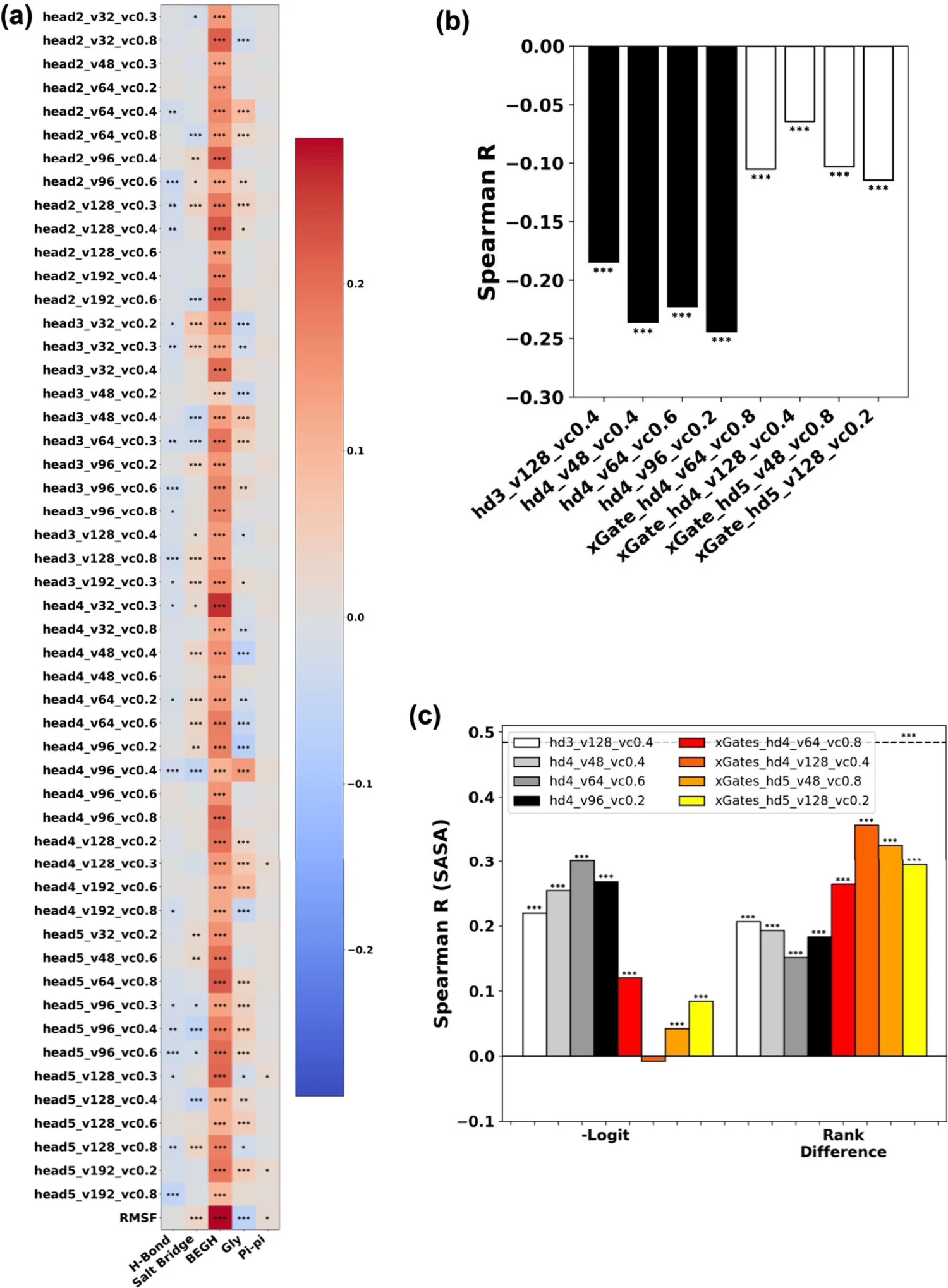
Performance of the thermophilic classifier with HEIWA trained on the SUPFAM-grouped data set. Biophysical soundness was reduced relative to that shown in Figure 3a–c.

These results are broadly consistent with those from the SUPFAM-stratified data set. In both data sets, HEIWA model logits showed stronger correlations with the RMSF benchmark, with no evidence of reduced classification performance on the validation and test sets relative to the SUPFAM-stratified data set. However, the two data sets differed in several respects. Without HEIWA, the trade-off between biophysical soundness and model performance was more apparent in the SUPFAM-grouped data set. However, after HEIWA was added, the SUPFAM-stratified data set showed a clearer trend where all selected configurations with two solvent heads misidentified glycine as stabilizing and salt bridges and H-bonds as destabilizing. Increasing the number of solvent heads to 3 restored the stabilization effect of the salt bridges. Resolving the glycine and H-bond artifacts required a further increase to 4 solvent heads (Figure 3a). This trend is less apparent with the SUPFAM-grouped data set (Figure 7a). Also, all optimal hyperparameters obtained from the SUPFAM-stratified data set had more than twice as many parameters. This suggests that the diversity of the SUPFAM in the data set represents a limiting factor, which may give the stratified data set an advantage with no clear evidence of substantial data leakage.

Overall, HEIWA models trained on both data sets identify similar relationships between biophysical attributes and model logits, suggesting that the model did not meaningfully learn SUPFAM-specific summation because of the pooling operation; therefore, our design enables the model to learn individual residues’ contributions to protein thermophilicity and generalize this learning to SUPFAMs absent from the training data set. One indication of this is that model specificity was consistently higher than sensitivity, especially after adding the HEIWA module (Tables 1 and 2). A residue’s high RMSF may have little effect on the overall protein stability when the residue is located in a disordered region, which may systematically bias the model toward negative predictions.

### Biophysical Error Without HEIWA Concentrated at Surface Hydrophilic and Buried Hydrophobic Residues

Next, we ask which residues contribute most to biophysical error, as measured by discrepancies from RMSF values obtained from molecular dynamics simulations. VMD visualizations of these residues show very similar effects of HEIWA models trained on the SUPFAM-stratified (Figure S6a) and SUPFAM-grouped data sets (Figure S6b). Without HEIWA, the systematic underestimation of stability in most proteins is concentrated on buried hydrophobic residues and exposed charged residues. This is largely alleviated by the introduction of HEIWA. This is consistent with the tendency of proteins to position hydrophilic residues at the surface, where they interact with solvent.

Interestingly, a minority of proteins were stabilized under ESM3’s vacuum bias. The example shown in Figure S6 is *Shigella flexneri* DNA base-flipping protein (Accession: P0AFP3; SUPFAM: SSF46767). The effect’s restriction to surface-charged residues is slightly more apparent in the SUPFAM-stratified data set (Figure S6a). Although a thorough examination of the root cause of this phenomenon is beyond the scope of this study, we suspect that protein unfolding may be kinetically unfavorable under vacuum bias for certain structure types. As a result, the mobilizing effect of solvent–protein interactions may become dominant: the solvent, which is the most mobile component in the system, increases the RMSF of surface residues through bombardment and H-bond-mediated comovement.^50,51^ This interpretation is consistent with the observed RMSF-SASA and per-residue logit-SASA correlations (Figures 3c and 7c). This observation may help explain the unconventional secretion of certain aqueous proteins across membranes without a signal or transit peptide.^54,55^ Since many DNA-binding proteins are amphiphilic, they may exhibit this quality. Recently, the intramembrane complex TraDE was reported to bind DNA and mediate horizontal gene transfer.^56^

### Molecular Simulations Show Greater Kinetic Stability of *de novo* Structures Generated Using HEIWA

Without fine-tuning ESM3, we incorporated HEIWA into the ESM3 generation pipeline and assessed the fidelity of *de novo* proteins. Residue conservation analysis (Figure S7) shows that HEIWA does not disrupt residue conservation patterns. After reducing the embeddings to 2D space using UMAP, we observed no difference between ESM3 embeddings before and after HEIWA modification, suggesting that the HEIWA modification did not substantially alter the overall structure of the ESM3 embedding. The ESM3 embedding used for structure token unmasking showed segregation according to helix propensity, sheet propensity, and exposure level (Figure 8).

**Figure 8:**
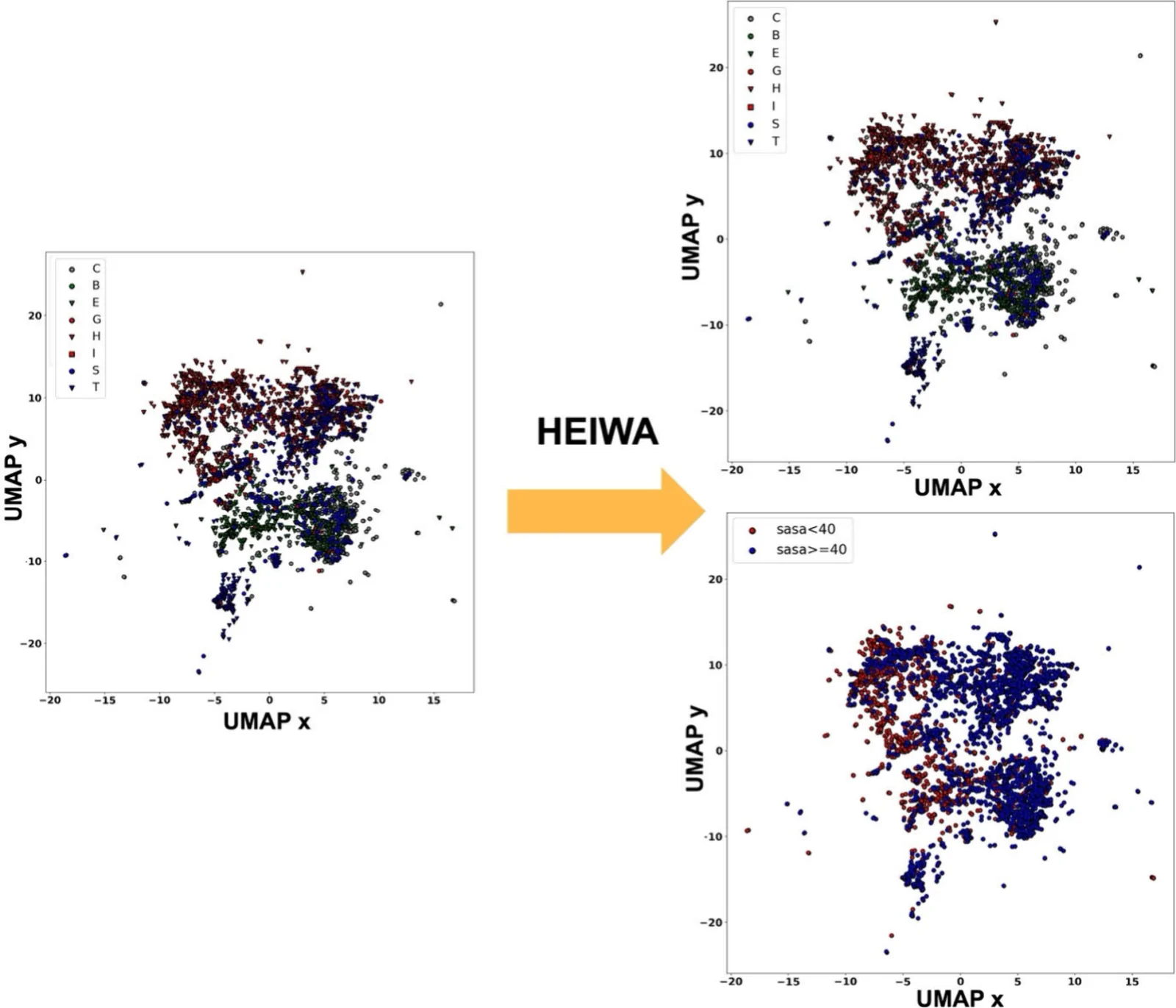
2D UMAP projection comparing the original ESM3 embeddings with the HEIWA-modified embeddings. The data structure of the embeddings was largely preserved, indicating that HEIWA acts primarily as a fine-tuner of the existing embedding. The ESM3 embedding used for structure decoding showed segregation according to helix propensity (red), sheet propensity (green), and residue-level surface area.

The problem of vacuum bias is more pronounced in proteins shorter than 512 amino acids due to their higher surface area-to-volume ratio, while the concerned physical errors are surface area-dependent. Therefore, we selected 19 mesophilic monomeric nonmembrane proteins, each from a unique SUPFAM, all shorter than 256 AA and distinct from the 70 proteins used for the biophysical benchmark, for *de novo* protein generation with ESM3, using two random seeds, H4 and M2 (Figure 1d). H4 is the random seed used for the third epoch of HEIWA model training, while M2 is an independent seed. The kinetic stability under thermophilic conditions of each mutant was assessed by molecular dynamics simulations using a temperature ramping scheme from 310 to 368 K over 100 ns (Table 3; Figures S8–S26).

**Table 3:**
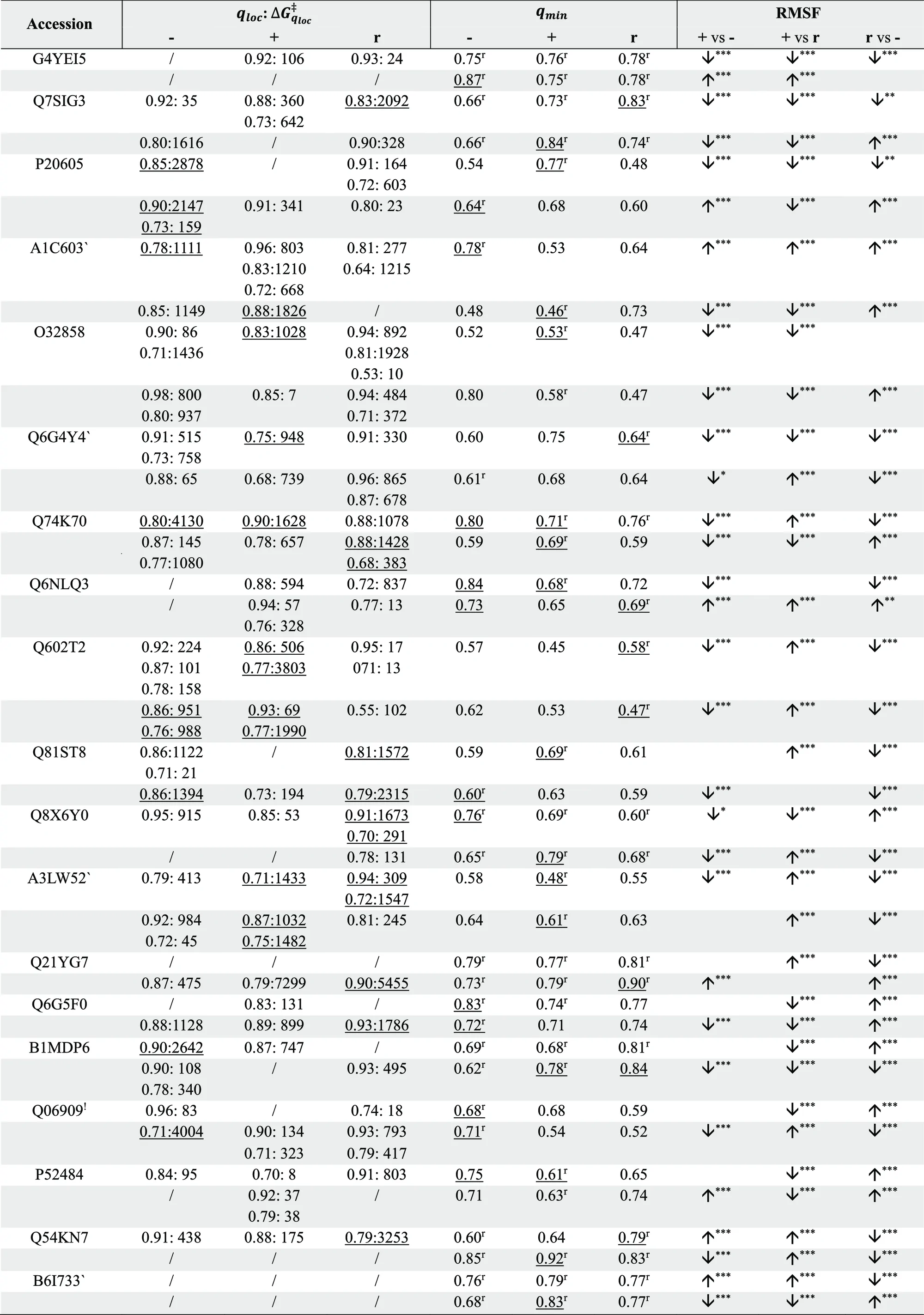
Summary of Molecular Dynamics Results, Including Metadynamics Simulations at 310 K and Temperature Ramping Experiments^a^

a “ˈ” indicates the protein is slightly disordered or has low pLDDT. “^!^” indicates the protein has a very large conformational change during the relaxation process (Table S2). Results obtained using the ESM3 random seed M2 are shaded in gray. The superscript “^r^” indicates that the RMSD of relaxation is either <2 Å or is the lowest among experimental treatments using the same random seed. Columns labeled with + , −, and r represent the three experimental treatments, including the presence of full HEIWA, ESM3 baseline, and HEIWA with reversed SASA gate, respectively. ***q*** denotes the percentage of native contacts defined after relaxation. ***q*_loc_** and Δ*G*_***q*_loc_**_^‡^ indicate the local minimum energy level and the associated energy barrier height (J/mol) against unfolding. ***q*_min_** means the percentage of native contacts in the global minimum. Considering the relaxation RMSD, the local and global minimum, the *de novo* protein with more stable free-energy profile is underlined. Columns under “RMSF” compare the RMSF among the ESM3 baseline, a full HEIWA, and HEIWA with reversed SASA gate during temperature ramping. For statistical significance: *p < 0.05, **p < 0.01, ****p < 0.001.

Paired Wilcoxon tests of RMSF showed that HEIWA can help ESM3 generate more kinetically stable structures in 23 out of 38 tested proteins (Figures S8–S18). The kinetic stability of 7 proteins was similar to with and without HEIWA (Figures S19–S24), while 8 proteins showed reduced stability after HEIWA was added (Figures S25 and S26). After ties were excluded, the difference was statistically significant according to an unsigned binomial test (Table 3; *p* = 0.011). In contrast, HEIWA with reversed SASA gate produced only 20 and 16 proteins, respectively, that were kinetically more and less stable than those generated by ESM3 (Table 3); therefore, the difference was not statistically significant using the same unsigned binomial test (Table 3; *p* > 0.05), whereas full HEIWA produced 19 proteins and 16 proteins, respectively, that are kinetically more and less stable than those generated with the reversed SASA gate; therefore, the difference was not statistically significant using the same binomial test (Table 3; *p* > 0.05). The reversed SASA gate was associated with extremely high RMSF in 7 proteins (Figures S9e, S10e, S21d, S21e, S22d, S23d, and S24d). Overall, only full HEIWA produced proteins with greater kinetic stability at high temperature.

We note that the impact of HEIWA on the RMSF of disordered regions was inconsistent across random seeds and protein structures, as HEIWA increased the mobility of low-pLDDT regions in multiple cases (Figures S8e, S13d, S14d, S15e, S17d, S19e, S21d, and S24e). Conversely, HEIWA also increased the stability of disordered regions in several cases (Figures S8d, S12d, S13e, S14e, S15d, S16d, S16e, S18d, S18e, S19d, S21e, and S24d). We believe that examining the impact of solvent encoding on disordered regions of ESM3-generated proteins will likely require a greater statistical power.

Among all HEIWA-stabilized proteins, system-wide kinetic and potential energies showed no consistent pattern, as HEIWA reduced the potential energy in 23 of 38 proteins and increased it in the remaining proteins. This suggests that steric clashes within the protein chain are not the principal cause of the increased stability observed in the HEIWA-generated proteins.

We next examined whether the metadynamics profiles at 310 K provided a consistent view of the free-energy profile. 17 of 38 wild-type proteins produced the lowest RMSD after relaxation, including prerun, using a full HEIWA (Table S2). One seeded 100 ns metadynamics trajectory was run for each system, and the resulting profiles were compared using the fraction of native contacts, ***q***, as the collective variable (Table 3; Figures S8–S26). In some cases, these profiles agreed with the temperature-ramping results, whereas in others they did not. With the H4 seed, G4YEI5 showed the greatest kinetic stability at high temperature when generated using full HEIWA, but metadynamics profile was similar to those of the ESM3 baseline and HEIWA with the reversed SASA gate (Table 3; Figure S8). With the M2 seed, Q8X6Y0 had the most stable free-energy profile with a full HEIWA due to its ***q*_min_** value of 0.79, but its RMSF at high temperature was higher than that of the structure generated with the reversed SASA gate (Figure S18). With the H4 seed, B6I733, a low-pLDDT protein, had a free-energy landscape with ***q*_min_** values comparable to the ESM3 baseline and the reversed SASA gate, but it showed the lowest kinetic stability at high temperature when generated using full HEIWA (Figure S26). However, the two analyses were consistent in some cases. With the M2 random seed and full HEIWA, Q7SIG3 produced a *de novo* structure that showed the best kinetic stability at high temperature and the highest ***q*_min_**. Using the M2 random seed and full HEIWA, A1C603 produced a *de novo* structure that exhibits the best kinetic stability at high-temperature conditions and a highly stabilizing local minimum at ***q*_loc_** = **0.88**. A similar agreement between the two analyses was observed for O32858 with the H4 seed and full HEIWA (Table 3).

Such differences are not unexpected because the analyses probe distinct aspects of protein behavior: temperature ramping simulations assess RMSF under a specified heating protocol, while metadynamics samples conformations under a bias applied along ***q***. This is because ***q*** does not uniquely define a protein conformation: structurally distinct states can have the same fraction of native contacts, and transitions may depend on coordinates not represented by ***q***. Because of this degeneracy, we interpret the metadynamics profiles as complementary qualitative context and do not use them to infer quantitative free-energy differences or as a definitive thermodynamic ranking of the native structure.

Taken together, the temperature ramping simulations support lower RMSF under the full-HEIWA condition than under the ESM3 baseline based on the tested protocol, while the comparisons involving the reversed-SASA-gate condition were not statistically significant. The metadynamics profiles provide qualitative context that is sometimes concordant but do not establish a thermodynamic stability ranking.

### Significance and Perspectives

When building machine learning systems for physical sciences, training a machine-learning model on a data set obtained from first-principles simulations often requires an intractable amount of computational resources. This challenge is particularly notable in quantum mechanics^57^ and drug design, where binding energy prediction using free energy perturbation is difficult to scale.^58^ To this end, machine learning’s ability to learn latent causal relationships has been exploited to develop surrogate model that reduce the computational requirements for first-principles simulations.^59,60^ In this study, rather than iteratively training a surrogate machine-learning model, we used ESM3’s SE(3)-embedding with a surrogate backpropagation objective: whole-protein thermophilic protein classification. Through model architecture-induced residue-level decomposition and biophysics-guided model selection, we identified models with optimized hyperparameters that predicted the contribution of individual residues to protein thermophilicity. As a result, rather than obtaining RMSF ground truth for every residue in the full data set, which contains 5125 (SUPFAM-stratified) and 5216 (SUPFAM-grouped) sequences, we only used a small molecular dynamics data set of 70 proteins as a physically grounded evaluation benchmark, representing 1.3–1.4% of the original data size.

This study demonstrates the possibility that a structure-based generative protein model can account for solvent interactions without requiring all-atom representations or molecular dynamics ground truth. Using thermophilic protein classification as a proxy task to assess force-field awareness, we show that solvent encoding can help remediate some of the systematic biophysical artifacts in ESM3, particularly the misidentification of H-bonds. We hypothesize that similar issues may have occur in other structure-based architectures. These results establish that solvent encoding is both feasible and beneficial at the residue level, and that the water–protein interface is a tractable, previously overlooked target for improving the physical grounding of protein language models. While Sumida et al. show that a structure-based generative model can generate stable *de novo* structures,^61^ this study provides complementary evidence about the behavior of generated structures and shows that solvent encoding can improve the kinetic stability of generated protein structures.

However, this study reveals that weak interactions such as π-π stacking, mediated by nonlocal electron correlations, may be masked by solvent encoding, possibly because H-bonds and salt bridges have stronger effects on protein stability (Figures 3a and 7a). The stabilizing effect of π-π interaction is also less likely to be identified in baseline models (Figures 2a and 6a). Given the low frequency of aromatic residues, it remains unclear if this is caused by an inherent lack of force-field information about π-π stacking or the small data set of this study. Nevertheless, ensuring that models can represent both strong and weak interactions will be crucial for building a protein model with holistic physical awareness, especially in disordered regions.^62^

Recent protein models have moved toward all-atom representations that allow biophysical interactions to be incorporated as structural prompts,^15,42–44^ offering a potential solution to this problem. However, many of these models do not fully account for solvent interactions, a limitation also noted by the authors of Boltz-2,^42^ AlphaFold3,^15^ and RFdiffusion3.^43^ Although solvent, ions, and ligands can be included as part of structural prompts based on the equilibrium states represented in the PDB database, it is questionable whether these prompts necessarily encode the causal solvent–protein codynamics over time. A close attempt at this was in BioEmu, a multimodule conformation generation pipeline that combines multiple sequence alignment and evoformer architecture for protein encoding, followed by a diffusion-based generative module. Extensive molecular dynamics were used to guide sampling from a realistic conformation sampling distribution without explicitly learning the protein’s fine-grained time-series evolution.^23^ This study demonstrates that adding solvent encoding to a residue-resolution model, such as ESM3, using attention is feasible and can improve its biophysical consistency, without acquiring all-atom representations.

Nevertheless, we argue that combining all-atom representations and solvent encoding could advance efforts to understand causality in protein physics — in particular, correcting physically implausible protein structures arising from prediction errors by modeling how water rearrangement precedes and drives changes in backbone conformation. Unlike molecular dynamics simulations, which integrate atomic forces at every time step, a transformer-based approach could learn a compressed representation of the solvent-mediated energy landscape, enabling direct sampling of conformationally relevant states at a fraction of the computational cost. Such temporal modeling could inspiration from video trajectory analysis and time-series decision-making in robotics, where sequence-to-sequence causality has been a central theme.^63,64^ Realizing these goals would require extending the present work, which used a precleaned data set that did not account for pH variation, nonaqueous cosolvents, protein–protein binding, or protein–ligand binding in an ESM3 setting. In particular, chaperone-protein interactions are critical, ATP-driven processes that ensure proper protein folding.^65^ Developing a sound biophysical understanding of protein models would be crucial to modeling these processes in the future. A paradigm that explicitly considers the global protein structure and respects the prompted, coordinate-agnostic catalytic structure more closely resembles the design of RFdiffusion3.^43^

## Conclusion

We demonstrated that the coordinate-informed ESM3 embedding can be used for thermophilic protein classification with high accuracy, but systematic biophysical misidentification exists as solvent encoding—a critical element for accurate physical understanding—is absent. We presented HEIWA, a hybrid explicit-implicit water attention mechanism that injects solvent-mediated force-field information into the ESM3 protein language model. Our design enables the model to learn the contributions of individual residues to protein thermophilicity. While HEIWA preserves the underlying data structure of the ESM3 embedding, it largely improves biophysical consistency and alleviates SASA-dependent errors. Molecular dynamics simulations of ESM3-generated proteins demonstrated HEIWA’s potential to improve kinetic stability under thermophilic conditions. Overall, this study demonstrates that solvent encoding is both feasible and beneficial for residue-resolution, structure-based protein language models.

## Materials and Methods

### Hardware

All model training and molecular dynamics simulations were performed on 4 desktop computers equipped with two 5090s, one 5080, and one 3090ti; each equipped with a 24-core Intel Ultra 9 285 K CPU.

### Data Set Preparation

Thermophilic protein data sets were compiled from the UniProtKB database as detailed in Table S1. In brief, all acidophilic, alkaliphilic, piezophilic, and halophilic proteins were excluded. Since protein stability is sensitive to single-point mutations, we ensured that all positive and negative samples had similar sequences. Only SUPFAMs containing both mesophilic and thermophilic proteins were retained. The data were split into train, validation, and test sets at a ratio of 0.6:0.2:0.2 using two methods: 1. SUPFAM-stratified splitting ensured that each partition contained samples from every SUPFAM. To this end, SUPFAMs with <5 unique sequences were excluded. 2. SUPFAM-grouped splitting ensured that all samples from the same SUPFAM were assigned to only one of the training, validation, or test sets.

### Model Training

All training runs were conducted using the Adam optimizer^66^ and epoch-specific random seeds as specified in our repository, with a learning rate decayed by 0.95 per epoch. Hyperparameter tuning began with 7 epochs for each combination in the first cycle, followed by one 3-epoch cycle, two 4-epoch cycles, two 5-epoch cycles, and so on. Only the top-performing 60% of the combinations were retained after each cycle. If a combination met the early stopping criteria, the best-performing filtered combination could be resumed from its last epoch. The early stopping criteria and dropout tuning were based on the minimum value of sensitivity, precision, and specificity in the training and validation metrics. The overfitting threshold was set to 4%, and the early stopping tolerance was set to 8 epochs.

The learning rate for training the base thermophilic classifier was 5 × 10^–5^ with an initial dropout of 0.2. In the subsequent training of the HEIWA model, the pretrained SASA gate, membrane gate, and base model were initialized at their pretrained state. The pretrained gates remained frozen throughout the training, whereas the base model parameters began updating in the second epoch. The learning rate was set to 1 × 10^–5^ to control overfitting. Dropout rates ranging from 0.1 to 0.5 were used in the HEIWA module. A high dropout rate was applied to the downstream SwiGLU layer. Additionally, we found that introducing loss clipping, which sets the loss to zero when the absolute prediction error is below 0.1, can help control overfitting.^67^

### ESM3 Protein Generation

We installed ESM3 version 3.2.0 and obtained model weights from the official Huggingface interface. Protein generation was performed by using temperature annealing. We first unmasked the structure tokens using 16 iterations and secondary structure tokens using 8 iterations, followed by SASA tokens using 4 iterations, then we repeated the structure token unmasking using 8 iterations, followed by the final sequence generation using 8 iterations. The unmasking temperature was set at 0.7. The HEIWA modification was applied immediately before the final sequence generation. Full-atom structures were subsequently generated using python Modeller 10.8.

### Biophysical Analysis

To identify networks of H-bonds, salt bridges, and π-π stacking within each protein, protein structure files were generated using VMD with the CHARMM force field.^68^ H-bond and salt bridge identified using the Python MDAnalysis version 2.8.0. For H-bonds, the distance and angle cutoffs were 3.5 Å and 160°, respectively. For salt bridges, the distance cutoff and angle cutoffs were 4 Å and 90°, respectively. π–π stacking interactions were identified using a distance cutoff of 3.8 Å. DSSP version 3.1.4 was used to identify secondary structure and estimate solvent-accessible surface area (SASA).

### Molecular Dynamics and Metadynamics

Molecular dynamics simulations were performed by using Gromacs version 2026.0. To eliminate steric clashes after full-atom generation, each protein structure was relaxed using 10 iterations of an energy minimization NVT cycle. Subsequent NPT relaxation followed a sequential scheme, gradually reducing the force constant. In chronological order, the force constants were 100, 316, and 100 applied for 50 ps, followed by 40, 16, 7, 3, 1, 0.5, and 0.2. Finally, a 1 ns run was performed without constraints using the Berendsen barostat. The main production MD run was conducted under constant pressure using a Parrinello–Rahman barostat. The first 30 ns were discarded as prerun. The main production run used a time step of 2 fs. Each simulation, including the prerun, was performed in triplicate with replicate-specific random seeds. The TIP4P/2005 ^69^ water model and the amber99sbws force field^70^ were used. The system was solvated with 0.15 M NaCl in a dodecahedral box with a 1.2 nm margin. The protein run durations per replicate were 100 ns for temperature ramping and 20 ns for obtaining the 70-protein RMSF benchmark.

Metadynamics simulations were performed by using plumed version 2.10. All atom pairs separated by less than 0.4 nm were defined as native contacts (***Q***) if their residue indices differed by at least 4. Every 500 steps, a Gaussian hill with a standard deviation of 0.02 and a height of 0.5 kJ/mol was deposited. A bias factor of 10 was used to ensure well-tempered metadynamics at 310 K. To restrict sampling to the near-native and partially unfolded conformational regions, harmonic lower and upper walls were applied at ***Q*** = 0.50 and ***Q*** = 1.02, respectively, with a force constant of 3000 kJ/mol. The metadynamics bias was stored on a one-dimensional grid spanning −0.02 to 1.05, sectioned into 250 bins, and the deposited Gaussian hills were recorded for subsequent free energy reconstruction. A single 100 ns simulation was performed for 100 ns for each protein.

## Supplementary Material



## Data Availability

The code and the database are available on GitHub at https://github.com/Ernest-NH-Ho/heiwa4h.

## Notes

Additional experimental methods and results, including details of the molecular dynamics data, training and validation (PDF)
