# Supplementary material for: HEIWA: Hybrid Explicit–Implicit Water Attention Affects Force Field Accuracy in Structure-Based Protein Language Model ESM3
Source: J Chem Inf Model. 2026 Oct 23;66(21):13827–45. doi: 10.1021/acs.jcim.6c01844 (PMC13753520; doi:10.1021/acs.jcim.6c01844)
Supplement: Supplementary file 1 [file ci6c01844_si_001.pdf]

Supporting Information

HEIWA: Hybrid Explicit–Implicit Water Attention Affects Force Field Accuracy in Structure-Based Protein Language Model ESM3

Ngai Hei Ernest Ho[1], Chao Tung Yang[2,3,4], I-Son Ng[1,*], Jo-Shu Chang[1,4,5,6*]

[1] Department of Chemical Engineering, National Cheng Kung University, Tainan 701, Taiwan

[2] Department of Computer Science, Tunghai University, Taichung 407, Taiwan

[3] Department of Medical Research, Kuang Tien General Hospital, Taichung 433, Taiwan

[4] Research Center for Smart Sustainable Circular Economy, Tunghai University, Taichung 407, Taiwan

[5] Department of Chemical and Material Engineering, Tunghai University, Taichung 407, Taiwan

[6] Department of Chemical Engineering and Materials Science, Yuan Ze University, Chung-Li 302, Taiwan

*Corresponding authors:

Prof. I-Son Ng (email: yswu@mail.ncku.edu.tw)

Prof. Jo-Shu Chang (email: changjs@mail.ncku.edu.tw)

The Supporting Information includes

Supporting Figures **S-3**

Supporting Tables **S-53**

References **S-56**

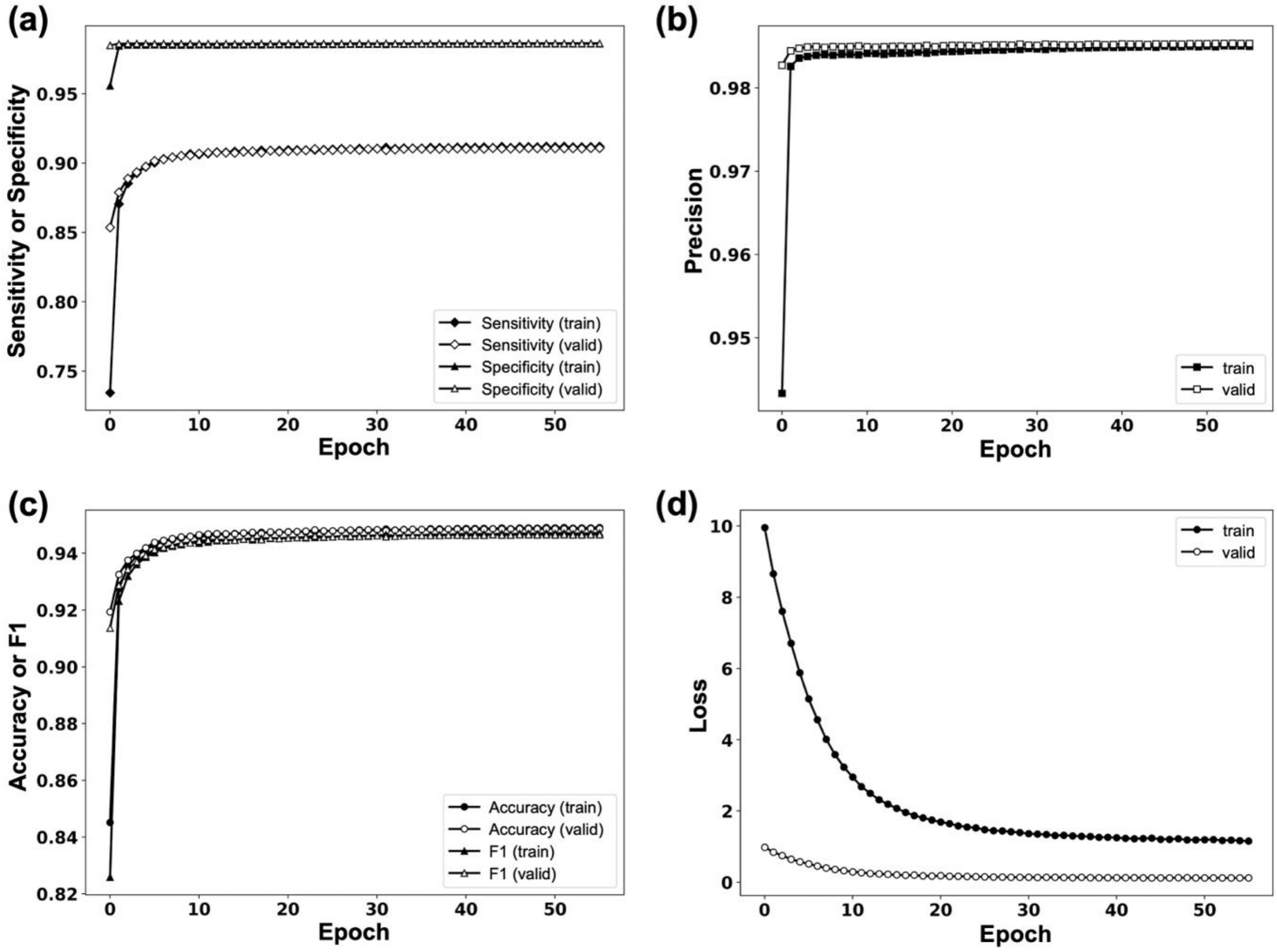

Figure S1. Training and validation learning curves for the SASA-based surface residue classifier. This gate masks solvent interactions with buried residues during HEIWA attention computation. The dataset was prepared from the UniProtKB database using DSSP, with the threshold set to 15.

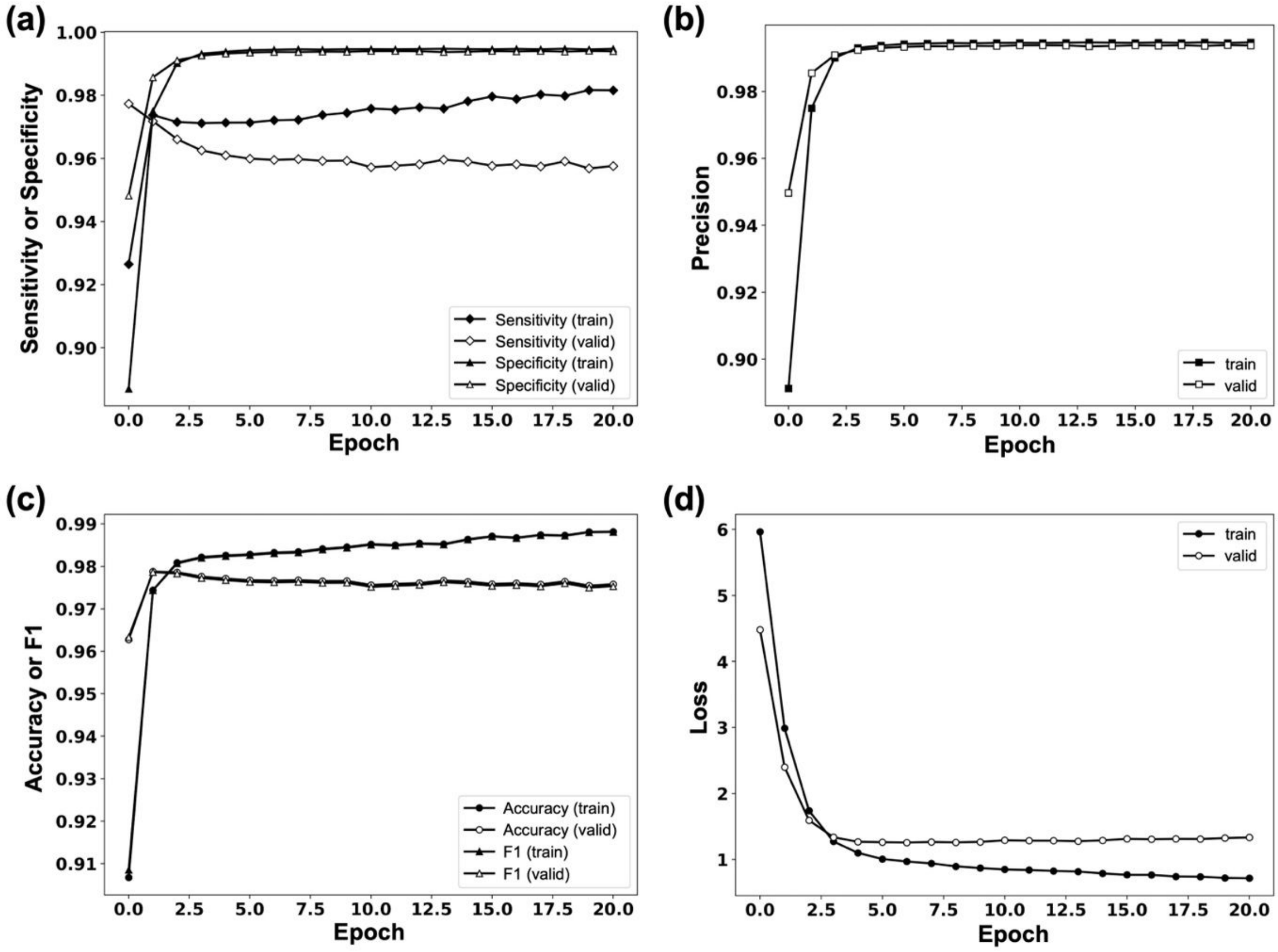

Figure S2. Training and validation learning curves for the membrane phase classifier. This gate masks solvent interactions with membrane-phase residues during HEIWA attention computation. The dataset was prepared from the UniProtKB database.

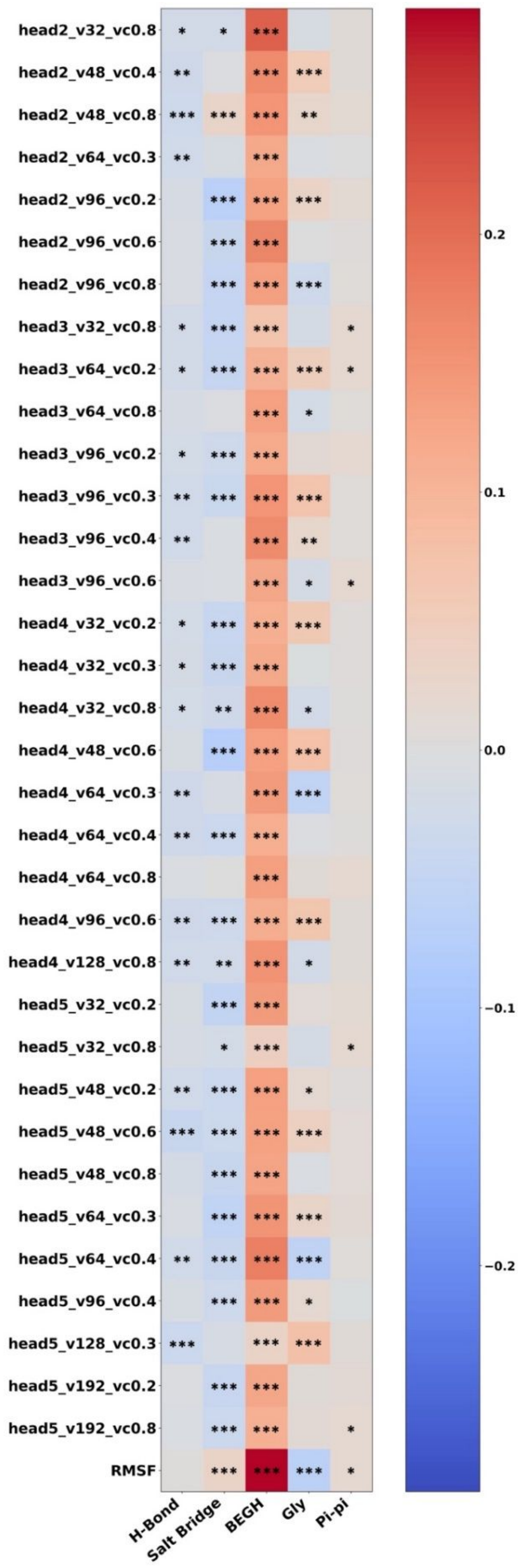

Figure S3. Biophysical performance of the 48-dim thermophilic classifier with the HEIWA module. Spearman correlations between per-residue logits and biophysical attributes (RMSF, H-bond count, SASA, hydrophobicity) across different hyperparameter configurations. Value-vector dimensions are indicated by "v", and SoRA compression factors by "vc". Red indicates

a correlation that suggests a positive contribution to protein thermophilicity; blue indicates a correlation that suggests a negative contribution.

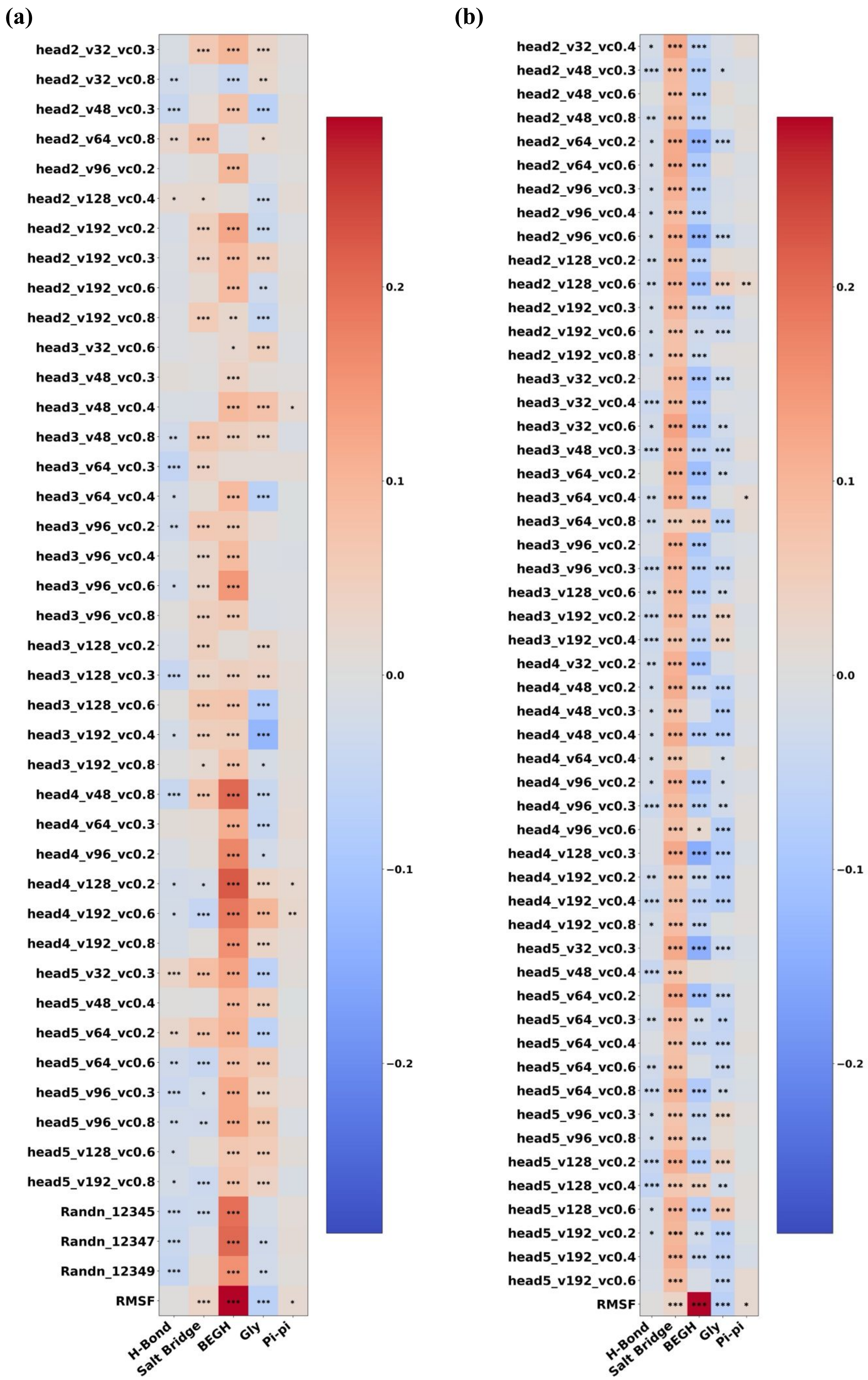

(a)
head2_v32_vc0.3
head2_v32_vc0.8
head2_v48_vc0.3
head2_v64_vc0.8
head2_v96_vc0.2
head2_v128_vc0.4
head2_v192_vc0.2
head2_v192_vc0.3
head2_v192_vc0.6
head2_v192_vc0.8
head3_v32_vc0.6
head3_v48_vc0.3
head3_v48_vc0.4
head3_v48_vc0.8
head3_v64_vc0.3
head3_v64_vc0.4
head3_v96_vc0.2
head3_v96_vc0.4
head3_v96_vc0.6
head3_v96_vc0.8
head3_v128_vc0.2
head3_v128_vc0.3
head3_v128_vc0.6
head3_v192_vc0.4
head3_v192_vc0.8
head4_v48_vc0.8
head4_v64_vc0.3
head4_v96_vc0.2
head4_v128_vc0.2
head4_v192_vc0.6
head4_v192_vc0.8
head5_v32_vc0.3
head5_v48_vc0.4
head5_v64_vc0.2
head5_v64_vc0.6
head5_v96_vc0.3
head5_v96_vc0.8
head5_v128_vc0.6
head5_v192_vc0.8
Randn_12345
Randn_12347
Randn_12349
RMSF
H-Bond
Salt Bridge
BEGH
Gly
Pi-pi
0.2
0.1
0.0
−0.1
−0.2
(b)
head2_v32_vc0.4
head2_v48_vc0.3
head2_v48_vc0.6
head2_v48_vc0.8
head2_v64_vc0.2
head2_v64_vc0.6
head2_v96_vc0.3
head2_v96_vc0.4
head2_v96_vc0.6
head2_v128_vc0.2
head2_v128_vc0.6
head2_v192_vc0.3
head2_v192_vc0.6
head2_v192_vc0.8
head3_v32_vc0.2
head3_v32_vc0.4
head3_v32_vc0.6
head3_v48_vc0.3
head3_v64_vc0.2
head3_v64_vc0.4
head3_v64_vc0.8
head3_v96_vc0.2
head3_v96_vc0.3
head3_v128_vc0.6
head3_v192_vc0.2
head3_v192_vc0.4
head4_v32_vc0.2
head4_v48_vc0.2
head4_v48_vc0.3
head4_v48_vc0.4
head4_v64_vc0.4
head4_v96_vc0.2
head4_v96_vc0.3
head4_v96_vc0.6
head4_v128_vc0.3
head4_v192_vc0.2
head4_v192_vc0.4
head4_v192_vc0.8
head5_v32_vc0.3
head5_v48_vc0.4
head5_v64_vc0.2
head5_v64_vc0.3
head5_v64_vc0.4
head5_v64_vc0.6
head5_v64_vc0.8
head5_v96_vc0.3
head5_v96_vc0.8
head5_v128_vc0.2
head5_v128_vc0.4
head5_v128_vc0.6
head5_v192_vc0.2
head5_v192_vc0.4
head5_v192_vc0.6
RMSF
H-Bond
Salt Bridge
BEGH
Gly
Pi-pi
0.2
0.1
0.0
−0.1
−0.2

(c)

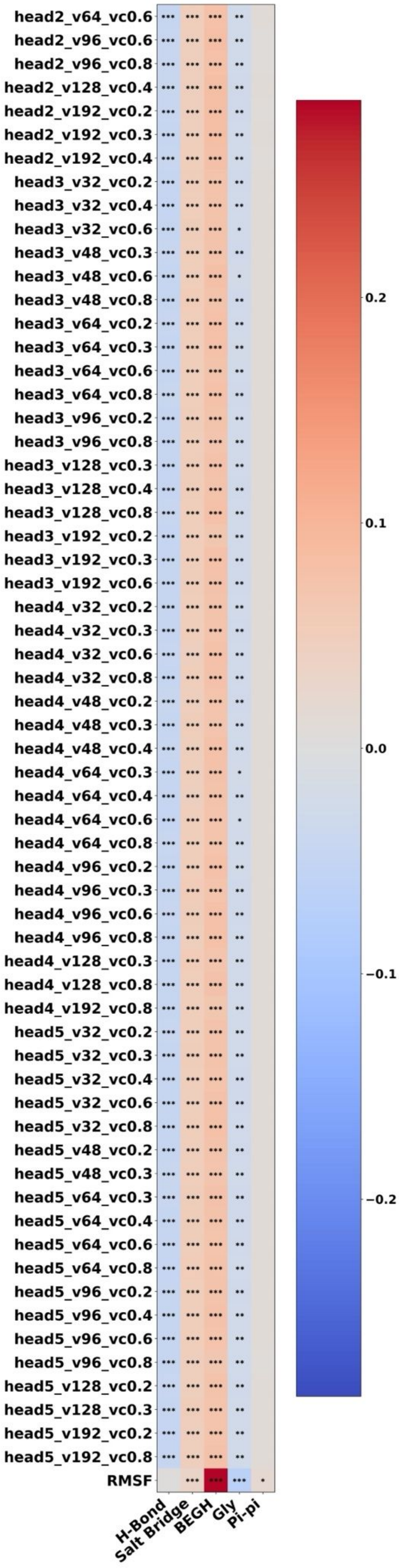
head2_v64_vc0.6
head2_v96_vc0.6
head2_v96_vc0.8
head2_v128_vc0.4
head2_v192_vc0.2
head2_v192_vc0.3
head2_v192_vc0.4
head3_v32_vc0.2
head3_v32_vc0.4
head3_v32_vc0.6
head3_v48_vc0.3
head3_v48_vc0.6
head3_v48_vc0.8
head3_v64_vc0.2
head3_v64_vc0.3
head3_v64_vc0.6
head3_v64_vc0.8
head3_v96_vc0.2
head3_v96_vc0.8
head3_v128_vc0.3
head3_v128_vc0.4
head3_v128_vc0.8
head3_v192_vc0.2
head3_v192_vc0.3
head3_v192_vc0.6
head4_v32_vc0.2
head4_v32_vc0.3
head4_v32_vc0.6
head4_v32_vc0.8
head4_v48_vc0.2
head4_v48_vc0.3
head4_v48_vc0.4
head4_v64_vc0.3
head4_v64_vc0.4
head4_v64_vc0.6
head4_v64_vc0.8
head4_v96_vc0.2
head4_v96_vc0.3
head4_v96_vc0.6
head4_v96_vc0.8
head4_v128_vc0.3
head4_v128_vc0.8
head4_v192_vc0.8
head5_v32_vc0.2
head5_v32_vc0.3
head5_v32_vc0.4
head5_v32_vc0.6
head5_v32_vc0.8
head5_v48_vc0.2
head5_v48_vc0.3
head5_v64_vc0.3
head5_v64_vc0.4
head5_v64_vc0.6
head5_v64_vc0.8
head5_v96_vc0.2
head5_v96_vc0.4
head5_v96_vc0.6
head5_v96_vc0.8
head5_v128_vc0.2
head5_v128_vc0.3
head5_v192_vc0.2
head5_v192_vc0.8
RMSF
H-Bond
Salt Bridge
BEGH
Gly
Pi-pi
0.2
0.1
0.0
−0.1
−0.2

Figure S4. Ablation study of both the SASA gate and membrane gate trained on the SUPFAM-stratified dataset. (a) Removal of both the SASA and membrane gates occasionally resulted in sound biophysical correlations. (b) Reversing the SASA gate resulted in only one hyperparameter configuration exhibiting sound biophysical understanding. (c) Reversing the membrane gate resulted in no hyperparameter configuration exhibiting sound biophysical understanding, in each case because H-bonds were identified as “destabilizing”.

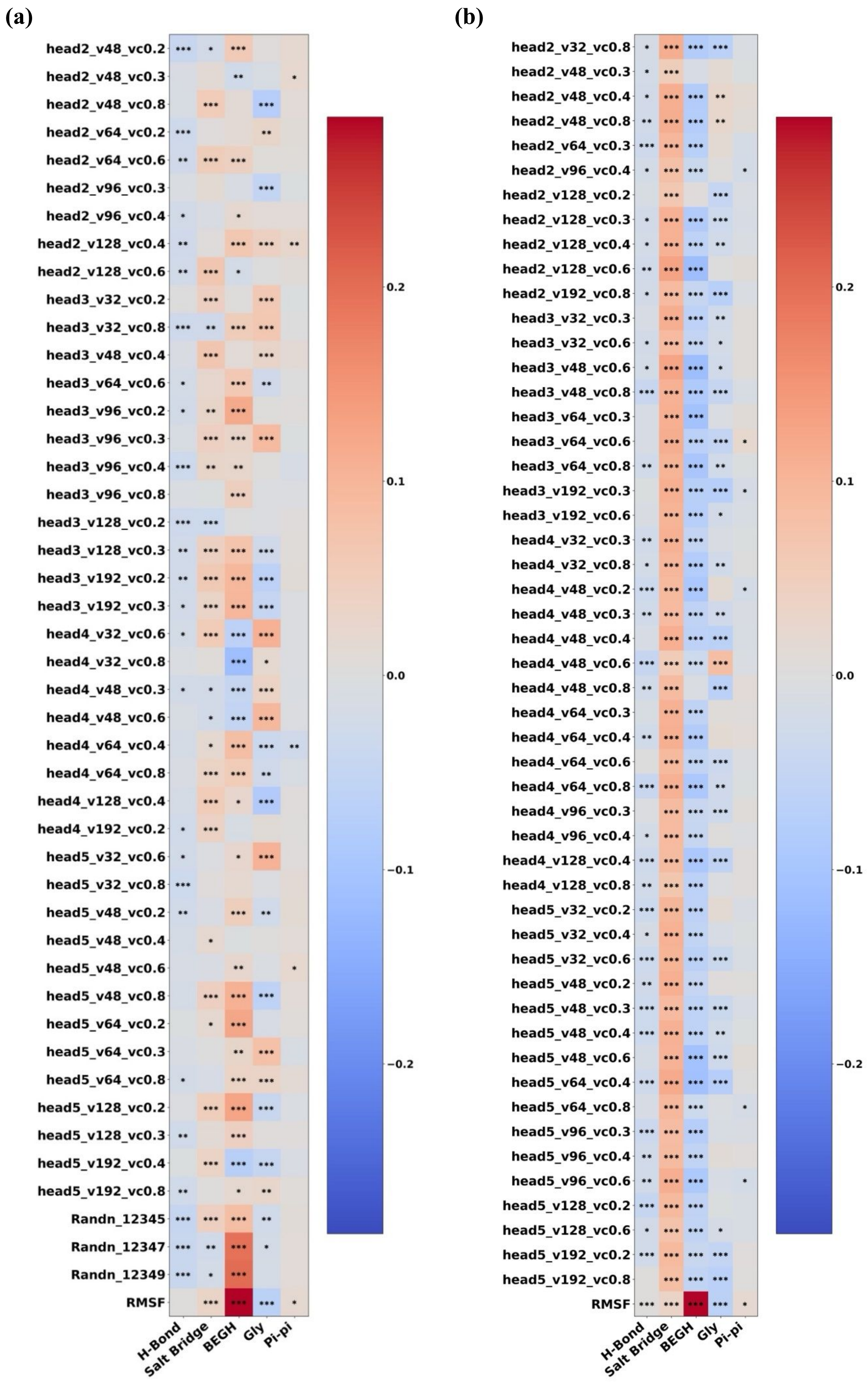
(a)
head2_v48_vc0.2
head2_v48_vc0.3
head2_v48_vc0.8
head2_v64_vc0.2
head2_v64_vc0.6
head2_v96_vc0.3
head2_v96_vc0.4
head2_v128_vc0.4
head2_v128_vc0.6
head3_v32_vc0.2
head3_v32_vc0.8
head3_v48_vc0.4
head3_v64_vc0.6
head3_v96_vc0.2
head3_v96_vc0.3
head3_v96_vc0.4
head3_v96_vc0.8
head3_v128_vc0.2
head3_v128_vc0.3
head3_v192_vc0.2
head3_v192_vc0.3
head4_v32_vc0.6
head4_v32_vc0.8
head4_v48_vc0.3
head4_v48_vc0.6
head4_v64_vc0.4
head4_v64_vc0.8
head4_v128_vc0.4
head4_v192_vc0.2
head5_v32_vc0.6
head5_v32_vc0.8
head5_v48_vc0.2
head5_v48_vc0.4
head5_v48_vc0.6
head5_v48_vc0.8
head5_v64_vc0.2
head5_v64_vc0.3
head5_v64_vc0.8
head5_v128_vc0.2
head5_v128_vc0.3
head5_v192_vc0.4
head5_v192_vc0.8
Randn_12345
Randn_12347
Randn_12349
RMSF
H-Bond
Salt Bridge
BEGH
Gly
Pi-pi
0.2
0.1
0.0
−0.1
−0.2
(b)
head2_v32_vc0.8
head2_v48_vc0.3
head2_v48_vc0.4
head2_v48_vc0.8
head2_v64_vc0.3
head2_v96_vc0.4
head2_v128_vc0.2
head2_v128_vc0.3
head2_v128_vc0.4
head2_v128_vc0.6
head2_v192_vc0.8
head3_v32_vc0.3
head3_v32_vc0.6
head3_v48_vc0.6
head3_v48_vc0.8
head3_v64_vc0.3
head3_v64_vc0.6
head3_v64_vc0.8
head3_v192_vc0.3
head3_v192_vc0.6
head4_v32_vc0.3
head4_v32_vc0.8
head4_v48_vc0.2
head4_v48_vc0.3
head4_v48_vc0.4
head4_v48_vc0.6
head4_v48_vc0.8
head4_v64_vc0.3
head4_v64_vc0.4
head4_v64_vc0.6
head4_v64_vc0.8
head4_v96_vc0.3
head4_v96_vc0.4
head4_v128_vc0.4
head4_v128_vc0.8
head5_v32_vc0.2
head5_v32_vc0.4
head5_v32_vc0.6
head5_v48_vc0.2
head5_v48_vc0.3
head5_v48_vc0.4
head5_v48_vc0.6
head5_v64_vc0.4
head5_v64_vc0.8
head5_v96_vc0.3
head5_v96_vc0.4
head5_v96_vc0.6
head5_v128_vc0.2
head5_v128_vc0.6
head5_v192_vc0.2
head5_v192_vc0.8
RMSF
H-Bond
Salt Bridge
BEGH
Gly
Pi-pi
0.2
0.1
0.0
−0.1
−0.2

(c)

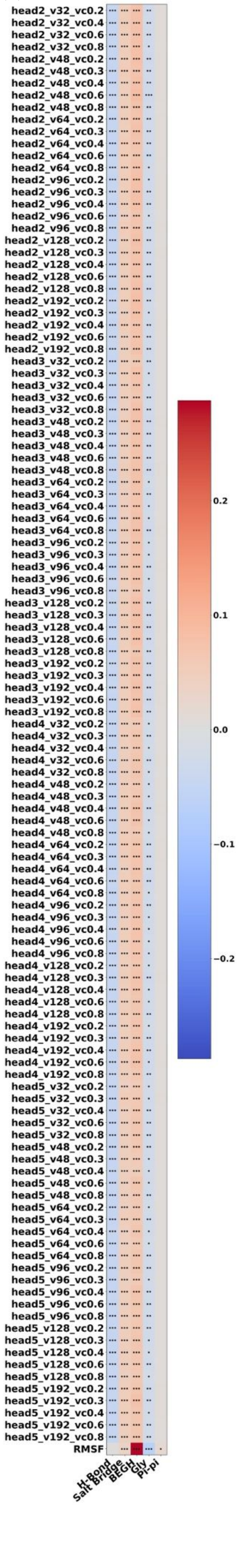

Figure S5. Ablation of both the SASA gate and membrane gate trained on the SUPFAM-grouped dataset. (a) Removal of both the SASA and membrane gates occasionally resulted in sound biophysical correlations. (b) Reversing the SASA gate resulted in no hyperparameter configuration exhibiting sound biophysical understanding. (c) Reversing the membrane gate resulted in no hyperparameter configuration exhibiting sound biophysical understanding, in each case because H-bonds were identified as “destabilizing”.

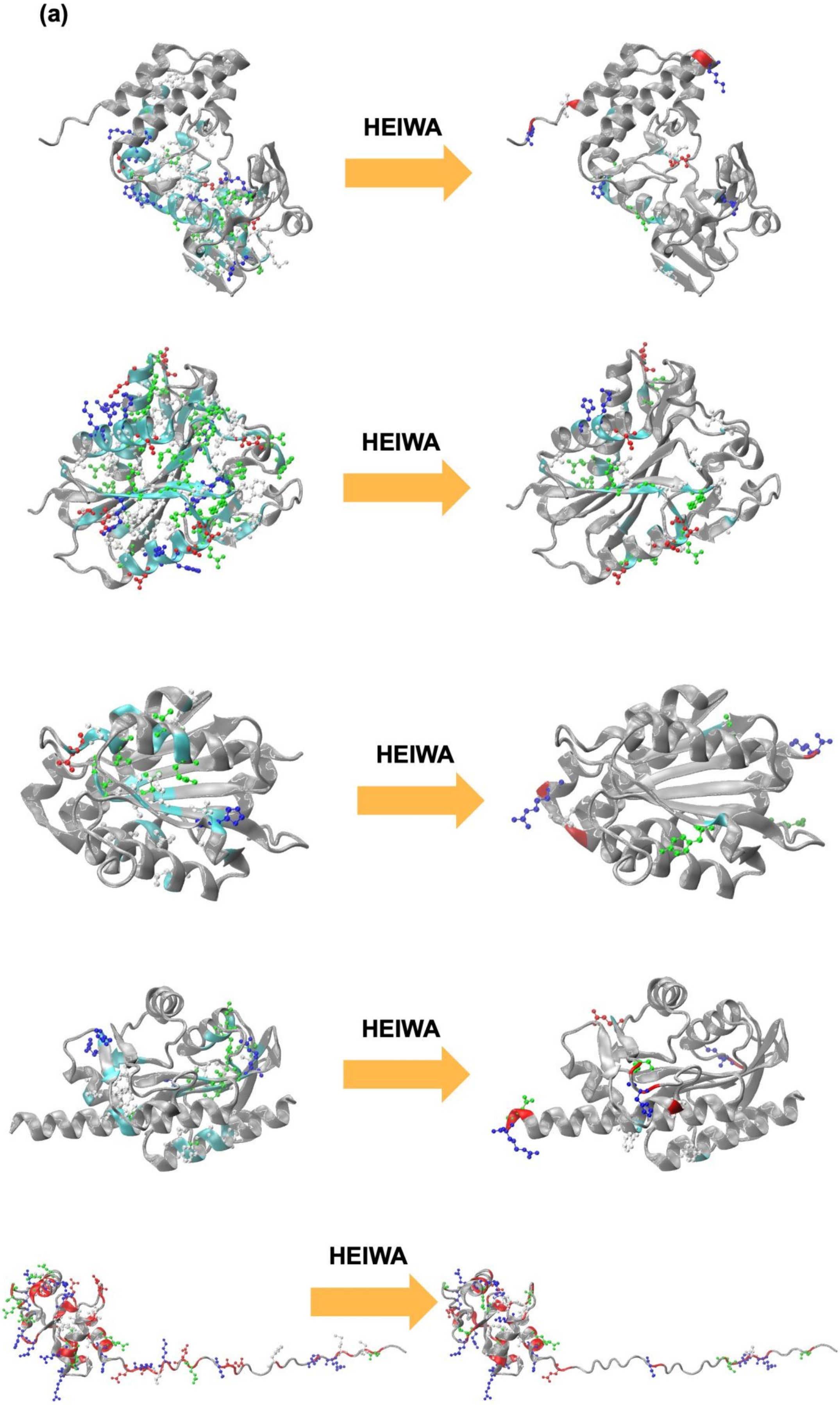
(a)
HEIWA
HEIWA
HEIWA
HEIWA
HEIWA

(b)

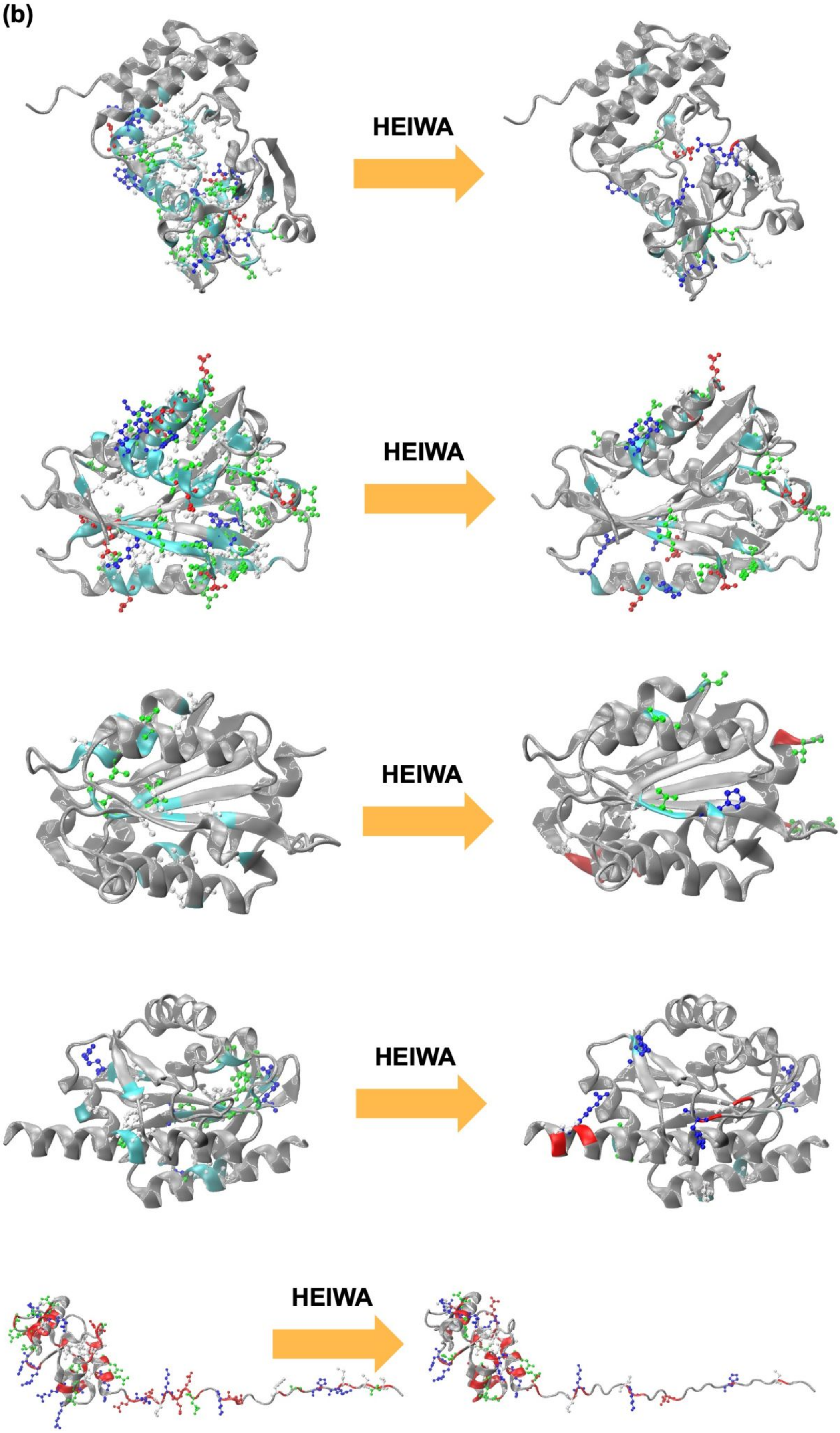

Figure S6. Comparison of RMSF and Spearman rank of model logit estimates. Severe misestimation of stability is defined by absolute differences in Spearman rank >60%. (a) Model "Head4_v192_vc0.8" is trained from the SUPFAM-stratified dataset. (b) Model "Head4_v64_vc0.6" was trained on the SUPFAM-grouped dataset. The proteins shown from the top are *Photobacterium damsela* 3-deoxy-D-manno-octulosonic acid kinase (Accession: P58551; SUPFAM: SSF56112), *Haemophilus influenzae* Octanoyltransferase (Accession: A5UBB1; SUPFAM: SSF55681), *Staphylococcus aureus* Molybdenum cofactor biosynthesis protein B (Accession: Q6G747; SUPFAM: SSF53218), *Pseudomonas savastanoi* Methylthioribulose-1-phosphate dehydratase (Accession: Q48KM8; SUPFAM: SSF53639), and *Shigella flexneri* DNA base-flipping protein (Accession: P0AFP3; SUPFAM: SSF46767). Backbone are shown in cyan when stability is severely underestimated and in red when it is severely overestimated. In these residues, all atoms are shown with positively charged residues in blue, negatively charged residues in red, hydrophilic residues in green, and hydrophobic residues in white.

```
>Entry=A1C603 SUPFAM=SSF82114; Sites=112,44,46

                 1                                                          60
WT               MRPDGPRDPVVGPDSGPEPPYPVRLSGPVIKGFGRGSKELGIPTANIPAEELAEHPDLQV
- (H4)           MLPPGPRPLVVGPASGLEPPYPILLSGPVIKGFGRGSKELGIPTANLPDEVLAEHPSLKS
- (M2)           MRPPGPRPLVVGPDSGPLPPFPIRLSGPVIKGFGRGSKELGIPTVNLPAEILADHPELKT
+ (H4)           MRTDGPPDPIVGPPSGLEPPYPIKLSGPVLKGFGRGSKELGIPTANLPAEVLDEHTDLKT
+ (M2)           MRPLGTRPLLVGSDSGPEPPYPIYLSGPVIRGFGRGSKELGIPTANLPGEILADHPFLKT
rSASA (H4)       MPPPGPRPLTVGPPSGPPPPYPIRLSGPILKGFGRGSKELGIPTVNLPAHILAKHTSLKS
rSASA (M2)       MRPPGPVPRVVGPPEGLEPPYPIELSGPVVKGFGRGSKELGIPTANLPAEVLAAHPQLKS

                 61                                                        120
WT               GVYYGVVALDPAKFQYHGDASRKGEDSQAAILPAVLSIGYNPFYKNKTRSIEIHIMPPLS
- (H4)           GVYFGWATLDGSKTVLDGDASRLGPDAKPLLYDMVMSVGTNPFYKNTTRSLEVHLFPPED
- (M2)           GIYFGWARFDPAKFEWLGDAAERGPDNRAPLREMVLSIGWNPFYKNETRSLEVHLLPPPL
+ (H4)           GVYFGFLSTDVSEYEYRGDPSKKGENLRPEIREIVMSIGFNPFYKNETRSLETHIHPSLS
+ (M2)           GIYFGWASIDPSKFKKEGDASYKAEDGEVSYHKMVMSVGWNPFYKNKTRSLEVHLIPEEV
rSASA (H4)       GIYYGFATLDPATTRPVGDASAAAADAEPRFLRMVMSIGWNPFYKNTTRSVEIHLLPPDD
rSASA (M2)       GIYFGWARTDVEEFKVEGRPDREGEDAEGEVFKMVMSVGFNPFYLNTVRSLEVHLLPPPL

                 121                                                       180
WT               SPSPTAEVTTQGQGHGQVKFHKLPDFYGTQLKLLILGYIRPEFDYVSLEALVEDIRVDCE
- (H4)           SPLPPLEVDPADASLGKVKRVPLPDFYGAEIRLTVVGYLRPELDYTSLEALVADIRVDVE
- (M2)           ASSDVPEEDDEGEGEGEEERPELPDFYGAEIEMVVLGYLRDELDFESLEALVEAIREDVE
+ (H4)           SVSSDSKDNSDSKSDGKVKREKLPDFYGQKIYLTVLGYIRPELDFDSLEALIEAIRSDVE
+ (M2)           AVSDVSEDDSLGLGKAKLKKRKLPDFYGAEIYLQVLGFIRPELDYDSLEALIEDIRVDVE
rSASA (H4)       LPDGTAALDSDDGDAEAVVKPVLPDYYGAEIRLTILGYIRPELDFDSLEELIEDIHEDIE
rSASA (M2)       PPDEDDEDVDEAAAAGKVKERPYPDFYGARLRLRVLGYIRPELDYDSLEALVEDIKVDVE

                 181                                                       214
WT               VARASLQRPAYERYLAGGQGLDAVEKQRRWLVSF
- (H4)           TARASLERPEYKKLRDGGEGVSAEDEERRSLWVF
- (M2)           AARASLQRPYAQRWRAGGDGLDWEARRREEEESE
+ (H4)           AARASLERPAYQKLLSGGEGEDAEEHRKKLVVSV
+ (M2)           TAKASLKRPYHEKLLAGGEGWDDFEKERRRLEAK
rSASA (H4)       TARASLKRPYAARWLDGGKGLSEEERDKLWTAPV
rSASA (M2)       TARASLERPWAVKYEAWPEGEEAVVEERKKELEK
```

```
>Entry=A3LW52 SUPFAM=SSF52799; Sites=146

               1                                                          60
WT             MSNKDTSILKGNVDHEEADSNPKLRKIGRPPALRIIPPLNFCPVERQLYRSGQPSAINQS
- (H4)         MALKDNSHLLGVVPLEEVPATPVFEEIGEPPAKKLIPPKNFSVVEENVYRSGLPSAEHFP
- (M2)         MPVLVPPILKGNLNLEKALTLPLLKIKGIPPAKLLIPPENFSVVEKNIYRSGFPSSENFS
+ (H4)         MTEKNEPIIIGTSVKLEINPDPKIYEIGEVPAEKLIPPENFSVVEENIYRSGFPTAEHFP
+ (M2)         MAEKDPSHLLGTVTFEEAPSDPKIKPKGKIPEVKLIPPENFSQVEENLYRSGLPTPENFP
rSASA (H4)     MSNIDESIEKAIEELKKILSNPKFIIKGKPPSKKLIPPENFSQIEKNIYRSGYPTPEHFS
rSASA (M2)     MSLKNVANKIGEPIELKSETVPIKKIEGEVPSKKLVAPKNFSVVEENIYRSGLPSAENLS

               61                                                        120
WT             FLEQLNLKTILWLASEEPQDDFLEFSNDHNINIEFVGIINEFSNYQNYNTNQTLTVNPWD
- (H4)         FLEQLNLKSIVYLAAEKPEPGFIEFLNEHNINLIEIGMENLLDDYHLYVHNKSLSVDVET
- (M2)         FLELLNLKCIINLAPEKTKEELIKFLEENNIVLIEIVIYSPNNVYENYNTNIQPSKDPFV
+ (H4)         FLEKLNLKSILYLCEEEYEPSFIEFFEEHNINVHHVGFYTEKVLLKHFKSDEPVTVNPYD
+ (M2)         FLEQLNLKSILYLCEEEYPEEFQEFLKEHNIKVFRVNMYKNNLNKLNLNENKVITIDPYS
rSASA (H4)     FLEKLNLKTILRIDTEEPKDYLIEFLNENNINLEYIGIKHDKGTKLDKVTNESYTYDGYS
rSASA (M2)     FLNQLNLKSILYLCPEEYKPFIIKFLEENNINLIKVGMVNEYNIYELYNENKIKTGNPYE

               121                                                       180
WT             ALNEQTIKRALELIVNRENYPLLVCCGMGRHRTGTVIGCLRRLQGWNLASVSEEYRRFTG
- (H4)         AFPEEAMKKALEVLFDRRNYPLLIHCNQGKHRTGCVVACLRKLQGWPLSAVLTEYRLFCG
- (M2)         DLNKENIIKALQLIFNKDNYPLLIHCNKGKHRTGIIIACLRKLQNWPLSAVLEEYKRFCG
+ (H4)         GINEENIIKALQIIFDRRNHPLLIHCNKGKHRTGVVIACLRKLQGWPLSAVFEEYRRFCG
+ (M2)         SINEESIKKALNIIFNKDNYPMLIHCNKGKHRTGIIIACLRKLQKWPLPAIFEEYKRFCG
rSASA (H4)     GINEELIIKALQEILDKNNYPMLIHCNHGKHRTGIIVACLRKLQGWPLSAIIEEYKKFIG
rSASA (M2)     SLNEENIIKALQVIFNKDNYPMLIHCNHGKDRTGIVVAILRKLQNWPLSAVLEEYKLFLG

               181                                                       212
WT             ARGGRILVELLIESFDIGSVEIDPSKVPDWLT
- (H4)         SKSGKIIDENFIESFDIGSVKVDPAYLPPWLL
- (M2)         SKFGRKEDIQFIESFDIGSVELDPSKLPDSWF
+ (H4)         AKGAREKDLQFIETFDIGSVQLDPAKLPDWFK
+ (M2)         SKGTREEDLEFIENFDISSVEIDPSKVLPSFL
rSASA (H4)     SKGGKEIDIKFIENFNIGSVKIDISKAPDWLK
rSASA (M2)     SKGVRERDIEIIETFDIGSVEIDPSKAPPIFF
```

```
>Entry=B1MDP6 SUPFAM=SSF53448; Sites=145,160,163

             1                                                          60
WT           MGRQAGTDFGVIIAVKNLATAKSRLAPSLPAPQRERLVLGMLTHAIAVAFSAAGTVAVIS
- (H4)       MTERPGSDLSVIIAVKPLATAKTRLAPVLDAPRRERLVLAMLRRTIAVAQAAAARVAVVS
- (M2)       MSDDIATDVGVIIPVKDLATAKTRLAPVYPAAERLALVEAMLRHVIAVAKAAAGAVAVIS
+ (H4)       MGRRAGDDWSVIIPVKPLATAKSRLAPVFDAPRRERLVLAWVEHTIAVARAVAARVALVG
+ (M2)       MSERIGSDWGVLIPVKRLDTAKTRLAPAFPPPQRRRLVLAMLVRTIAVCQACAGLVAVIS
rSASA (H4)   MTTGTTPDWNVIIPVKDLATAKTRLAPVMPPAERLRLIEGMLRHVIAVAHAAASAVAVVS
rSASA (M2)   MGNMSASPWSVIIPVKPLATAKSRLAPVLPAPQRRRLVLALLVRTIAVCRAVCAEVAVIS

             61                                                        120
WT           PDPTAKAAASEAGARFIFDDTPEGHPDPLNNALRSAAAQLRASSPNLVVLQADLPVVTAT
- (H4)       RDPRVRAVAEAAGARFVEDTTPAGHPTPLNTALAAAEALLRADSPTVAVLHADLPVITAA
- (M2)       PDPRVRAIAEAEGVRFLEDDTPAGHPTPLNHALERAEAVLRAHTPNVCVLQADLPCVTPA
+ (H4)       PDPVVRAIADRAGVRWVEDTTPAGHPDPLNDALRAAVALLRPDSPRVAVLQADLPCVTAD
+ (M2)       PDPEVRAAAEDAGARFLQDDTPEGFPTPLNHALRQAAAWLRASVPNVAVLHADLPAVTAD
rSASA (H4)   RDPRVRAIAEAAGARFVADDTPPGHPDPLNAALAAAAALLRPACPAVAVLHADLAAVTPE
rSASA (M2)   RDPEVRAVAEDAGARWLEDDTPEGEPTPLNVALRAAAAELRPVTPNVAVLHADLATVTAD

             121                                                       180
WT           EFAAALAAARGHARSFVADRHGTGTAALFSTDGDLNPLFGIDSAQRHRDSGAIELTGDWP
- (H4)       ELAALFAAARAHPRAFVADAAGTGTTALFAFDCPLDPRFGADSVARHRAAGAVELTGDWP
- (M2)       ELRAAFAAARQHPRAFLADRAGTGTTALFAFNAPLAPQFGEDSVARHRAAGAVELTGDYP
+ (H4)       ELAAVFAAARGHPRAFLADRAGTGTTLLIAVDCPLDPRFGRDSVERHRAAGAVELTGDWP
+ (M2)       ELAALFAAARGHSRAFVADRHGTGTTALFVFDGPLDPRFGRDSAQRHRDAGAVELTGDFP
rSASA (H4)   ELATAFAEARRHPRAFLADAAGTGTTLLVSRDGRLAPRFGPDSVARHRAAGAHELTGDLP
rSASA (M2)   ELAAAFAAARGHPRAFLADRHGTGTTLLIALDCPLEPRFGRDSVQRHREAGAHELTADLP

             181                                                       204
WT           GLRCDVDTPEDLAVARELGAALPL
- (H4)       GMRVDIDTPEDLARARELGADFPW
- (M2)       GLRTDIDTPEDLAVARELGAAWPE
+ (H4)       GLRTDVDTPEDLAVARERGAPLPT
+ (M2)       GLRTDVDTPEDLARARELGAVLPL
rSASA (H4)   GLRTDVDTPEDLARARALGAAWPT
rSASA (M2)   SLRQDIDTPEDLAVARELGAVLPV
```

```
>Entry=B6I733 SUPFAM=SSF88697; Sites=21,24,74

              1                                                          60
WT            MQPNDITFFQRFQDDILAGRKTITIRDESESHFKTGDVLRVGRFEDDGYFCTIEVTATST
- (H4)        MKNNEISFLERFIPDILAGKKTVTIRDESESHYKVGDILKVYRKEDKSYFITIEITSVSE
- (M2)        MNKNEIRFFEKFLEPIKSGKKTITIRTESESHFKVGDILKVGKMNTDGYFVTVEVTSIST
+ (H4)        MKKNYITFYPRFEEDILNGKKTITIRDESESYLKVGDILKVKEKGVDGYFATIEITSIST
+ (M2)        MKDNEIDFFKRLIQPILDKKKTITIRDESESHYKVGDILKIGKMDEDGFFASAEITSIST
rSASA (H4)    MKNNYISFKERFEQPILEGRKTVTIRDESESHLKVGDILKIGKKLDDGVFATAEITSIST
rSASA (M2)    MKNNEISFFEELMQPILAKKKTITIRDESESHFKVGDVLKIGKMNTDGYFATVEITSIST

              61                                                        103
WT            ITLETLTEKHAEQENMTLTELIKVIADIYPGQTQFYVIEFKCL
- (H4)        ITWDEITEEHCEQECMTLEEFKKILAELYPGKTEFYVIEFKVI
- (M2)        ITWDEITEEHCIQECMTLEEFLKTLSDMYPGETKFYIIEFKVI
+ (H4)        ITLEEITEEHCEKECMTLEEFIKELSDLYPGETIFYVIEFKLI
+ (M2)        ITWNEITEEHAEQECLTLDELIKTLSDMYPGVTEFYVIEFKII
rSASA (H4)    ITLDDITEEMCEQECMTLDEFKKTLADLYPGETEFYVIEFKII
rSASA (M2)    ITFDEITEEMAEQECMTLEELKKALSDMYPGVTEFYVIEFKLI
```

```
>Entry=G4YEI5 SUPFAM=SSF52499; Sites=124,25,90

               1                                                          60
WT             MAALSSATKRLGRLIPHSSVLFVCDVQEVFRGLTFQLPTVIHGTNTMVSAAKLLNVPVVV
- (H4)         MAALSSATLKRTRLQAESAIVFICDMQESFRGLILNFDSVIDGCVFLVKVARLLNVPVVT
- (M2)         MSEEEEEEKRKSRLKAEDTLLFVVDVQEKFRGAILNFDKVIRGIVFAIKIAKLLDIPVVI
+ (H4)         MAEKKSELKKIPRLIAEESILFICDVQENFKGAIKNFDSVVEGINFLIEVAKLFNVPIII
+ (M2)         MSLESSEAKKIGRLEAEETILFVVDMQEKFRGAIRNFDLVVERCALLIKAAKLFDVPVIV
rSASA (H4)     MSAESSSVKKIGLLKAEESILFICDMQEKFRGHILNFDLVVQRVVRLIEICRMFNIPVII
rSASA (M2)     MNSENLKEKKLPRLKAEEAILFVCDVQEKFKGAIKNLDKVIEGVKFAIECCKLLNIPVIV

               61                                                        120
WT             TTQYGSRLGSTVSEISKNLEDAPDVKVFDKMKFSMLVPEVERHLTTNMPQRKSVLLCGIE
- (H4)         TTQYASKLGPLVPELTKELEDFPNTKHFDKTRFSMCIPEVLKHLDSNHPERKQVLLCGIE
- (M2)         TTQYAKKLGPIVPEISKELEDHPNVFTFDKLKFSMMTPEVEKHIEENFPDKKQVLLTGIE
+ (H4)         TEQYPKGLGPTVPEISSKLENYENVWRFSKTKFSMMIPEVENLLKEKYPERKQVILTGIE
+ (M2)         TEQYAKKIGPLLPEISSLLEDYPNSWTFDKTKFSMMIPEIEKYIEENLSEKKTVLLTGIE
rSASA (H4)     TTQYASKIGPILPELSKELENWDDVRSFDKIKFSMMIPEVERFIETEFPQRKQVLLCGIE
rSASA (M2)     TQQYGKKIGPILPEISELLENWPNAKVFEKTKFSMMIPEVEKYLEENYPNRKTVLVAGIE

               121                                                       180
WT             THVCVLQTCLDLLDKGYDVHVVSDAVSSSTSYNRSMALERIRQSGAYITSVESAIFQLAN
- (H4)         THVCVLQTVLDLLELGYRVFVVTDAVGSSTARDKSVALERMRQAGAYLVSVESAAFQLVE
- (M2)         THVCVLQTALDLMERGYEVFIVSDAIGSRTEENHNLALERLRQAGAKIVSTEMAIFELVE
+ (H4)         THICVYQTALDLLEKGYKVFIVSDAVSSRTEENKNTALERLRQAGAFIVSSEMAIFELVE
+ (M2)         THVCVLQTALDLLEKGYKVFIVTDAISSRTEEDHEVALERLRQAGAILTTTESAIFELWG
rSASA (H4)     THVCVLQTVLDLLEKGYQVYVVTDAISSRTALDRTVALERMRQAGAHLVSLESAAFELLE
rSASA (M2)     THVCVLQSVLDLLEKGYNVFVLEDAVSSRTEENKKIALERMRQSGAYIVSVESAVFQLME

               181                                                       210
WT             DAGNPEFKSISKLIKEHLKVQNGFDTGARI
- (H4)         DAGNPLFKAISKLVKERQKVINGKVTASLL
- (M2)         DAKNPKFKEVLKLIKEFKKEENSITSGSKK
+ (H4)         DAKNPKFKEVSKLIKKYKKKIKKITSGNNK
+ (M2)         DAKDPNFKELSKLIKSYKCILTGKDSGSNK
rSASA (H4)     DAGNPKFKDLLKLLKKYKKDRIAIVNGAKL
rSASA (M2)     DASNPKFKELTKLIKKRKLKKNGFDLGVKI
```

```
>Entry=O32858 SUPFAM=SSF55205; Sites=22

               1                                                          60
WT             LQPMAIALAAVADGTSMITENVFEARLAFVEEMIRLGADARTDGHHAVVRGIPQLSSAPV
- (H4)         MQPQLIALASLAEGTTVIVENVFEGRLMHVQELKRIGADIRVEGRYAVVRGVDKLTAAPL
- (M2)         MQPQLMALASIAEGTTIIVENVFEGRLSHIQELKRIGADARVEGNIAVIRGVPKLTAAPL
+ (H4)         MQPQIVALASLAPGTSIIVENVFENRMMYVQELQRIGADIRTEGNIAVIRGVDKLHAATI
+ (M2)         MQGQAVALASLADGTSIFVENVFEGRLSYIQELIKIGADARVDGKIAVVRGVPKLTAASL
rSASA (H4)     MQPQLIALASIAKGTSIIVENVFENRMMHIQELKRIGADIKTEGRYAVIKGVDKLTAAPL
rSASA (M2)     MQPQALALASIAKGTSIIVENVFEGRLSYVEEFKKIGADAKVDGKIAVVRGVDKLHAASL

               61                                                        113
WT             WSSDIRAGAGLVLAGLVADGETEVHDVFHIDRGYRLFVENLLSLGAEIERVGS
- (H4)         MATDLRAGAGLVLAGLVAEGETVVENIYHIDRGYENFEEKLKALGAKIERVGG
- (M2)         KATDLRAGAGLVIAGLVAEGETVIENIYHIDRGYENFEEKLKSLGAKIERVGE
+ (H4)         RATDLRAGAGLVLAGLVAEGETIVENVYHIDRGYENFVEKLQSLGANIERVGE
+ (M2)         RATDLRAGAGLVLAGLVAEGETVIENIYHIDRGYENFEDVLKSLGAKIERVGE
rSASA (H4)     KATDLRAGAGLVIAGLVADGKTEIHDIYHIDRGYENFEEKFKALGANIERVGG
rSASA (M2)     KATDLRAGAGLVLAALVAEGETVIENVYHIDRGYENFEEKLKSLGAKIERVGG
```

```
>Entry=P20605 SUPFAM=SSF140931; Sites=140,141,142,146,172,84,85

              1                                                         60
WT            MSDKFGEGRDPYLYPGLDIMRNRLNIRQQQRLEQAAYEMTALRAATIELGPLVRGLPHLR
- (H4)        MPSEYGEGKDPYLYPGSDILKNKLGIRDPEELEEAEAELIAERLEEIEFGPPDWGFEYLR
- (M2)        MSDYYGNKEDPYLYPGETVLRNKLNIRDEERLEEVEAELTALRAEEIDFGEEKFGFERLK
+ (H4)        MPYPYGEGYDPYLYPGTTVLRNKLDIRDQERLEEVEKELVALRLAEIEPGPFTYGFEYLK
+ (M2)        MSDLYSKEEDPYLYPGESVLRNKLDIRDAERLEEAERELIALRLAELEEGEPPRGLADLR
rSASA (H4)    MESPLGNYEDPYLYPGETILRNKLDIRDEEKLEEAEAELVALRLKTIDFGEKIWGFEYLK
rSASA (M2)    MSYNYGDGEDPYLYPGESILRNKFDIREQERLEEVEKELIALRLATIQPGPFNYGFEYLR

              61                                                       120
WT            TIHRQLYQDIFDWAGQLREVDIYQGDTPFCHFAYIEKEGNALMQDLEEEGYLVGLEKAKF
- (H4)        EIHKQLFQDLYDWAGELRTVNISQGDTPFALAAYIEREAEKLFAVLEEEDYLKGLEKEEF
- (M2)        EIHKHLFQDLYDWAGEVRTVNISQGDTPFCHYAYIEKEAEALFKKLEKENYLKGLEKAEF
+ (H4)        EIHKRLFQDIYDWAGQLRTVNIRQGDTPFCHYAYIEKEAEALFKQLEEEGYLVGLEKAEF
+ (M2)        EIHRRLFQDLYDWAGQLRTVDIRQGDSPFCLAAYIEREAEALFEELQEEGWLKGLEKAEF
rSASA (H4)    RIHKQLFEDLYDWAGQLRTINIAQGDTPFCLAEYLEEESKELFEELKKENYLKGLEKEEF
rSASA (M2)    EIHRQLFQDLYDWAGELRKVNIYQGDTPFCLYAYIEKEINALFKQLEEEGYLEGLEKAEF

              121                                                      180
WT            VERLAHYYCEINVLHPFRVGSGLAQRIFFEQLAIHAGYQLSWQGIEKEAWNQANQSGAMG
- (H4)        IERLAHYYAELNVLHPFREGSGRAQRVFMTELAKEAGWEMDWTGIPPEEMLQASIAAFTG
- (M2)        IEKLAEYYAELNVLHPFREGSGRAIREFIKELAEKAGYEVDFEGVEKEEMLQASIASFSG
+ (H4)        IERLAEYYAELNVLHPFREGSGRAQRVFMKKLALQAGYRLDWEGIEKEEMLQASIAAFQG
+ (M2)        VRRLAEWYAELNVLHPFREGSGRAQREFIRRLCLLAGYELDWEGIEREEMIQASIASFYG
rSASA (H4)    IEKLAEYYAELNVLHPFREGSGRAQREFFKRLAEEAGYEFDWEGIEKEEWLQAMIKSFTG
rSASA (M2)    VEKLAYYYSELNVLHPFREGSGRAQREFMKQLALKAGYLLDWEGIKREEMIQASKAAFQG

              181                                                      200
WT            DLTALQMIFSKVVSEAGESE
- (H4)        DLEPMQELLERVISKKGEVA
- (M2)        DLEPMKELFSRIISKKGEAE
+ (H4)        DLEPMKELFARCISEAGEDE
+ (M2)        DLAPMEELFARAIHEAGETE
rSASA (H4)    DLEPLKKLFEKAVKKKGEKE
rSASA (M2)    DLEPMKKIFARIISEAGESY
```

```
>Entry=P52484 SUPFAM=SSF46934;SSF54495; Sites=106

                 1                                                          60
WT               MSVSKLNKMQFSDKMSNLALARVTRKCKEVANASDITEAGIHVEIKENNLMDIKGFIKGP
- (H4)           MSVSKLVEKKKAEKALNMAKARVSREIKEINEDEDFKKLGLKVELINDDIFNIKAYIKGP
- (M2)           MSLEKLLKKIPSPKMSLKALKRLSKEIKDVAESEEVKKEGIKIEIINNNITDIIAYIKGP
+ (H4)           MSISKEEKKKKAEKVAKMALARIAKEVKETAEDAELAKEGISVKVKDNNLFDIEGYIKGP
+ (M2)           MSLSKLIKKLYSNKESNKALARIAKRLKEEANDPDLQKSGLKIEIKEDNVREVKGYIKGP
rSASA (H4)       MSVAKLVQKLKEEKEEQMALARISREVKDLLEDPSFKEAGLKAEVKEDNIFDVKGYIKGP
rSASA (M2)       MSVAYLVKKLKDTKNSNMALARRSKEVKTTANSSDLKESGIEIELKEDDITDITGKIKGP

                 61                                                        120
WT               EGTPYAGGTFEIKVDIPEHYPFEPPKAKFVTRIWHPNISSQTGTICLDILKDKWTASLTL
- (H4)           EGTPYAGGTFELKIDIPKEYPFKPPKVKFTTKIWHPNISSETGAICLDILKDKWTAALTI
- (M2)           EGTPYEGGVFEFEIKIPPEYPFKPPKVKFKTKIWHPNISFQNGAICLDILKDKWTPALTL
+ (H4)           EGTPYAGGTFKLKISIPLEYPFKPPKVKFKTKIWHPNISSTTGAICLDILKDKWTPALTI
+ (M2)           EGTPYAGGTFNLKIEIPEEYPFKPPKVKFVTRIWHPNISSQTGTICLDILKDKWTAALTI
rSASA (H4)       EGTPYAGGVFKLEISIPKEYPFKPPKVKFTTKIWHPNISSQTGAICLDILKDKWTPALTL
rSASA (M2)       EGTPYAGGTFDVKIDLPEEYPFKPPKVKFSTKIWHPNISSQTGTICLDILKDKWTPALTI

                 121                                                       180
WT               RTVLLSLQAMLCSPEPSDPQDAVVAKQFINNYPMFTATAVYWTSYFANSKKDVEPDFNRK
- (H4)           KTVLLSIQSLLSSPEPDDPQDADIAKLYKTNKPLFNSVAKAWTAKYATSKKEVPDDFEKK
- (M2)           KTVLLSLKSLLSSPEPDDPQDADIAKIYKTDKELFESTVKYWTAKYANAKDDVKAVYPEE
+ (H4)           KTVLLSLQSLLSSPEPDDPQVAEIAKVYKNDREKFEKIAKIWTEKYALNKKEEIPEEKKK
+ (M2)           KTVLLSIQSLLTSPEPDDPQDADIAKVYKTNRPKFNETASRWTAKYAMPKQTVELPDEKL
rSASA (H4)       KTVFLSLQSLLSSPEPSDPQDADIAKLYLENRPLFEATAKYWTLKYTENKKEIEPSEEKK
rSASA (M2)       KTVLLSLQSLLCSPEPDDPQDAVVAKQYKDDYTKFVETAKFWTNYYANSKLEIKDDYNKK

                 181                                                       214
WT               VGRLIEMGIRETEAIVYLSCNNWKLEQALQFIFD
- (H4)           ISKFEEMGFKKEDAIRYLAVVNWKVDETLEKFFS
- (M2)           VKKLIEMGISEEDAIKYLLENNWNLEKALEKILS
+ (H4)           IDKFIEMGFKKEEAEEKLALNNWKLEEAVNKILE
+ (M2)           ISRFLEMGIKKDEAISYLALNNWKFEETIEKIFS
rSASA (H4)       LGRLEEMNVKKEDAIIYLAANNWKLEETVKKILS
rSASA (M2)       ISRFEEMGIKREEAIKYLALNNWKLEEALDKILS
```

```
>Entry=Q06909 SUPFAM=SSF88946;SSF88659;
Sites=39,40,41,42,43,44,45,46,47,48,49,50,51,52

              1                                                          60
WT            MERFRDGAQDAFEDLFARHAPRVQGFLARMVRNGALAEDLLQATFLSVIRSRGRYEPGTR
- (H4)        MLRFRDGAEDAFEVLYARLAPRVLGVLLRLTRDAAEAEDLLQATFLSVIRSRGRYEPGTR
- (M2)        MLRVREGDEDAFRVLYDRHAPRILGYLSRMVRDGALAEDLLQATFLSVIRSRGRYEPGTR
+ (H4)        MERFRDGDEDAFEELYARLAPRVHAFLARAVRDGALAEDLLQATFLSVIRSRGRYEPGTR
+ (M2)        MERFRTGAEAAFEELYARYAPRVHGFLARLTRNAADAEDLLQATFLSVIRSRGRFEPGTR
rSASA (H4)    MLRFRDGDEPAFEVLYARLAPRVLGFCARMTRDGAEAEDLLQATFLSVIRSRGRYEPGTR
rSASA (M2)    MQRFRDGDETAFEELYARHAPRIYAYLLRLTRDAALAEDLLQATFLSVIRSRGRYEPGTR

              61                                                        120
WT            FIPWLMTIAANAARDALRHQRHVDAYASREDTATPASAAPDDSDPSLRRHLLDALQQLHP
- (H4)        FEPWLYTIALNAVRDARRRRRRETARAARLDDAVPAPAAPDDAVAGERLLLESALAELEP
- (M2)        LEPWLFAIATNVARDAFRKRRREQARVDRLDDVVPASVPDELADPAERREVLEVLEQLEP
+ (H4)        FDAWLMTIAANAARDELRRRRRERARLERLEDVPPAPAAPDPADPARRRRVLEVLERLEP
+ (M2)        FEAWLFTIAANAARDLHRRRRRELARALRLDVAETPVSAPDVADVDERRRLVECIEELEP
rSASA (H4)    FEPWLFTIAANEARMALRRRRREEARAALEEVAVDAPAAPDDTPPARRRWLLECLEQAKP
rSASA (M2)    FEAWLYTIAANAARDALRERRRVLARAARLDVVVPAAAAPDDADPAERRRLLEVLEELEP

              121                                                       174
WT            DHREAVVLSKVEGWSFEEIGALRGISPGAARLRAHRGYEKLRELLGELELEVAR
- (H4)        DQREAVVLHHLEGMSHVEIAAMLGTSEGASRQRAHRGLLRLRELLGEDEEDRAR
- (M2)        VLRDAVVRHYVEGRSFEEIGAEQGTSAGAARMRAHRGLQRLREMLGLRLLLEAL
+ (H4)        EQREAVVQTKLLGRSPEEIGAEWGTSPGAARMRAHRGLLRLRELLGELEEEEAR
+ (M2)        EQREVVVLTKVEGRSHEEIGAERGASPGAARVRAHRGLLRLRECLGERREEEAR
rSASA (H4)    EQREAVVATYLEGRSFEQAAAELGTAPGAMRMRAYRGLERIRECFGEKLEELAR
rSASA (M2)    DLREAVVQAYVEGRSPEEIGAERGVSAGAARMRAQRGLARLRELLGEREEEEAR
```

```
>Entry=Q21YG7 SUPFAM=SSF54975; Sites=20,38

               1                                                          60
WT             MADVTYRLVICGLVQGVFYRGSMVSRANALGLRGWVRNRLDGSVEAVVQGEATEVNRMVE
- (H4)         MSQVTRRLRITGRVQGVFFRASVVEQAEELGLKGWVRNRRDGSVEAVVQGEEELVEQMIE
- (M2)         MSKITKKLIITGRVQGVFFRASMVEKAESLGLKGWVRNRRDGSVEAVIQGEEDKVEKMIE
+ (H4)         MEIVTYKLKITGRVQGVFFRYSTVEKALELGLKGWVRNRRDGSVEAVVQGEEEKVEKFIE
+ (M2)         MAQVTRRLTITGKVQGVWFRASMVSRAQAMGLRGWVRNRYDGSVEAVVQGEAPEVERFIE
rSASA (H4)     MSMVSYRLVITGKVQGVWFRASMISRANSLGLKGWVRNRRDGSVEAEIQGESDLVEKMIE
rSASA (M2)     MAKISYRLIITGKVQGVFFRASTIARANALGLRGWVRNRLDGSVEAVVQGEAALVEKMIE

               61                                                         92
WT             WARRGPSNAVVTSVNIFPSEGDFVGFQLREST
- (H4)         WCRQGPPVARVESVEVEEAEGDFSGFEQRPTV
- (M2)         WCHKGPPLAVVENVEVEDAEGDFNGFEQKPTV
+ (H4)         WCKKGPPLAEVEKVEVEEAEGEFEGFEILPTV
+ (M2)         WCRKGPPLAEVTSVEVEPAEGDFEGFEIRESV
rSASA (H4)     WCWKGPPTAEVTSVEIYPSEGEFNGFERRESV
rSASA (M2)     WCRKGPPVAEVESVEIFPAEGEFEGFEQRPTV
```

```
>Entry=Q54KN7 SUPFAM=SSF52833; Sites=30,31,32,33

               1                                                          60
WT             MYKEPKNESEYEAELKNAPVAVVYLTATWCGPCRAIAPVFTNISNAPENSKITFFKVDVD
- (H4)         MYHVPKDEAEFKALLKNSPVALVDFSATWCGPCKAIAPVFDELSNAPENSKVTFFKVDVD
- (M2)         MYFEPKDESEFKAELKNAPLALVKFSATWCGPCKAIAPLLDELSNAPENSKLTFFKVDVD
+ (H4)         MYHEPKNESEFHSLLENSPTALVKFSATWCGPCRAIAPFFDELSNAPENSKLTFFKIDID
+ (M2)         MYIEPKNESEFKSLLKNSPNALVDFFATWCGPCKAIAPIFDELSSAPENSKLTFFKVDID
rSASA (H4)     MYHEPKNEAEFKALLKNAPVAVVDFFATWCGPCKAIAPVFAELSNAPENAKITFFKVDID
rSASA (M2)     MYIIPKNESEFKSLLKQSDNAIVYFYATWCGPCKAIAPFFDELSTAPENSKITFFKIDID

               61                                                        105
WT             ALKKLPVCESLQGVPTFIAYRNGEEQERFSGANKVALENMVKKLL
- (H4)         ALEKLPELESLEGLPTFITYKNGEEVDRFSGANKQALKEAVKKLL
- (M2)         ALKELPEVQSLQAIPTFIAYKNGQEVERFSGANKTALKNIVKKLL
+ (H4)         SLKELPEIQSLQAIPTFITYKNGEEIDRFSGANKDGLENAVKKLL
+ (M2)         ALEELDEVKGLEGIPTFIAYKNGEEVERFSGANKTGLKDAVKKLL
rSASA (H4)     ALKELPELQGLQGIPTFAFYKNGELVERFSGANKPALQEGIKRLL
rSASA (M2)     ALEELEENQNLKGIPTFIAYKNGEEVERFSGANKPALQDMVKKLL
```

```
>Entry=Q602T2 SUPFAM=SSF55068; Sites=54

                 1                                                           60
WT               MWFKSAGSALPDPHDALPGRATPLQVTETHFVHGRRIRPPFPSGTARAFFGMGCFWGAER
- (H4)           MALLPSSLSLVNKEDALPGRSTPIEVAPTHLVTGNQLKPPFPAGTAKAMFGLGCFWGAER
- (M2)           MFFLKKKPPLAAPEDALPGRDTPLEVNPTHLVNGRPLLPPFPEGTARAMFGMGCFWGAER
+ (H4)           MFLKPKSYFLPAKEDALPGRPEPMEVAPTHLVNGRPLLPPFPPGTAKAYFGLGCFWGAER
+ (M2)           MSLLSRKLTLPDREDALPGRPEPIEVEPTHLVTGRRIRPPFPPGTARAMFGLGCFWGAER
rSASA (H4)       MALLLSGHAMAAPEDALPGRDTPLTVAETHHVTGRQLLPPFPPGAAKAMFGMGCFWGAER
rSASA (M2)       MKKKRDLTRLAAKEDALPGRDEPMEVPDTHFVNGNPLKPPFPEGTEKIEFALGCFWGAER

                 61                                                         120
WT               RFWTLPGVYSTAVGYAGGSTPNPTYDEVCTGRTGHAEVVLVVYEPDKVGFDALLQVFWTS
- (H4)           KFWSLPGVYSTAVGYAGGFTPNPTYEEICSGRTGHTEVVLVVYEPDKIGYAELLKVFWES
- (M2)           KFWSVPGVYTTAVGYAGGHTPNPTYEEMCTGRTGHTEVVLVVYETDKVSYADLLKIFWES
+ (H4)           KFWSLPGVYSTAVGYAGGFTPNPTYEEVCSGRTGHTETVLVVYEPDKVSLAELLKVFWEA
+ (M2)           KFWQLPGVYTTAVGYAGGFTPNPTYEEICSGRTGHTEVVLVVYEPDRVGYDALLKVFWES
rSASA (H4)       KFWTLPGMYTTAVGYAGGFTPNPTYEEMCSGRTGHTEVVLVVYDPDKVSLATLLKVFWES
rSASA (M2)       KFWQLPGVYSTAVGYQGGFTPNPTYEEICSGRTGHTEAVRVVYEPEKVSLETLLKVFWES

                 121                                                        180
WT               HDPTQGMRQGNDVGTQYRSVIFATTPEQLQWALVSRDAYERALERAGRGGITTEIRMAPD
- (H4)           HDPTQGMRQGNDVGTQYRSVIYASSPEQLKLAEESRDAYQKALEKAGKGGITTEIRPAPP
- (M2)           HDPTQGMRQGNDVGTQYRSAIYASSPEQLELAELSKELYQEGLEKAGLGPITTEIALAPT
+ (H4)           HDPTQGMRQGNDVGTQYRSAIYATTPEQLALAEASKAAYQEALEKAGLGPITTEIEPAPP
+ (M2)           HDPTQGMRQGNDVGTQYRSAIYAATPEELERAHASRDAYQKALEKAGLGAITTEIRPAPE
rSASA (H4)       HDPTQGMRQGNDVGTQYRSAIYAATPEQLAQAEASRAAYQAALDKAGLGPITTEIAMAPP
rSASA (M2)       HDPTQGMRQGNDVGTQYRSAIYAHSPEQLKLAEASRAAYQKALEKAGKGAITTEIAMAPP

                 181                                                        222
WT               FYYAESYHQQYLAQHPNGYCGLRGTGVECPPFRIEQSRADPI
- (H4)           FYYAEDYHQQYLAKNPNGYCGLRGTGVKFPPLKLEEAKAHHH
- (M2)           FYYAEDYHQQYLAKNPNGYCGLGGTGVCCPPIDLEELAAPAA
+ (H4)           FYYAEDYHQQYLAKNPNGYCGLGGTGVECPPLEIPAAHALAL
+ (M2)           FYYAEDYHQQYLAKNPNGYCGLRGTGVRFPDLRLEESGAPAA
rSASA (H4)       FYYAEDYHQQYLAKNPNGYCGLRGTGVCLPDPLPEAAWATRL
rSASA (M2)       FYYAEDYHQQYLAKNPNGYCGLGGTGVAMPELELAAAAALPH
```

```
>Entry=Q6G4Y4 SUPFAM=SSF55811;
Sites=52,53,54,55,56,57,58,59,60,61,62,63,64,65,66,67,68,69,70,71,72,73

               1                                                          60
WT             MDAVVNLTTLPYRRSVGILVFNHEGKVWVGRRLMVCIHEDTKIYHRWQLPQGGIDENEEP
- (H4)         MTTKINYDDLPYRKCVGVMVINEEGKVFIARRINFDPRFGDELENYWQFPQGGIDENEEP
- (M2)         MNSDFNKDDLPYRKCVGVMVMNEEGKIFVGRRIIPTVHEDDKEQLLWQMPQGGIDENEEP
+ (H4)         MTSTIDIENLPYRKNVGVMVINEEGKVFIGRREKVELKENEYIENWWQMPQGGIDENEEP
+ (M2)         MVSIPNYDTLPYRKNVGVMVFNEEDKVFIAKRKKDVEYKNVKLKEWWQMPQGGIDENEEP
rSASA (H4)     MTSKINYDTLPYRKCVGVMVINDENKVFVGRRIIETKEKDVEVENQWQMPQGGIDENEEP
rSASA (M2)     MTSTMNYDDLPYRKCVGVMVINDEGKVFIGRRLIKDEKENVKRENWWQMPQGGIDENEEP

               61                                                        120
WT             LDAARRELYEETGIRSIELIKEAKYWFHYDFPQEIVGSVLGSKYRGQIQKWFAFQFTGEL
- (H4)         LDAARRELYEETGIKSVELVKESKEWYRYDLPEELVGKVWGGKYRGQEQKWFLFRYTGKE
- (M2)         LDAARRELYEETGIKSAELLKESEEWLKYDLPKELIGTVWGSKYRGQEQKWFLFRFTGEE
+ (H4)         LDAARRELYEETGIKSVELIKESKEWLKYDLPKELLGKVWGSKYRGQEQKWFLFRFTGEE
+ (M2)         LDAARRELYEETGLKSVELIKESEEWLYYDLPEELLGKIFGSKYRGQEQKWFAFRYTGEE
rSASA (H4)     LDAARRELYEETGIRSVELVKESKEWLRYDLPEELLGTAFGGKYRGQEQKWFLFRYTGEE
rSASA (M2)     LDAARRELYEETGIKSAELLKESKEWLKYDLPKELLGSVWGSKYRGQEQKWFLFRFTGED

               121                                                       173
WT             SEIKINPPPDGHKAEFDQWKWVDLETLPSIVISFKKHVYMKIVNEFRGSFKGL
- (H4)         SEIQLVPVPTGKKPEFDEWKWVELEDLPSYIVPFKKEVYEKVVEEFKGLWKGW
- (M2)         SEIQLNPPPDGHEPEFDEWKWIDLDELPSLIVPFKKEVYKKVVEEFKGLEKGK
+ (H4)         SEIKLNPPPTGKKPEFDEWKWVDLEELPNLIVPFKKEVYKKVVEEFKGVIKGK
+ (M2)         SEIQLNPVPDGHEPEFDEWRWEDLDTLPSLIVPFKRDVYKKVVEEFKGCFKGK
rSASA (H4)     SEVKLNPIPTGHKPEFDEWKWVDLDTLPSLIVPFKREVYKKVVEEFKGEEKGK
rSASA (M2)     SEIQIVPPPNGHEPEFDEWKWVDLDDLPNLIVPFKKEVYKKVVDEFKGIEKGL
```

```
>Entry=Q6G5F0 SUPFAM=SSF56420; Sites=136,137,140,94

                1                                                          60
WT              MPMRPLVIVPDPILREISKPVEYIDSAVQKLADDMLETMYHAQGVGLAAIQIGIPLRMLV
- (H4)          MAIRPIIKIPDPQMRLISKPIEKIDSSIHKIIDDMFETMYEAKGIGLAAIQIGIPLRLFV
- (M2)          MATRDIITVPNPLMREISEPVEKFDSSIQKIIDDMFDTMYEAKGIGLAAIQIGIPKRILI
+ (H4)          MPKRPIVTVPDPKLREISKPIEKIDASIHKLIDDMFETMYEAKGIGLAAIQIGVLKRIFV
+ (M2)          MAIRPIITVPDPKLREISKPVEKIDSSIHKIIDDMFETMYEAKGIGLAAIQIGIPLRLFV
rSASA (H4)      MPIRDIITIPDPRLREISKPVEKIDASIHKLIDDMFETMYEAKGIGLAATQIGVPLRIIV
rSASA (M2)      MAIRDIIEIPDPFLRKISEDIEKFDSSIQKLIDDMFETMYEAKGIGLAAIQIGIPLKLFV

                61                                                        120
WT              LDVSRNDEQKNPLVIINPEVLWLSDERNIYKEGCLSIPEYFAEVERPKRLCVRYQNREGK
- (H4)          LDVAEEDEEKNPLVVINPEILKTSDEKSVYEEGCLSIPDIYAEIERPERITIRYLDREGK
- (M2)          IDLSKEDEEKNPIVMINPEILWVSDEKCIYEEGCLSIPDIYADIERPKRLTIRYLDRNGK
+ (H4)          LDVSENDEEKQPLVMINPEILETSDEKTIYEEGCLSVPEYYAEIERPKKITVRYLDRNGK
+ (M2)          MDLHKEDEEKNPLVMINPEILETSDEKCIYEEGCLSVPDYYAEIERPKKVTVRYLDRNGK
rSASA (H4)      MDVSKEDEEKNPMVIINPEFVELSDEKQIYEEGCLSVPEYYAEIERPKKITVRYLDREGK
rSASA (M2)      LDITETEEEKEPLVFINPTLLEESDEKQIYEEGCLSIPDYYAEIERPKKITVKYLDRNGK

                121                                                       176
WT              QTEIEADDLLATCLQHEIDHLNGRLFIDYLSKIKRDMVIRKFKKRAKEKNTQEAVL
- (H4)          LTEIEADDLLAVCFQHEIDHLNGKLFIDYLSKLKRDRIIRKFKKLAKEEVDELSIF
- (M2)          ECEIEADDLLAVCIQHEIDHLNGKLFIDYLSKLKRDRIIRKFKKMAKEEVEEEAVL
+ (H4)          EVEIEADDLLAVCIQHEIDHLNGKLFIDYLSRLKRERILKKLKKRAREKDAEEAVL
+ (M2)          EVEEEFTDLLATCIQHEIDHLNGKLFIDYLSKLKRDMILKKFKKLAKEKDDELALL
rSASA (H4)      EVEIEADDLLAVCIQHEIDHLNGKLFIDYLSRLKRERIIRKFKKLAKEKVEEKAEE
rSASA (M2)      ETVITADDLLATVIQHEIDHLNGKLFIDYLSKLKRDMIIKKFKKKAKKKVEELSIL
```

>Entry=Q6NLQ3 SUPFAM=SSF110004; Sites=116,155,69,73

```
              1                                                          60
WT            MKRKRYEMEKKKKTEIQTAIEELSVFIVTKPADKTEATHIPLRPILSFCSLIIQVLDKIG
- (H4)        MKIKKKKKKKKKKTILEEANEELSVFENVKPSNKNIPRKIPLKPLLEVLNKILEILDKLG
- (M2)        MKKKKKNLNKKKKSKLEEINEKLSKYTNVKESDLDESYKFPLKPIIELGEYITEGLEKLG
+ (H4)        MLKKLFKKKKKEKTRLEELNEKLAKYLIPEKANPNLPLELPLKPLLELAEEIIEILEKMG
+ (M2)        MEKKKKKKKKKKKTKLDELNDKLSKYIPTYDPDKDEPEELPIKPLIELFKFIIELIDKMG
rSASA (H4)    MEEKLLEELKKEKTKLEEIIEELSKYELNNIKDKSELEKIPLKPIIKLLEEILKILDKLG
rSASA (M2)    MLSKLLSKLKEKKSKLDELIEQLSQYKLDEPADLEKAKQIPIKPLLDLYNELIEVLDKLG

              61                                                        120
WT            PTMAVLRQDIDQNIQRLEKFYETDSCVYSNLAEILKKEKEEGTSKMVASCGRALFWLTRT
- (H4)        PAFSIVKKDLEKNIEKLEKRYKENPSKYDTIEKLLEKEKENGNLLKDDSASKALLWTSRG
- (M2)        PVFSIVREDIEINIKRLKKKLEENPDVYDSINKIIEKEKEEGTIKKEDSTARALWWLQRS
+ (H4)        PAFAPVKQDLEENLEKLKKRYEENPEKYSTIEKILKKEKEDGTLEKEDSTAKALWWLTRG
+ (M2)        PVFSPTKEDLQENIEKLEKRYEENPEEYNTFSKIFEKEYEEGTIKKKDSVARAVWWSIRG
rSASA (H4)    PALSLVKKDIEENLEKLKKRLEEDPNKYNTFDKIIEKEKEEGTLKKEDSTAKALEWLLRG
rSASA (M2)    PALSTVREDIQENVERLQKRYEEDPSKYSTFPQILEHEYENGTLLKDDSVARALFWLTRH

              121                                                       180
WT            MDFTAGLLRLLSKEMSSKMEELVEECYMTTLKPHHGWIASAAFKVCLKLVPDNKTFMEAI
- (H4)        LEFVKNILKLLSKPQSEKLKKIVKEAYEETLKPYHGFFTNNVVKIALKFLPDKETFLNSI
- (M2)        LEFIQAILKELTKDLSGKFKEIVKKAYEETLKPYHGFLTSTAVKIALKFVPDKKTFLEVL
+ (H4)        LEFVVGVLKKLTKRDDVKMEEIVLEAYEKTLKPYHGKISNAAFKIALKFVPDRKTFLENI
+ (M2)        LEFIVAVLKKVTEEEDVEMKEIFEKAYEETLKPYHDWITNTAFKVALKFAPDRETFFENL
rSASA (H4)    LEFVKAFLKKLSENLDKKTKEIFEEAYNETLKPYHGFLVNKAFKAALKFAPDNKKLLEAI
rSASA (M2)    LEFIALVLKELTKNLDSKTKEIVEKAYEKTLKPYHGFITNAAFKVALKFVPDRKTLFEAL

              181                                                       223
WT            GARDESYDTLREDIDTLSSLLTPILKEIYFVLEQYGLSRLRSM
- (H4)        SPKNKSEEEVKKELKTFSDKIEPLLKKINQELEKYGLTWEKYK
- (M2)        GGKEEDIEEIKKIFNEFYNQLVPILKLIKLNLEKYGLGKLEIK
+ (H4)        GGKDKPFEEMKKILNEFVDNMKPILKKIHKVLEKYGYTYEKMK
+ (M2)        GWKDEDKEYYKKEINEFVDLVKPYIKEVKKILKKYGLGKLKKE
rSASA (H4)    GAKNNDYERFKKTVNEFVNLIEPIIKRIYIVFEKYGIKRPKAK
rSASA (M2)    GAKPEGEEELKKTLNEFSDIIEPILKAIYIELEKYGYTWEKSK
```

```
>Entry=Q74K70 SUPFAM=SSF52141; Sites=65

              1                                                          60
WT            MKKLIGNDWDEVLAPAFESEKYQELRDFLKEEYQTKQIFPDMYHIFTAFKLTSFADTKVV
- (H4)        MKKIIGNEWDEKLAPAFESEKFVELKEFLKEEYKTKKIYPPMKDIFNAFKKTPYDNVKVV
- (M2)        MKKLLGNNWDEVLAPAFESEKFEELKEFLKEEYKTHKIYPPMKDIFNAFKYTSYDNVKVV
+ (H4)        MKKIIGTDWDEVLAPAFESEKFQELKEFLKEEYKTHKIYPPMKDIFNAFKYTSYDDVKVV
+ (M2)        MKKLLGNDWDEALAPAFESPKFEELKAFLKEEYKTKKIYPPMKDIFNAFKLTPFDKVKVV
rSASA (H4)    MAKIIGNDWDEVLKPAFESEEFKKLKEFLKEEYKTKKIYPPMKDIFNAFKYTPYDDVKVV
rSASA (M2)    MKKILGNDWDEALAPAFEDPKFEELKEFLKEEYKTKKIYPPMKDIFNAFKLTPYDKVKVV

              61                                                        120
WT            ILGQDPYHNPGQATGMSFSVNPGVPLPPSLKNIYKELYDDVGAVPVNHGYLKKWADQGVL
- (H4)        ILGQDPYHNPGQAHGLSFSVNPGVPIPPSLKNIYKEIYDDIGVEPVEHGYLEKWADQGVL
- (M2)        ILGQDPYHNPGQAHGLSFSVLPGVPLPPSLKNIYKELYDDIGFEPQKHGYLKKWAEQGVL
+ (H4)        ILGQDPYHNPGQAHGLSFSVNVGVPIPPSLKNIYKELYDDLGIEPVDHGDLTYWAEQGVL
+ (M2)        IIGQDPYHGPGQAHGLSFSVKPGVKIPPSLKNIYKELHDDLGIEIVPHGYLEKWAEQGVL
rSASA (H4)    ILGQDPYHGPGQAHGLSFSVKPGVKLPPSLKNIYKEIESDLGVKPLKHGYLEKWAEQGVL
rSASA (M2)    ILGQDPYHGPGQAHGLSFSVLPGVKMPPSLKNIYKELEDDIGVKPVDYGYLEEWAKQGVL

              121                                                       180
WT            LLNAVLTVPYGNANGHRGKGWELITDTAIKALSDRGKVVFILWGKFAQNKIPLIDEGKNV
- (H4)        LLNAVLTVEEGNAASHKGKGWELITDQAIKALSEKGKVVFILWGKYAQDKIPLIDESKNT
- (M2)        LLNAVLTVREGEANSHKGKGWELITDTAIKALSEKGNVVFLLWGKYAQSKLPLIDESKNV
+ (H4)        LLNTVLTVREGKANSHKGKGWEIITDKAIKALSEKGNVVFLLWGKYAQKKLPLIDTSKNT
+ (M2)        LLNTVLTVEEGKPNSHQGKGWEHITDKAIKALSEKGNIVFILWGKYAQSKLPLIDKSKNT
rSASA (H4)    LLNTVLTVREGKPASHQGKGWELITDTAIKALSERGNIVFVLWGKYAQKKLPLIDTEKNT
rSASA (M2)    LLNTVLTVEEGKANSHKGKGWEHITDTAIKALSEKGNVVFLLWGKYAHKKIPLIDTSKNE

              181                                                       231
WT            IIKSPHPSPFSADRGFFGSRPFSRCNDALIKFGESPIDWQLPQEVNPADMA
- (H4)        IIKAAHPSPLSAHRGFFGSKPFSKTNEALIKQGKSPIDWKLPKEIDPSLLS
- (M2)        IIKSAHPSPLSAYRGFFGSKPFSKVNEALKKLGKEPIDWQLPKEVDPLDYL
+ (H4)        IIESAHPSPLSAYRGFFGSKPFSKVNEALIKQGKEPIDWQLPKIILPSEYA
+ (M2)        IIESAHPSPLSAYRGFFGSKPFSKVNEALKKQGLEPIDWKLPEKIDEEDFL
rSASA (H4)    VIKSAHPSPLSAYRGFFGSKPFSKVNEALKEQGLEPIDWQLPEEIDPSLIK
rSASA (M2)    IIKSAHPSPLSAYRGFFGSKPFSKVNEALKKLGKEPIDWELPKEVDMEKYK
```

```
>Entry=Q7SIG3 SUPFAM=SSF50494; Sites=187,45,59,61,64,66,69,93

               1                                                          60
WT             VVGGRVAQPNSWPWQISLQYKSGSSYYHTCGGSLIRQGWVMTAAHCVDSARTWRVVLGEH
- (H4)         IVGGQEATPHSRPYQVSLQYKSGSSYYHTCGASLIKENWVITAAHCLNNNYTYRVVLGEH
- (M2)         IVGGYEAIPHSRPYQVSLQRKSGSSWYHICGGTLIREDWILTAAHCVNVSYTYRVVLGEH
+ (H4)         IIGGKEAIPHSHPYQVSLQYKSGSSWYHFCGGTLIREDWVLTAGHCVNSSYTYRVVLGEH
+ (M2)         IVGGEEAEPHSRPYQVSLQYKSGSSWYHTCGATLIREDWILTAAHCVNASYTYRVVLGEH
rSASA (H4)     IIGGYEAEPNSWPWQVSLQYKSGNSWYHTCGATLIRENWILTAAHCMNTSYTYRVVLGEH
rSASA (M2)     IIGGTEATPHSWPYMVSLQYKSGSSYYHICGATLIKPNWVITAAHCVDASYTYRVVLGEH

               61                                                        120
WT             NLNTNEGKEQIMTVNSVFIHSGWNSDDVAGGYDIALLRLNTQASLNSAVQLAALPPSNQI
- (H4)         NLSTNEGTEQYKDVTEIYLHPSYNPNNLSGGYDIALIKLSSNATFTSSVQLISLPPANKT
- (M2)         NLTTNEGYEQYINVTSVYLHPGYNSNDLSGGYDIALLRLSTNATLNSAVNLASLPPANLE
+ (H4)         NLTTNEGYEQYRDVDATYIHPGYNSNDLTGGYDIGLLRLNTKATLTSAVQLASLPPANYV
+ (M2)         NLTTNEGYEQIRDVVSTYIHPGYNPDDLSGGYDIALLRLNQNATLTSGVQLASLPPENLE
rSASA (H4)     NLTTNEGTEQVRDITSVYIHPGYNPNDLLGGYDIALVRLNTNATLTSGVQLASLPPQNRE
rSASA (M2)     NLSTNEGTEQVRDVTSIYLHPGYDPNNLTGGYDIALLKLSTNATLNSSVQLASLPSANTE

               121                                                       180
WT             LPNNNPCYITGWGKTSTGGPLSDSLKQAWLPSVDHATCSSSGWWGSTVKTTMVCAGGGAN
- (H4)         LPENTTCWVTGWGRTSEGGPLSDTLQEASVSMIDYATCTSSSWYGSTIKPTMICAGGGST
- (M2)         LPENTSCYVTGWGRTSDGGPLSDVLQEAYVPTIDYSTCTSSSYWGSTVKPTMLCAGVGSV
+ (H4)         VPENTSCYVTGWGRTSEGGPLSDVLQEAYVPTVDYATCTSSSWWGSLIKPTMVCAGGGSV
+ (M2)         ISENTSCWVTGWGRTSNGGPISDVLQEAYLPTIDYATCTSSSWYGSTIKTTMICAGGGSE
rSASA (H4)     FPENTTCWVTGWGRTSEGGPLSDVLQQAYVPLLTYSTCTSSSWYGSTVKETMICAGLGSV
rSASA (M2)     LSDNVDSYVIGWGRTSEGGPLSDTLQEAYVPYIDYSTCSSSSWYGSTILPTMLCAGEGSK

               181                                                      236
WT             SGCNGDSGGPLNCQVNGSYYVHGVTSFVSSSGCNASKKPTVFTRVSAYISWMNGIM
- (H4)         DSCQGDSGGPLVCEENGTWYQYGIVSFGSSSGCNAPKKPGVYTRVSNYHSWINSIL
- (M2)         SACNGDSGGPLLCEVNGSWYQYGITSFVSSSGCNTPKKPTVFTRVSNYLSWINGIL
+ (H4)         SACNGDSGGPLFCEVNGSWYVYGITSFGSSSGCNAPKKPGVYTRVSNYLSWINGII
+ (M2)         SACNGDSGGPLMCEFNGSYYVYGIVSFVDPSGCNVPKKPTVFTRVSNYLSWINGIL
rSASA (H4)     SACNGDSGGPLMCEENNKWYQYGITSFVSSSGCNAPKKPTVFTRVSNYLSWINGII
rSASA (M2)     SACQGDSGGPLLVEVNGSYYVYGIVSFGDSSGCNVPKKPTVYTRVSNYLSWINSII
```

```
>Entry=Q81ST8 SUPFAM=SSF102588; Sites=113,12,15

              1                                                          60
WT            MSGLHILAFGAHADDVEIGMAGTIAKYTKQGYEVGICDLTEADLSSNGTIELRKEEAKAA
- (H4)        MKPLDILAFGAHPDDVEIGMAGTIAKYTKKGKKVGICDLTKAELSTRGTLEIRKKEAEKA
- (M2)        MEGLHILAFGAHPDDVELGMAGTLAKYTKKGYKVGICDLTNAELSTRGTIEIRKEEAKAA
+ (H4)        MEGLDILAFGAHPDDVELGMAGTLAKYAKQGKNVGIVDLTKAELSTRGTVEIRIEEAKAA
+ (M2)        MESLDILAFGAHPDDVEIGMAGTLAKYTKKGKKVGIVDLTKAELSSRGTVEIRKEEAKKA
rSASA (H4)    MEGLDILVFGAHPDDIEIGMAGTIAKYTKQGKKVAICDLTEGELSSRGTLEIRKEEAKAA
rSASA (M2)    MSGLDILAFGAHPDDIELGMAGTLAKYAKQGKEVAIVDLTEAELSSRGTVEIRKEEAKAA

              61                                                        120
WT            ARIMGVKTRLNLAMPDRGLYMKEEYIREIVKVIRTYKPKLVFAPYYEDRHPDHANCAKLV
- (H4)        SKILGVKVRLNLDIPDRAFFDKEEYKKKIVKVIRKYKPKIVFIPYIEDRHPDHANCHKLV
- (M2)        SKILGVNFRINLDIPDRGFEDKEEYKKKIIKVIREYKPKIVFTPYWEDRHPDHSNASKIV
+ (H4)        SKILGVKVRLNLDIPDRAFFDKEEYIKKIVEVIRTYKPKIVLAPYFEDRHPDHGNASKLI
+ (M2)        SKILGVKERINLDIPERSFKDKEEYIKKVTKIIRKYKPKLVLAPYWEDRHPDHGACSKIV
rSASA (H4)    SKILGVHARLNLNLPDRGFEDKEEYKKEIIKVIRTYKPKIVFTPYWEDRHPDHGAASKIV
rSASA (M2)    SKILGIKARINLNIPDRSFKDKEEYKKKIIEVIRTYKPKIVFIPYWEDRHPDHGYCSKIV

              121                                                       180
WT            EEAIFSAGIRKYMPEVPPHRVESFYHYMINGFHKPNFCIDISEYVSQKVEALEAYESQFS
- (H4)        KDAIFLSGLRKLFPEIPPHRVENVYYYFINANLKPDFLVDISEYIDKKIEAIKAYKSQFD
- (M2)        EEAIFLSGLRKYLPELPPHRVENVFYYIQNGFPKPDFLVDISEYIDLKIKSLKAYKSQFS
+ (H4)        QEAIFVSGLRKYLPELPPHRVDAFYYYFINGFPKPDFVVDISEYMDLKIEALKAYKSQFT
+ (M2)        KEAIFVSGLKKYFPEIPPHKVENFLYYFINGFEKPDFLVDISEYIEQKIEALKAYKSQFS
rSASA (H4)    EEAIFVTGLRKVFPEIPPHRVEAFYYYIINGFHKPDFYVDISEYIDTKIEALKAYESQFS
rSASA (M2)    EEAIFVSGLRKYMPELPPHRVEAVYYYLVNGYFKPDFVVDISEYIDKKIESIKAYKSQFT

              181                                                   234
WT            TGSDGVKTPLTEGYVETVVAREKMFGKEVGVLYAEGFMSKKPVLLHADLIGGCK
- (H4)        PNSKGPKTPLNNGYIEAIEARDKYFGRLIGIKYAEGFISEKPIIIKSDLLGGLK
- (M2)        TSSDGIETPLNNGYIENIIAREKYFGRLIGVEYAEGFISKKPILIKSDLLGGLK
+ (H4)        DGLDGPETPINVGFIEVIEARAKYLGRQIGVEYAEGFISLRPIIINDDLFGGLK
+ (M2)        TGPDGEETPLTNGYIDAILARAKYFGKLIGVDYAEGFISEKPIIIKSNIFSNLK
rSASA (H4)    EGSDGVETPLNNGYIETVIARAKELGRLIGVEYAEGFISKKPIKLISDFFGDIK
rSASA (M2)    DGFDGVETPINNGFIETILAREKELGRTIGVEYAEGFISKKPLIIKGSLLGGNL
```

```
>Entry=Q8X6Y0 SUPFAM=SSF51391;
Sites=108,134,135,136,137,166,186,187,37,38,39,40,41,69,70,89

                1                                                          60
WT              MYQPDFPPVPFRLGLYPVVDSVQWIERLLDAGVRTLQLRIKDRRDEEVEADVVAAIALGR
- (H4)          MELPALPQLPKKLGFYPVVDSVEWIQKLFDLGVKTLQLRIKDRKDEEIEAEIEAAIALAK
- (M2)          MIQSPYPPLPQRLGLYPVVDSVEWIEKLLNLGVKTLQLRIKDKKLEEIEAEITAAVALAK
+ (H4)          MKTLPLPQLPKYLGLYPVVDSVEWIEKLFNLGVKTLQLRIKDRKTDEIEPIIIAAVALAK
+ (M2)          MKQSPFPPLPQRLGLYPVVDSVEWMEKLLELGVKTLQLRIKNQKDEEIEPIIEAAIALAK
rSASA (H4)      MLQSPFPALPKRLGLYPVVDSVEWIEQLFALGVKTLQLRIKDRKVEEIEAEIVAAIALAK
rSASA (M2)      MIQASFPPLPKRLGLYPVVDSVEWMEKLFNLGVKTLQLRIKDKKLEEIEAEIAAAVALAK

                61                                                        120
WT              RYNARLFINDYWRLAIKHQAYGVHLGQEDLQATDLNAIRAAGLRLGVSTHDDMEIDVALA
- (H4)          KYNALLFINDYWKLAIKYQADGVHLGQEDLKATDLLALRTAGLKLGISTHNDEEVEEALA
- (M2)          KYNAQLFINDHWKLAIKHQAYGVHLGQEDLKATDLDAIKAAGLKLGISTHNDQEVDLALA
+ (H4)          KYNALLFINDYWQLAIKYQADAVHLGQEDLLATDLQALIDAGIRLGISTANDEEVDQALA
+ (M2)          KYNALLFINDYWKLAIKYKADGVHLGQEDLKATDLQALIDAGLRLGISTHNEEELELALA
rSASA (H4)      KHDAYLFINDYWQLAIKHQAFGVHLGQEDLKATDLDALKAAGLRLGISTANDEEIELALA
rSASA (M2)      KYNARLFINDYWQLAIKHQADGVHLGQEDLKAADLPAIHAAGLKLGISTHNDEELEIALA

                121                                                       180
WT              ARPSYIALGHVFPTQTKQMPSAPQGLEQLARHVERLSDYPTVAIGGISLARAPAVIATGV
- (H4)          AKPSYIAFSHVFPTQTKIHPSAPQGLEKLSKYVQRLSDYPTVAIGGISLARLPAVLATGV
- (M2)          AKPSYIAIGHVFPTQTKQMPWAPQGLEQLSKLVQRLADIPTVAIGGISLARAPAVLAAGV
+ (H4)          AQPSYIAIGHVFPTQTKQMPWAPQGLEKLAKYVERLSHIPTVAIGGISLARAPAVLATGT
+ (M2)          AKPSYIALGHVFPTQTKQMPSAPQGLEKLAKWVERLSHIPTVAIGGISLARAPAVLATGV
rSASA (H4)      ARPSYIAFGHVFPTQTKQMPDAPQGLEKLAQYVQRLAHIPTVAIGGISLARAPAVLATGV
rSASA (M2)      ARPSYIALGHVFPTQTKQMPWAPQGLEKLAKWVERLSHIPTVAIGGIDLARAPAVLATGV

                181                                                       211
WT              GSIAVVSAITQAADWRLATAQLLEIAGVGDE
- (H4)          QSAAVVSGIIKAADPKQAVAEYLKITGTSLL
- (M2)          PSMAVVSAIIQAADPKQAIAQFQQICGIGNE
+ (H4)          SSMAVVSAIIQAADQKQAVAEFLKICGVGQE
+ (M2)          QSIAVVSAITQAADPKQAIAQFLQLTAESSK
rSASA (H4)      DSVAVVSAITQAADPKQAVAEFLKICGIGTL
rSASA (M2)      DSIAVVSAVTQAADPKQAVAQFLQICGLGLE
```

Figure S7. Multiple sequence alignment of ESM3-generated proteins before and after HEIWA modification. Proteins are arranged alphabetically by UniProt accession. - and + indicate the absence and presence of the HEIWA module, respectively. “rSASA” indicates the use of reversed SASA gate. Each *de novo* generation was performed with two random seeds, “H4” and “M2”. Conserved residues were highlighted in yellow. No disruption of residue conservation patterns was observed, consistent with HEIWA acting as a fine-tuner of the embeddings.

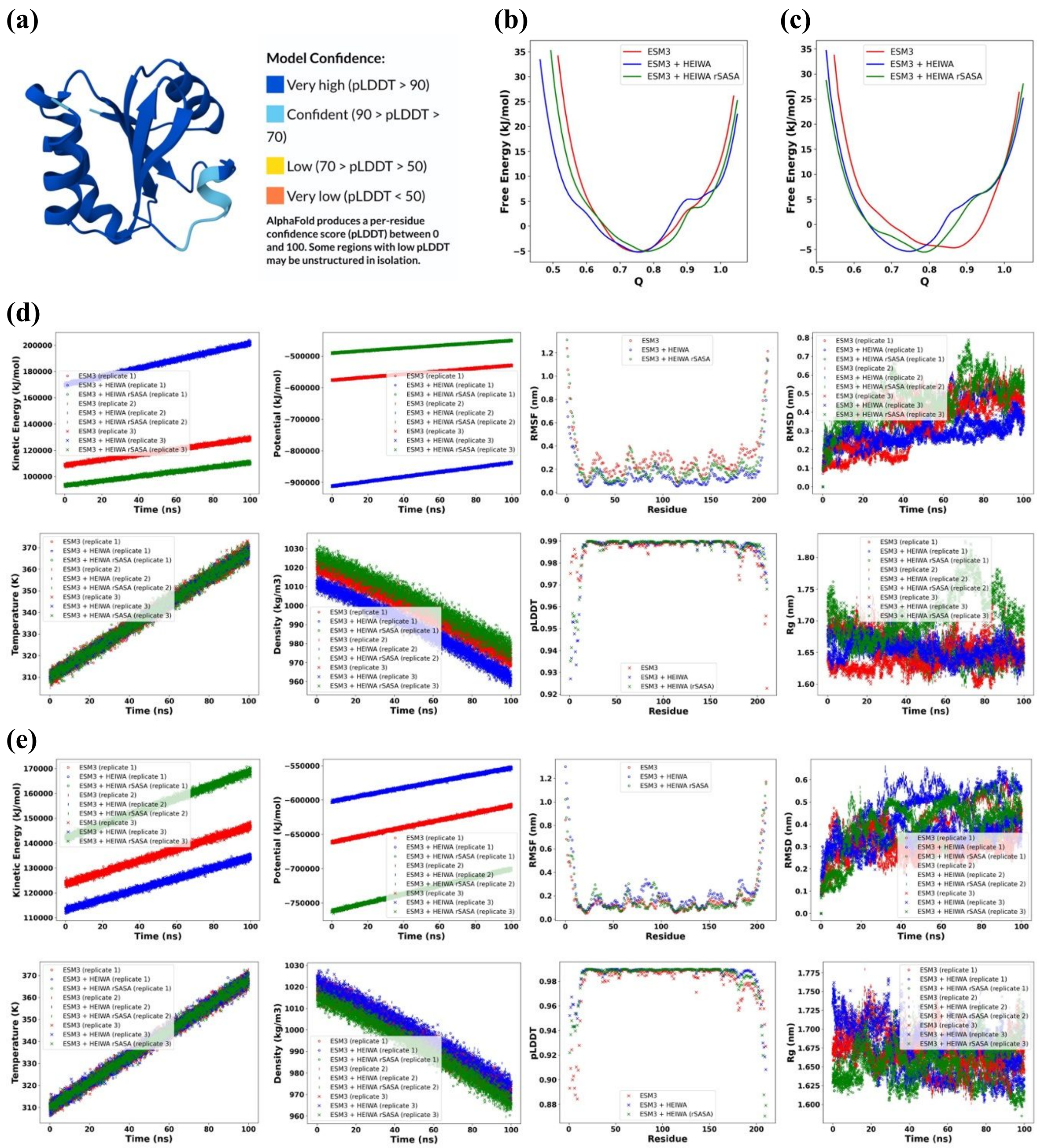

Figure S8. Gromacs molecular dynamics results for G4YEI5, including (b,c) metadynamics and (d,e) temperature ramping where RMSF was calculated as the average of all replicates. pLDDT was calculated using the ESM3 pipeline. These results are based on *de novo* proteins generated by ESM3 using two random seeds, H4 for (b,d) and M2 for (c,e). For H4 (d), full HEIWA resulted in significantly lower RMSF than the ESM3 baseline (paired Wilcoxon $p < 0.001$) and with the reversed SASA gate (paired Wilcoxon $p < 0.001$). The reversed SASA gate resulted in significantly lower RMSF than the ESM3 baseline (paired Wilcoxon $p < 0.001$). For M2 (e), full HEIWA resulted in significantly higher RMSF than the ESM3 baseline (paired Wilcoxon $p < 0.001$) and with the reversed SASA gate (paired Wilcoxon $p < 0.001$). RMSF of the reversed SASA gate did not differ significantly from the ESM3 baseline (paired Wilcoxon $p > 0.05$). The first 15 and last 10 residues were excluded from the paired Wilcoxon test.

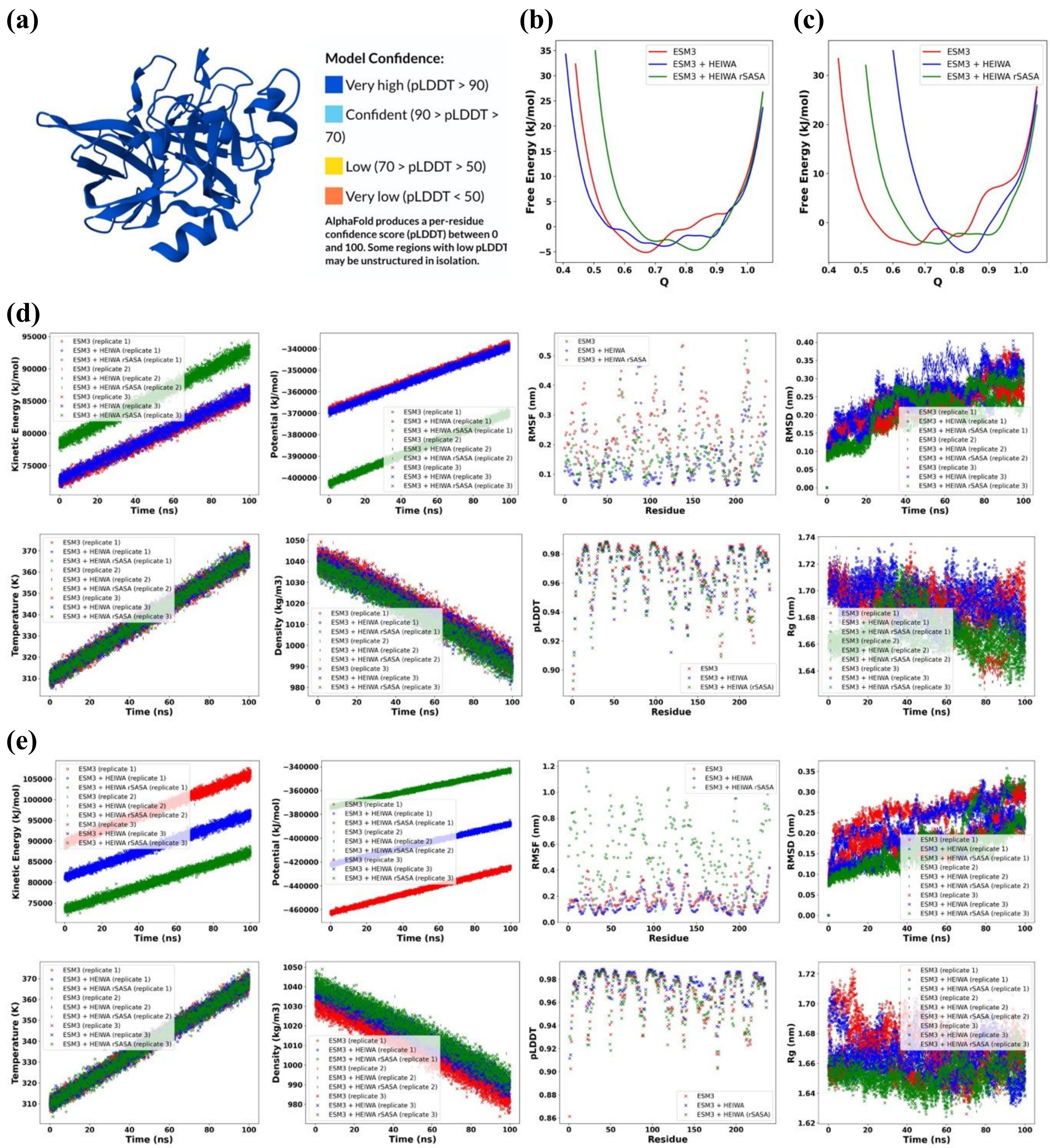

Figure S9. Gromacs molecular dynamics results for Q7SIG3. For both random seeds (d,e), full HEIWA resulted in significantly lower RMSF than the ESM3 baseline (paired Wilcoxon $p < 0.001$) and with the reversed SASA gate (paired Wilcoxon $p < 0.001$). For H4 (d), the reversed SASA gate resulted in significantly lower RMSF than the ESM3 baseline (paired Wilcoxon $p < 0.01$). For M2 (e), the reversed SASA gate resulted in significantly higher RMSF than the ESM3 baseline (paired Wilcoxon $p < 0.001$).

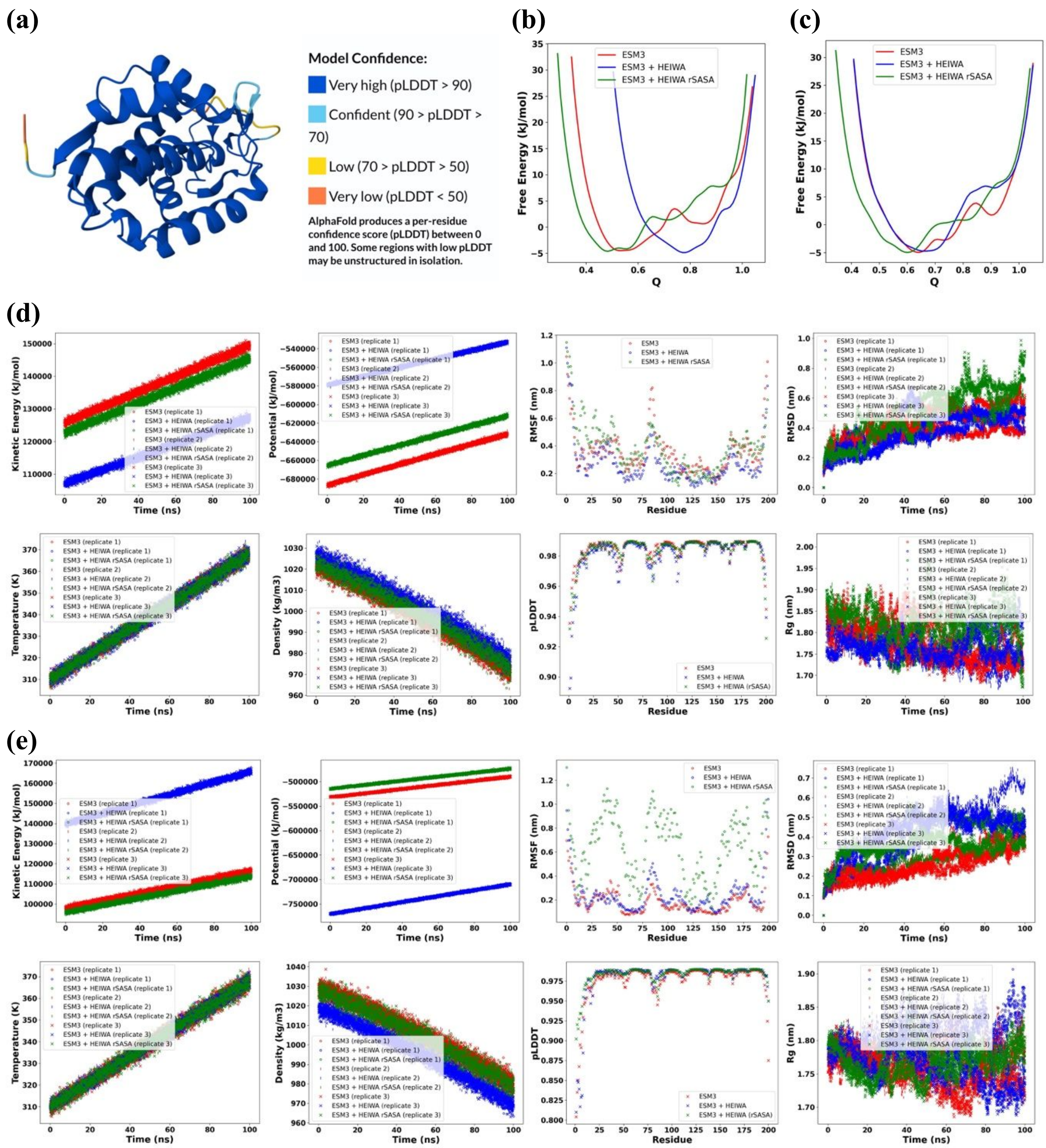

Figure S10. Gromacs molecular dynamics results for P20605. For H4 (d), full HEIWA resulted in significantly lower RMSF than the ESM3 baseline (paired Wilcoxon $p < 0.001$) and with the reversed SASA gate (paired Wilcoxon $p < 0.001$). The reversed SASA gate resulted in significantly lower RMSF than the ESM3 baseline (paired Wilcoxon $p < 0.01$). For M2 (e), full HEIWA resulted in significantly higher RMSF than the ESM3 baseline (paired Wilcoxon $p < 0.001$), but significantly lower than RMSF with the reversed SASA gate (paired Wilcoxon $p < 0.001$). The reversed SASA gate resulted in significantly higher RMSF than the ESM3 baseline (paired Wilcoxon $p < 0.001$). The first 15 and last 10 residues were excluded from the paired Wilcoxon test.

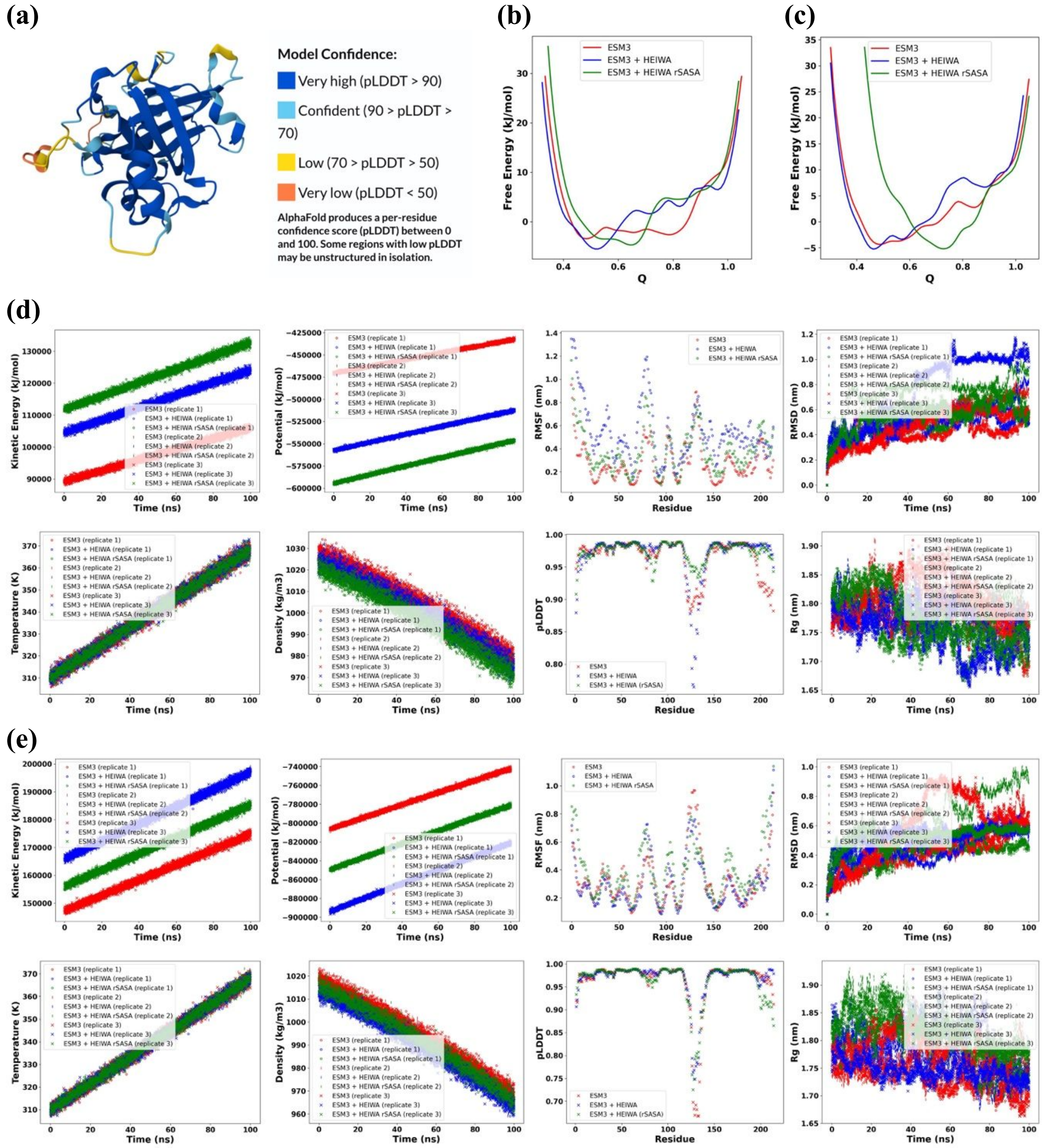

Figure S11. Gromacs molecular dynamics results for A1C603. For H4 (d), full HEIWA resulted in significantly higher RMSF than the ESM3 baseline (paired Wilcoxon $p < 0.001$) and with the reversed SASA gate (paired Wilcoxon $p < 0.001$). For M2 (e), full HEIWA resulted in significantly lower RMSF than the ESM3 baseline (paired Wilcoxon $p < 0.001$) and with the reversed SASA gate (paired Wilcoxon $p < 0.001$). For both random seeds (d,e), the reversed SASA gate resulted in significantly higher RMSF than the ESM3 baseline (paired Wilcoxon $p < 0.001$).

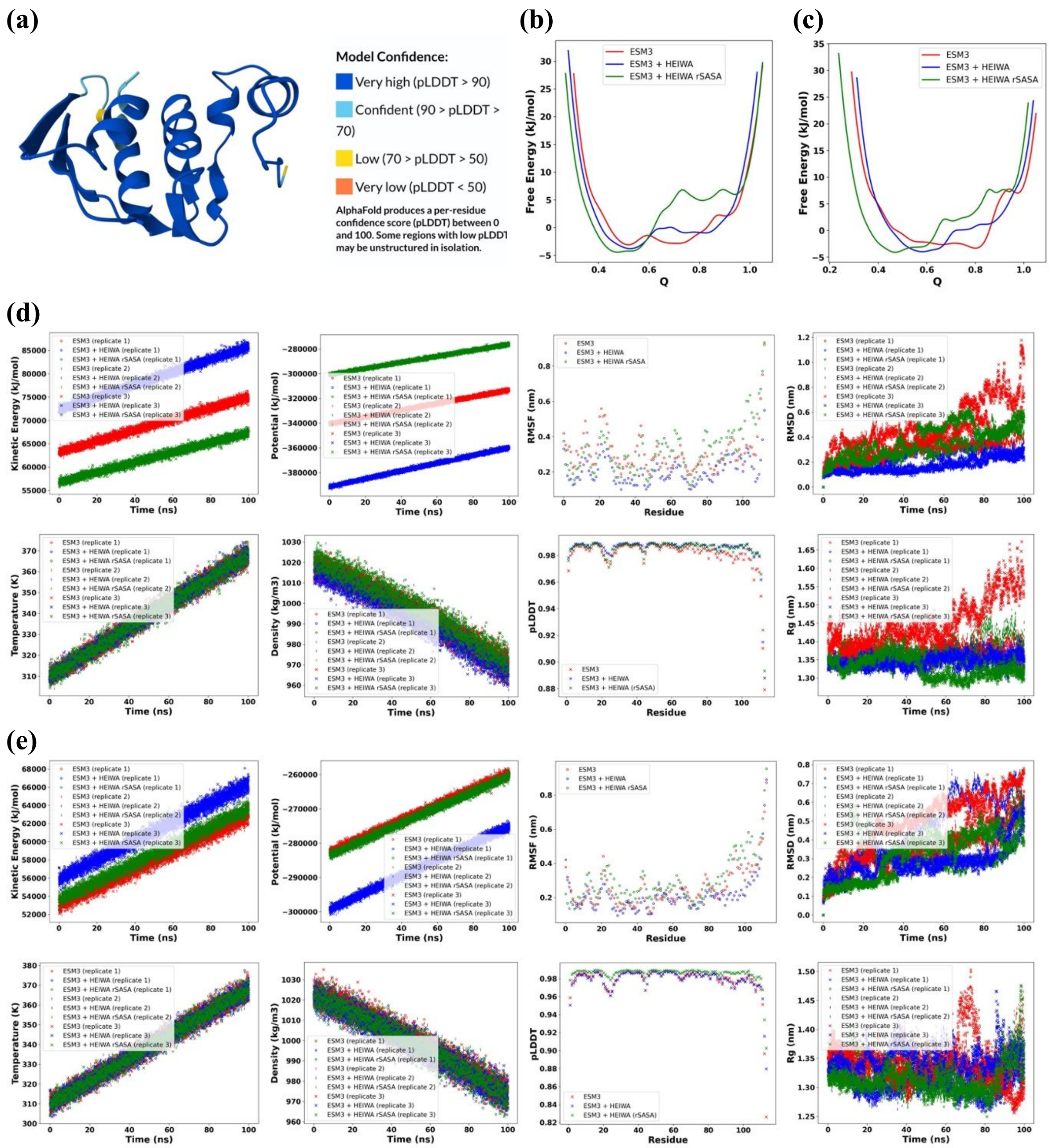

Figure S12. Gromacs molecular dynamics results for O32858. For both random seeds (d,e), full HEIWA resulted in significantly lower RMSF than the ESM3 baseline (paired Wilcoxon $p < 0.001$) and with the reversed SASA gate (paired Wilcoxon $p < 0.001$). For H4 (d), RMSF of the reversed SASA gate did not differ significantly from the ESM3 baseline (paired Wilcoxon $p > 0.05$). For M2 (e), the reversed SASA gate resulted in significantly higher RMSF than the ESM3 baseline (paired Wilcoxon $p < 0.001$). The last 5 residues were excluded from the paired Wilcoxon test.

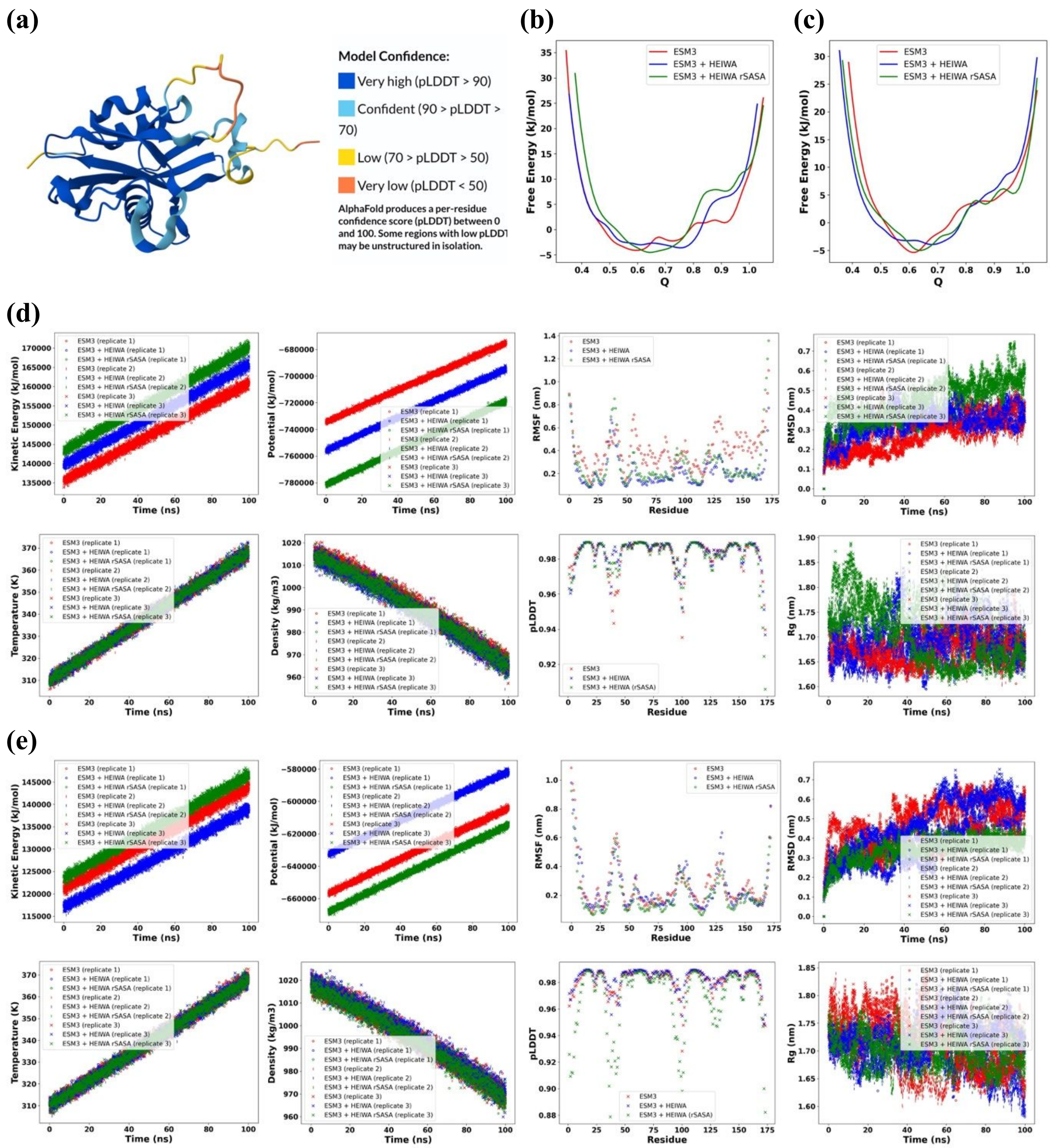

Figure S13. Gromacs molecular dynamics results for Q6G4Y4. For H4 (d), full HEIWA resulted in significantly lower RMSF than the ESM3 baseline (paired Wilcoxon $p < 0.001$) and with the reversed SASA gate (paired Wilcoxon $p < 0.001$). For M2 (e), full HEIWA resulted in significantly lower RMSF than the ESM3 baseline (paired Wilcoxon $p < 0.05$) but significantly higher than RMSF with the reversed SASA gate (paired Wilcoxon $p < 0.001$). (d,e) For both seeds, the reversed SASA gate resulted in significantly lower RMSF than the ESM3 baseline (paired Wilcoxon $p < 0.001$). The first 5 and last 5 residues were excluded from the paired Wilcoxon test.

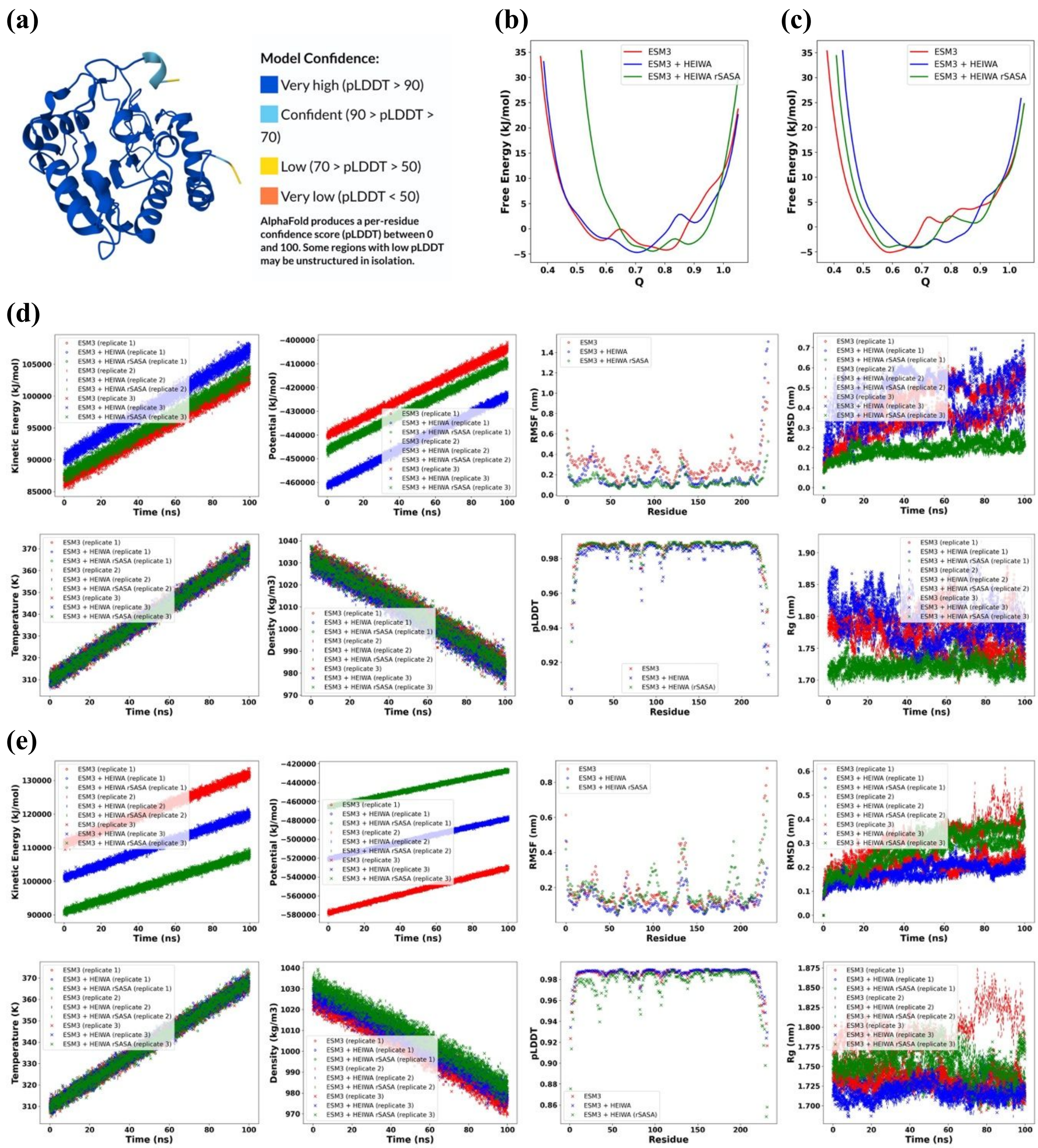

Figure S14. Gromacs molecular dynamics results for Q74K70. For H4 (d), full HEIWA resulted in significantly lower RMSF than the ESM3 baseline (paired Wilcoxon $p < 0.001$), but significantly higher than RMSF with the reversed SASA gate (paired Wilcoxon $p < 0.001$). The reversed SASA gate resulted in significantly lower RMSF than the ESM3 baseline (paired Wilcoxon $p < 0.001$). For M2 (e), full HEIWA resulted in significantly lower RMSF than the ESM3 baseline (paired Wilcoxon $p < 0.001$) and with the reversed SASA gate (paired Wilcoxon $p < 0.001$). The reversed SASA gate resulted in significantly higher RMSF than the ESM3 baseline (paired Wilcoxon $p < 0.001$). The first 5 and last 10 residues were excluded from the paired Wilcoxon test.

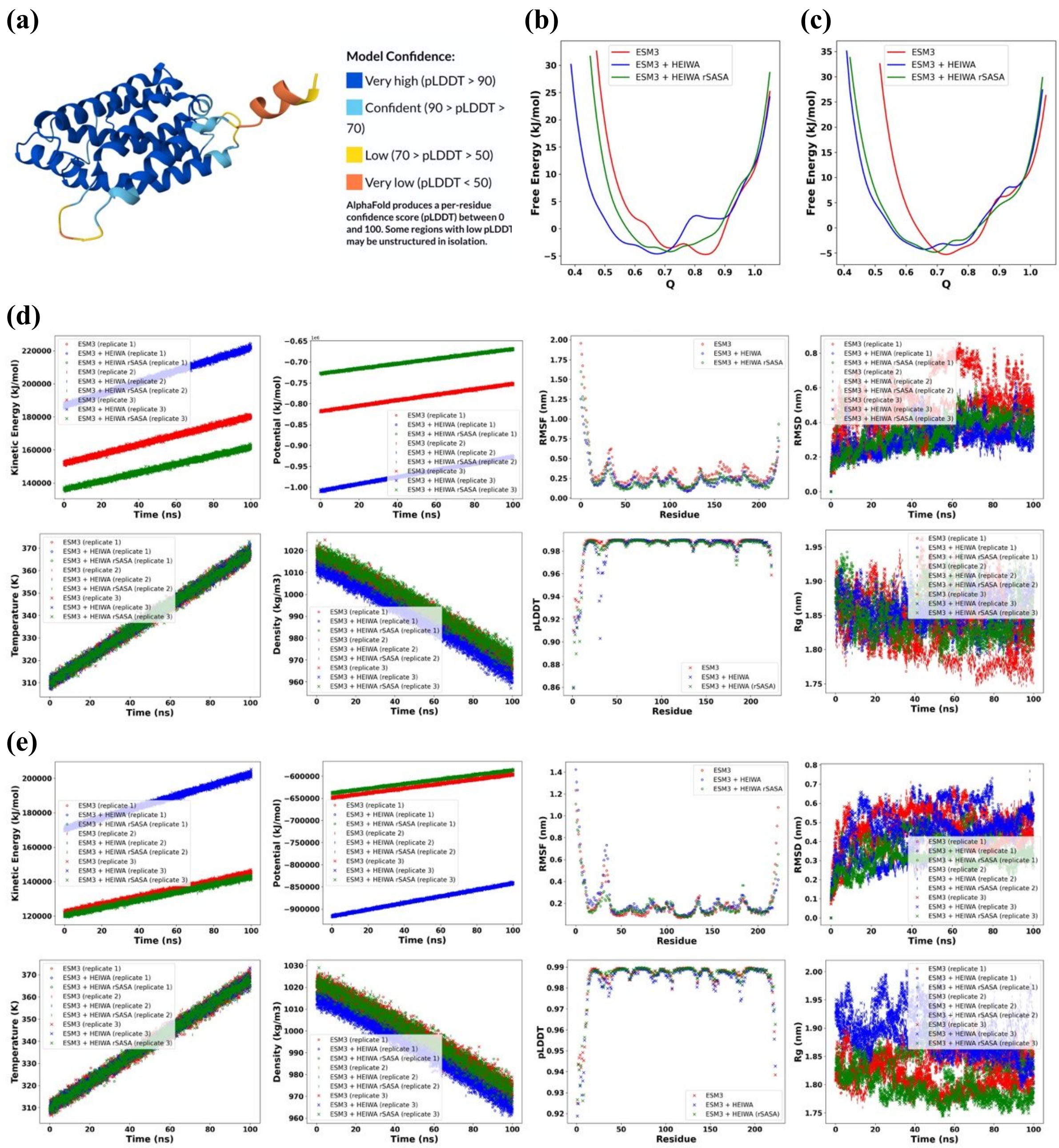

Figure S15. Gromacs molecular dynamics results for Q6NLQ3. For H4 (d), full HEIWA resulted in significantly lower RMSF than the ESM3 baseline (paired Wilcoxon $p < 0.001$) but did not differ significantly from the reversed SASA gate (paired Wilcoxon $p > 0.05$). The reversed SASA gate resulted in significantly lower RMSF than the ESM3 baseline (paired Wilcoxon $p < 0.001$). For M2 (e), full HEIWA resulted in significantly higher RMSF than the ESM3 baseline (paired Wilcoxon $p < 0.001$) and with the reversed SASA gate (paired Wilcoxon $p < 0.001$). The reversed SASA gate resulted in significantly higher RMSF than the ESM3 baseline (paired Wilcoxon $p < 0.01$). The first 10 and last 5 residues were excluded from the paired Wilcoxon test.

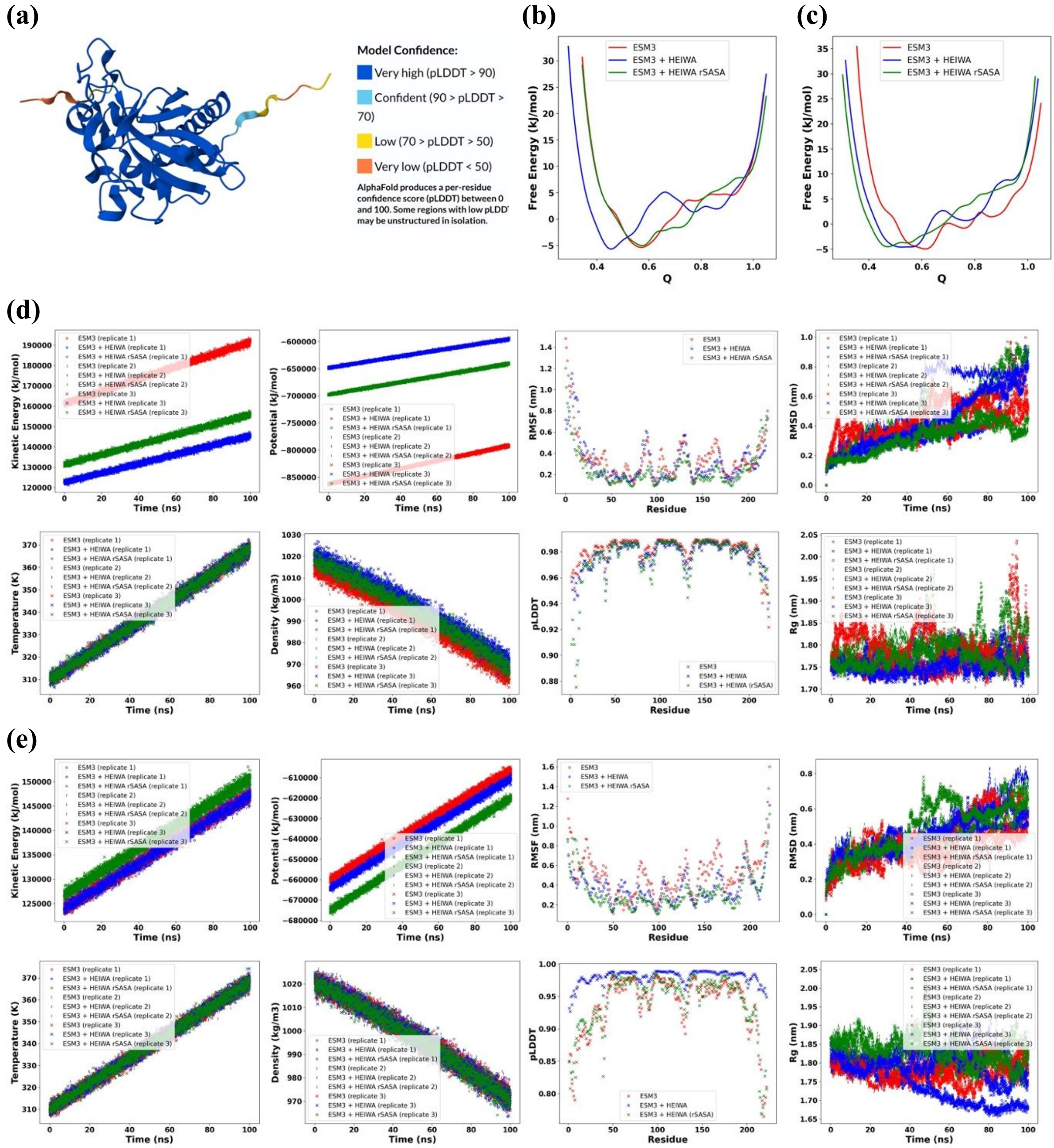

Figure S16. Gromacs molecular dynamics results for Q602T2. For both random seeds (d,e), full HEIWA resulted in significantly lower RMSF than the ESM3 baseline (paired Wilcoxon $p < 0.001$) but significantly higher than RMSF with the reversed SASA gate (paired Wilcoxon $p < 0.001$), and the reversed SASA gate resulted in RMSF significantly lower than the ESM3 baseline (paired Wilcoxon $p < 0.001$), excluding the first 5 and last 10 residues.

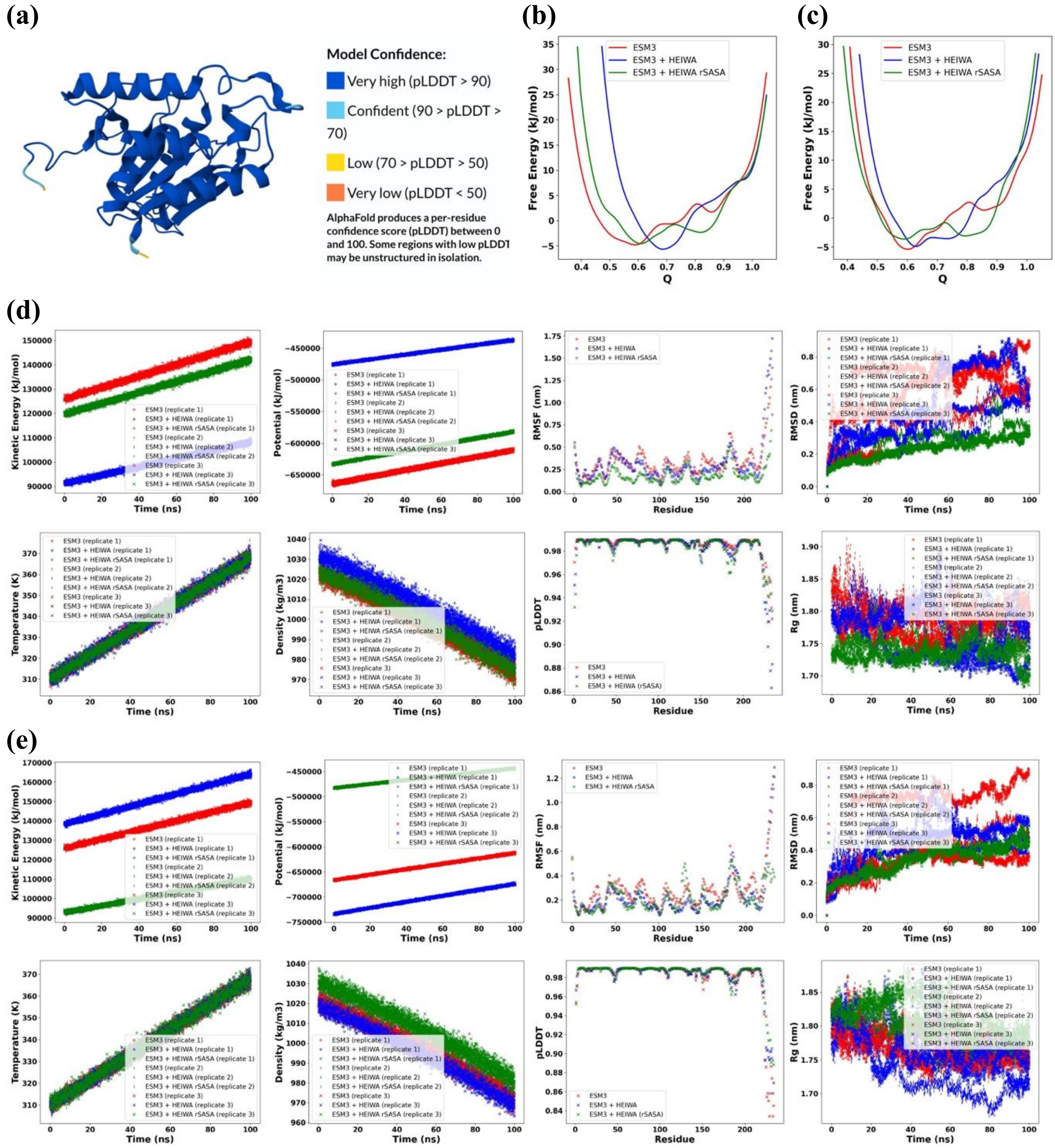

Figure S17. Gromacs molecular dynamics results for Q81ST8. For H4 (d), the RMSF of full HEIWA did not differ significantly from the ESM3 baseline (paired Wilcoxon $p > 0.05$) but was significantly higher than with the reversed SASA gate (paired Wilcoxon $p < 0.001$). For M2 (e), full HEIWA resulted in significantly lower RMSF than the ESM3 baseline (paired Wilcoxon $p < 0.001$) but did not differ significantly from the reversed SASA gate (paired Wilcoxon $p > 0.05$). For both seeds (d,e), the reversed SASA gate resulted in significantly lower RMSF than the ESM3 baseline (paired Wilcoxon $p < 0.001$). The first 5 and last 10 residues were excluded from the paired Wilcoxon test.

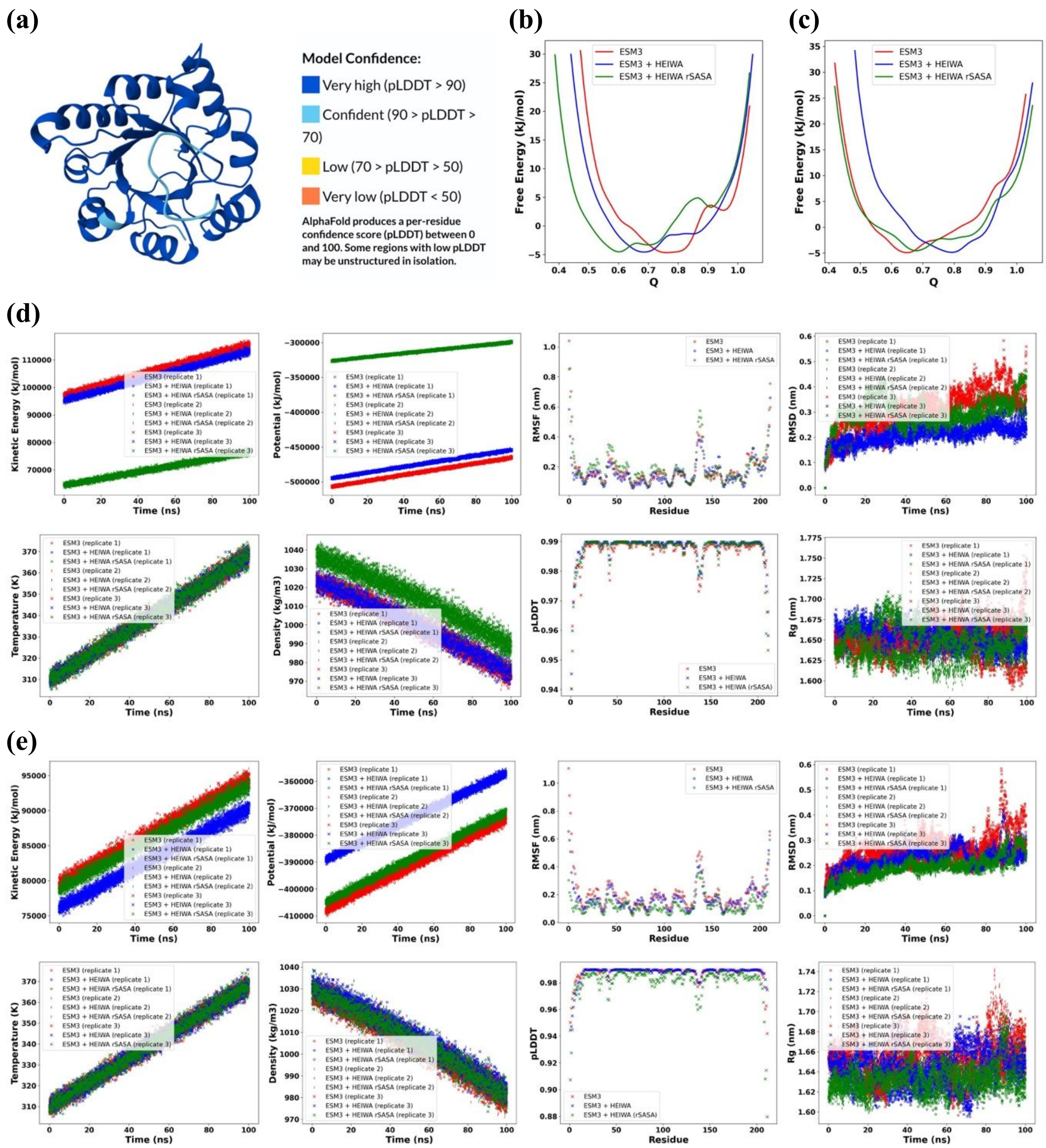

Figure S18. Gromacs molecular dynamics results for Q8X6Y0. For H4 (d), full HEIWA resulted in significantly lower RMSF than the ESM3 baseline (paired Wilcoxon $p < 0.05$) and than with the reversed SASA gate (paired Wilcoxon $p < 0.01$). The reversed SASA gate resulted in significantly higher RMSF than the ESM3 baseline (paired Wilcoxon $p < 0.001$). For M2 (e), full HEIWA resulted in significantly lower RMSF than the ESM3 baseline (paired Wilcoxon $p < 0.001$) but significantly higher than RMSF with the reversed SASA gate (paired Wilcoxon $p < 0.001$). The reversed SASA gate resulted in significantly lower RMSF than the ESM3 baseline (paired Wilcoxon $p < 0.001$). The first 5 and last 5 residues were excluded from the paired Wilcoxon test.

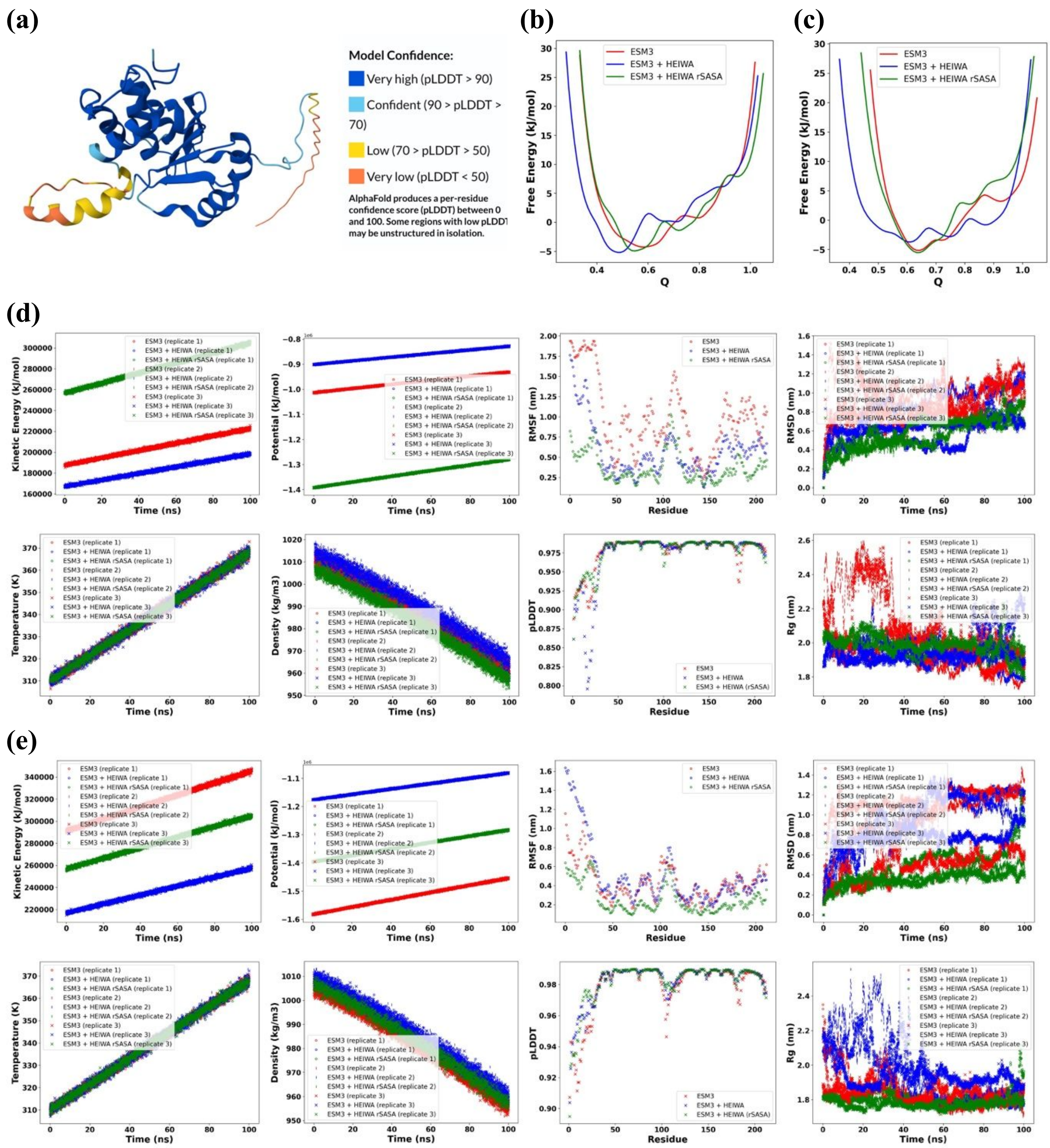

Figure S19. Gromacs molecular dynamics results for A3LW52. For H4 (d), full HEIWA resulted in significantly lower RMSF than the ESM3 baseline (paired Wilcoxon $p < 0.001$) but significantly higher than RMSF with the reversed SASA gate (paired Wilcoxon $p < 0.001$). For M2 (e), RMSF of full HEIWA did not differ significantly from the ESM3 baseline (paired Wilcoxon $p > 0.05$) but was significantly higher than with the reversed SASA gate (paired Wilcoxon $p < 0.001$). For both seeds (d,e), the reversed SASA gate resulted in significantly lower RMSF than the ESM3 baseline (paired Wilcoxon $p < 0.001$). The first 30 residues were excluded from the paired Wilcoxon test.

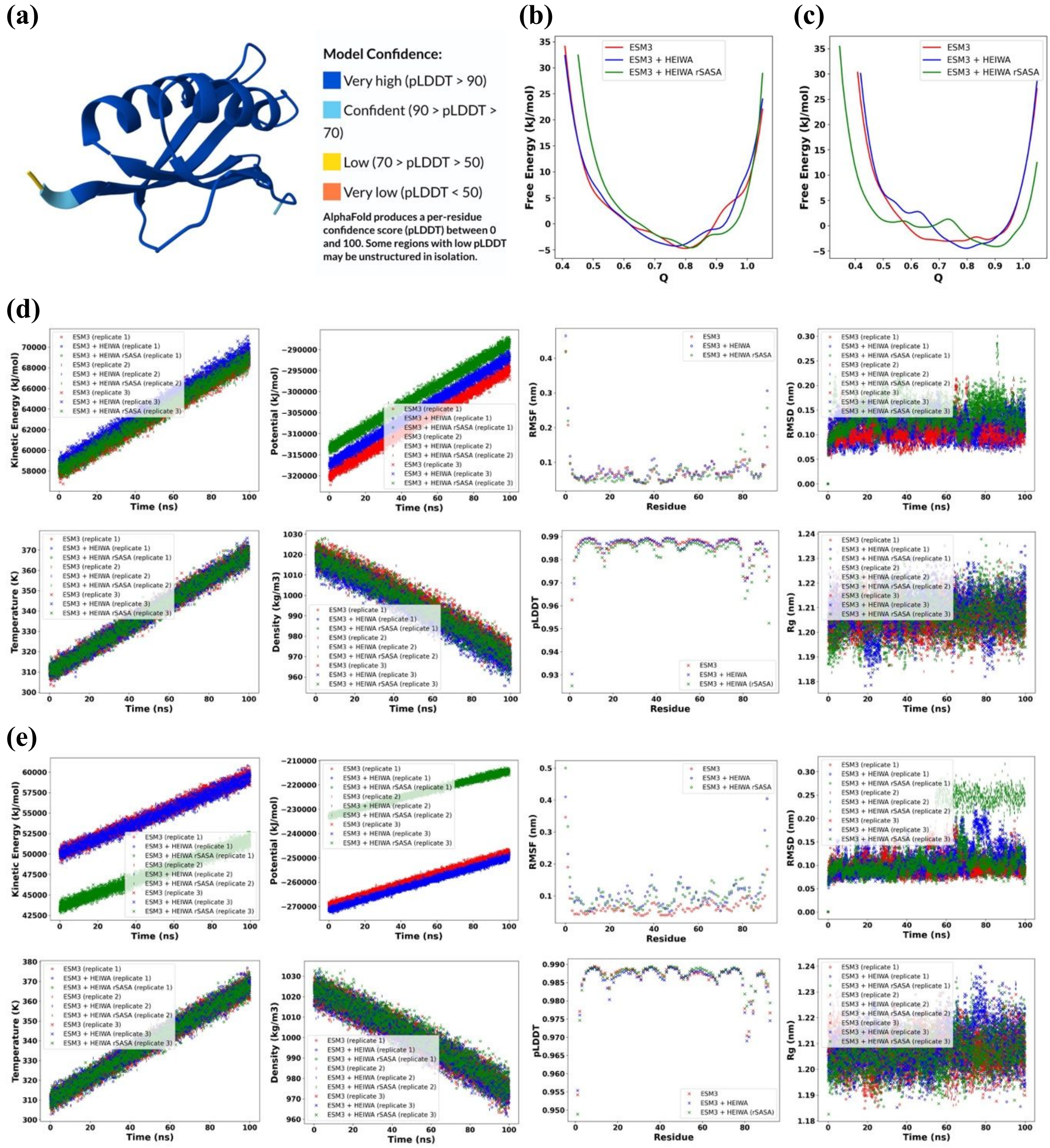

Figure S20. Gromacs molecular dynamics results for Q21YG7. For H4 (d), the RMSF of full HEIWA did not differ significantly from the ESM3 baseline (paired Wilcoxon $p > 0.05$) but was significantly higher than with the reversed SASA gate (paired Wilcoxon $p < 0.001$). The reversed SASA gate resulted in significantly lower RMSF than the ESM3 baseline (paired Wilcoxon $p < 0.001$). For M2 (e), full HEIWA resulted in significantly higher RMSF than the ESM3 baseline (paired Wilcoxon $p < 0.001$) but did not differ significantly from the reversed SASA gate (paired Wilcoxon $p > 0.05$). The reversed SASA gate resulted in significantly higher RMSF than the ESM3 baseline (paired Wilcoxon $p < 0.001$). The first 5 and last 5 residues were excluded from the paired Wilcoxon test.

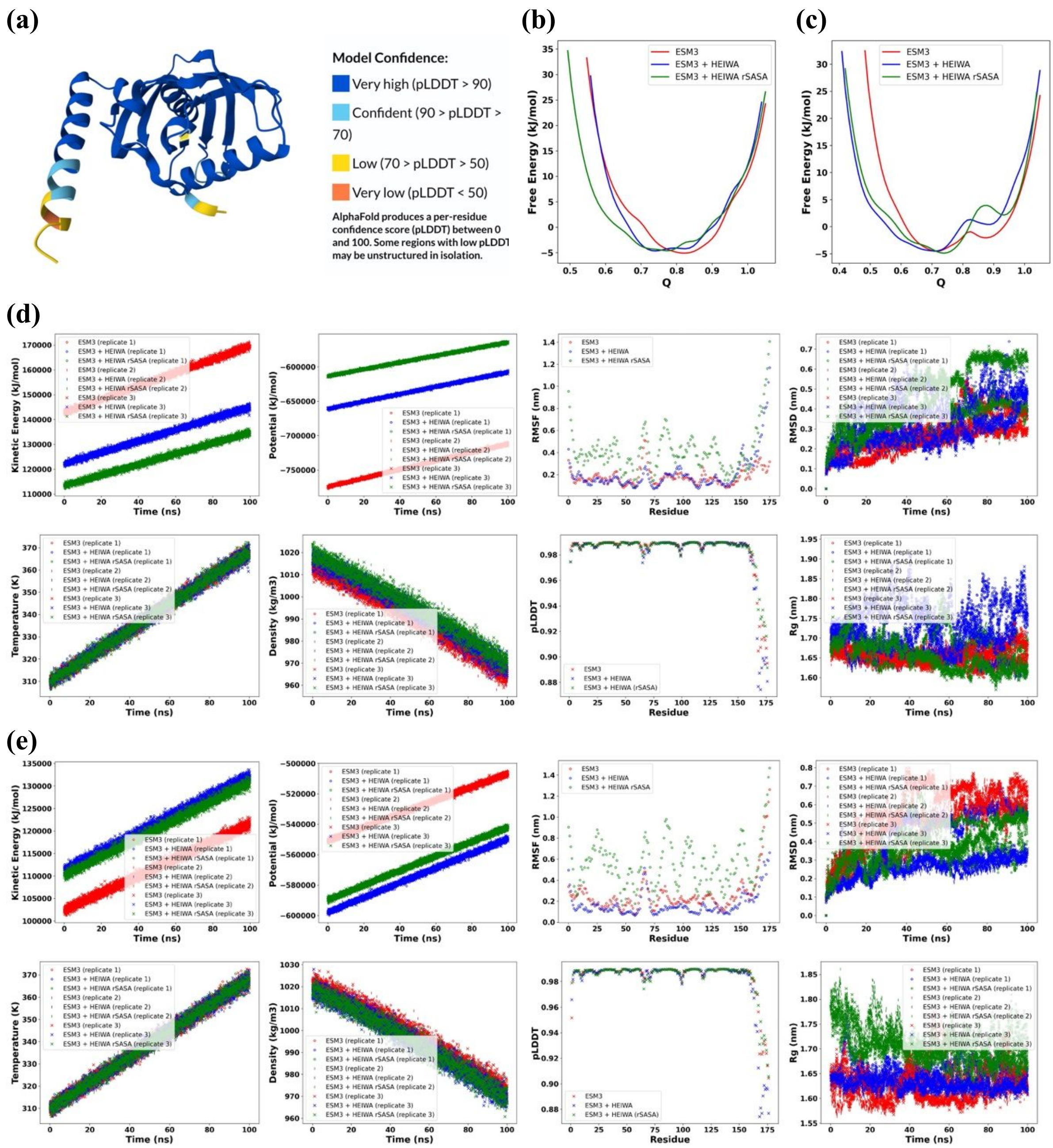

Figure S21. Gromacs molecular dynamics results for Q6G5F0. For H4 (d), the RMSF of full HEIWA did not differ significantly from the ESM3 baseline (paired Wilcoxon p=0.051) but was significantly lower than with the reversed SASA gate (paired Wilcoxon $p < 0.001$). For M2 (e), full HEIWA resulted in significantly lower RMSF than the ESM3 baseline (paired Wilcoxon $p < 0.001$) and than with the reversed SASA gate (paired Wilcoxon $p < 0.001$). For both seeds (d,e), the reversed SASA gate resulted in significantly higher RMSF than the ESM3 baseline (paired Wilcoxon $p < 0.001$). The first 5 and last 25 residues were excluded from the paired Wilcoxon test.

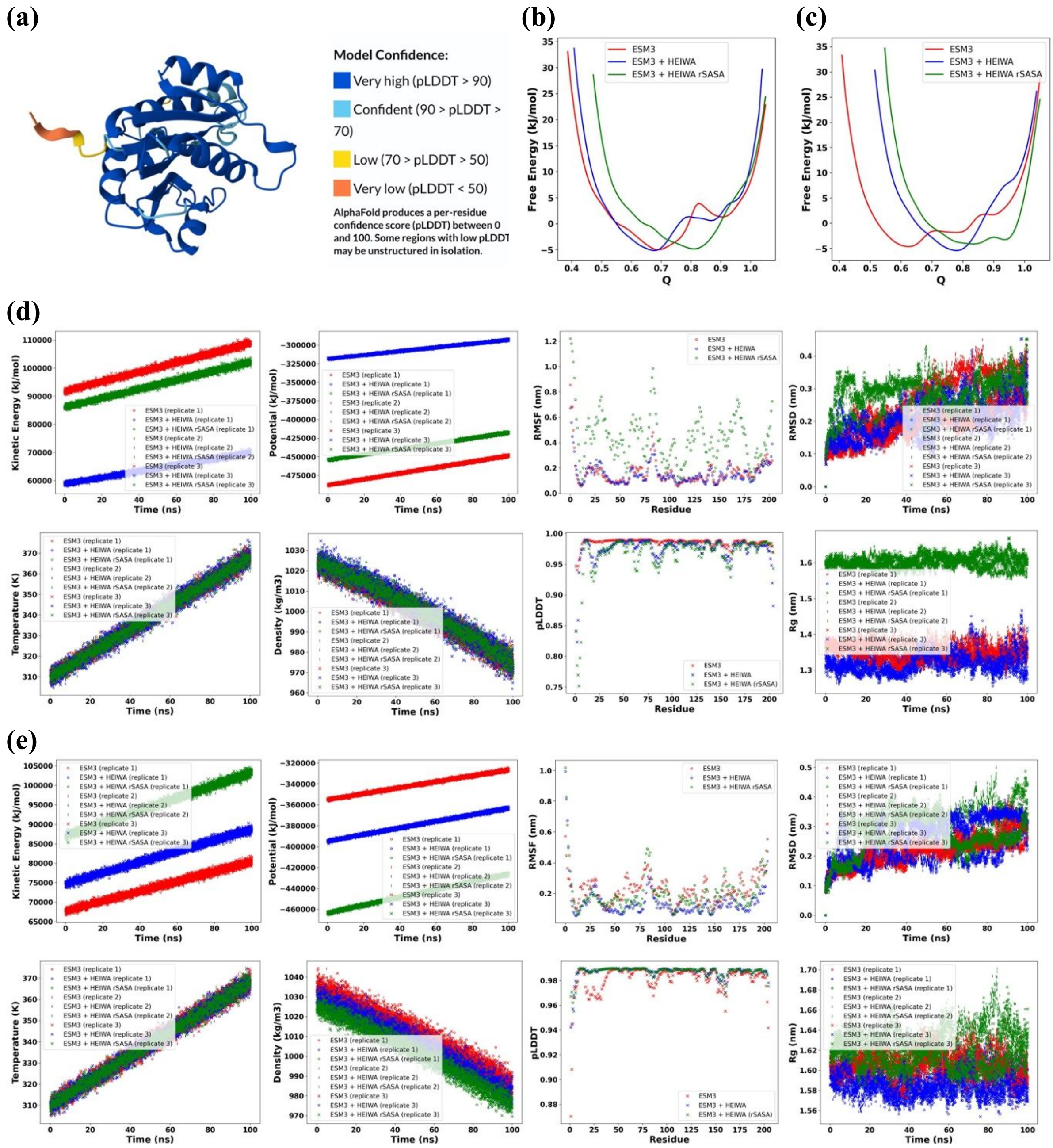

Figure S22. Gromacs molecular dynamics results for B1MDP6. For H4 (d), the RMSF of full HEIWA did not differ significantly from the ESM3 baseline (paired Wilcoxon $p > 0.05$) but was significantly lower than with the reversed SASA gate (paired Wilcoxon $p < 0.001$). The reversed SASA gate resulted in significantly higher RMSF than the ESM3 baseline (paired Wilcoxon $p < 0.001$). For M2 (e), full HEIWA resulted in significantly lower RMSF than the ESM3 baseline (paired Wilcoxon $p < 0.001$) and with the reversed SASA gate (paired Wilcoxon $p < 0.001$). The reversed SASA gate resulted in significantly lower RMSF than the ESM3 baseline (paired Wilcoxon $p < 0.001$). The first 10 and last 5 residues were excluded from the paired Wilcoxon test.

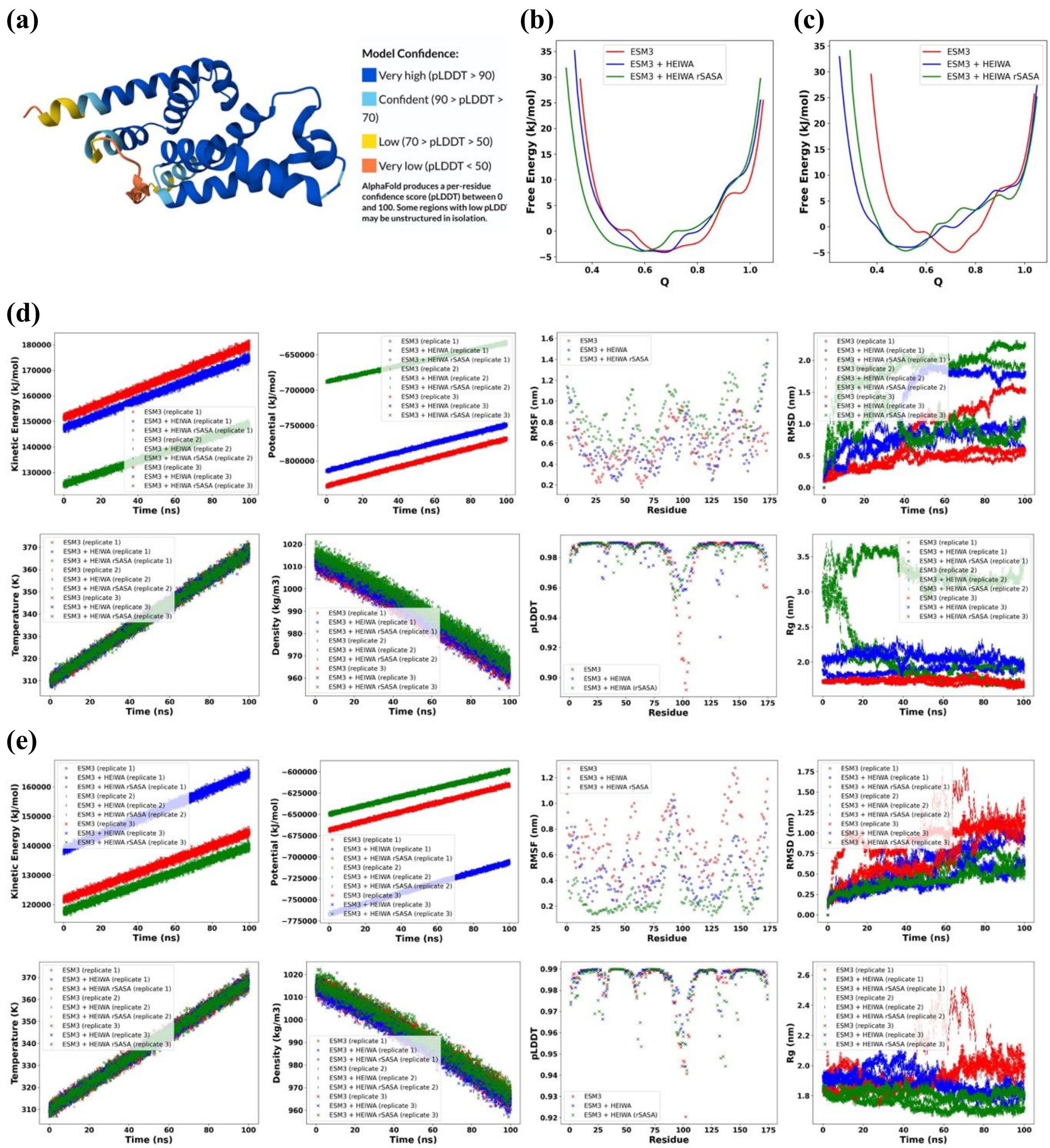

Figure S23. Gromacs molecular dynamics results for Q06909. For H4 (d), the RMSF of full HEIWA did not differ significantly from the ESM3 baseline (paired Wilcoxon $p > 0.05$) but was significantly lower than with the reversed SASA gate (paired Wilcoxon $p < 0.001$). The reversed SASA gate resulted in RMSF significantly higher than the ESM3 baseline (paired Wilcoxon $p < 0.001$). For M2 (e), full HEIWA resulted in significantly lower RMSF than the ESM3 baseline (paired Wilcoxon $p < 0.001$) but significantly higher than RMSF with the reversed SASA gate (paired Wilcoxon $p < 0.001$). The reversed SASA gate resulted in significantly lower RMSF than the ESM3 baseline (paired Wilcoxon $p < 0.001$). The first 10 and last 10 residues were excluded from the paired Wilcoxon test.

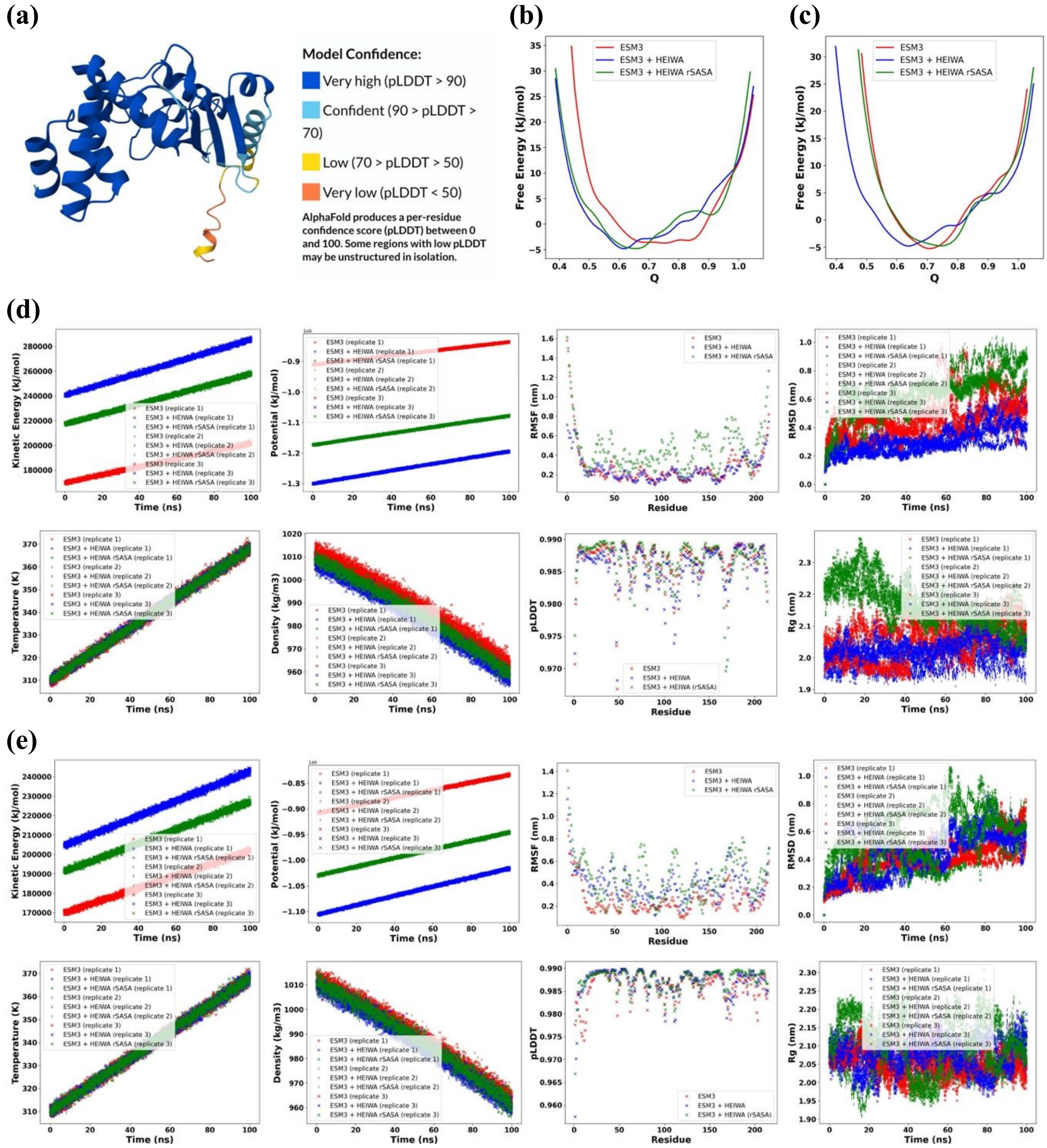

Figure S24. Gromacs molecular dynamics results for P52484. For H4 (d), the RMSF of full HEIWA did not differ significantly from the ESM3 baseline (paired Wilcoxon $p > 0.05$) but was significantly lower than with the reversed SASA gate (paired Wilcoxon $p < 0.001$). For M2 (e), full HEIWA resulted in significantly higher RMSF than the ESM3 baseline (paired Wilcoxon $p < 0.001$) but significantly lower than RMSF with the reversed SASA gate (paired Wilcoxon $p < 0.01$). For both seeds (d,e), the reversed SASA gate resulted in significantly higher RMSF than the ESM3 baseline (paired Wilcoxon $p < 0.001$). The first 15 and last 5 residues were excluded from the paired Wilcoxon test.

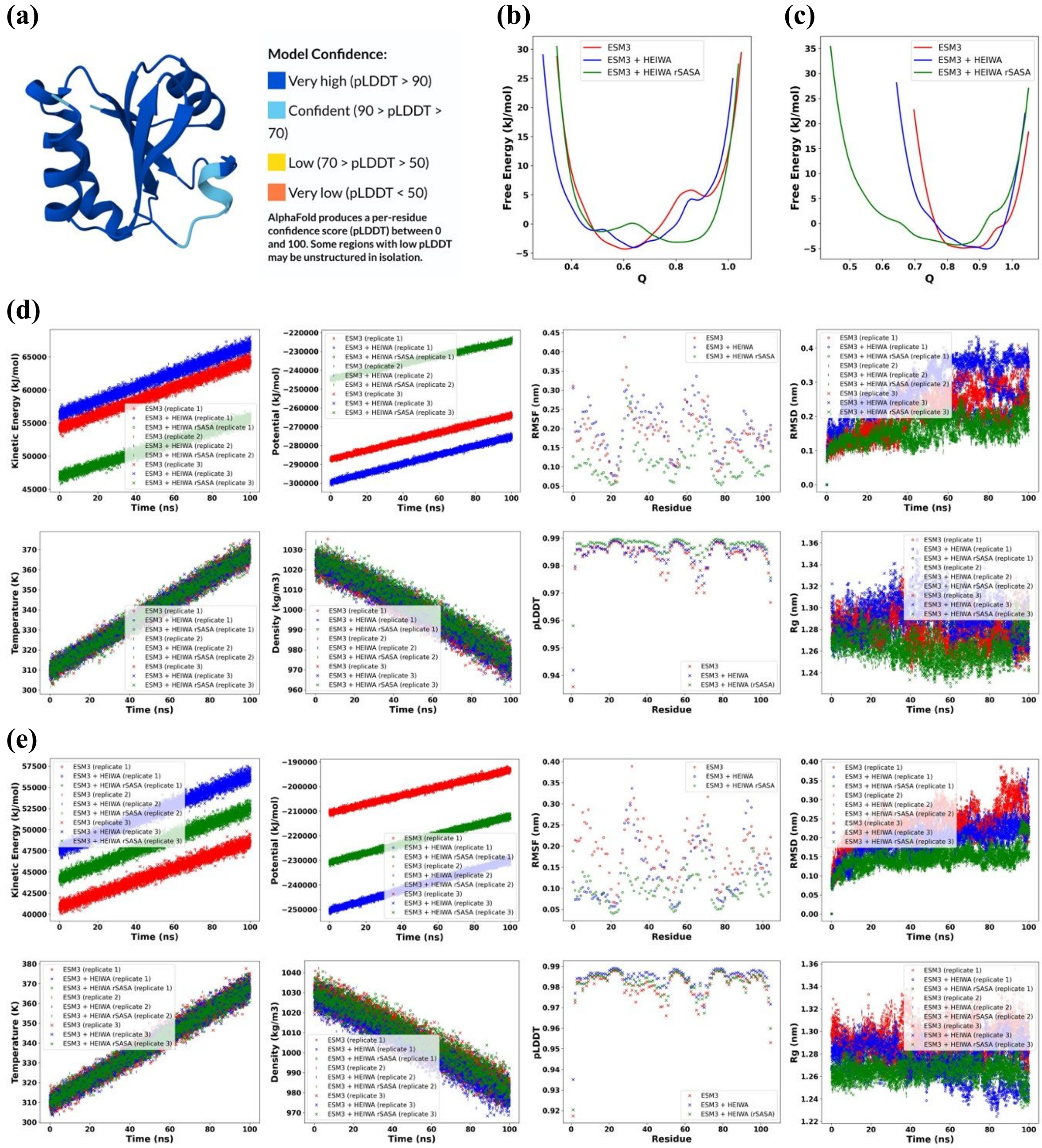

Figure S25. Gromacs molecular dynamics results for Q54KN7. For H4 (d), full HEIWA resulted in significantly higher RMSF than the ESM3 baseline (paired Wilcoxon $p < 0.001$) and than with the reversed SASA gate (paired Wilcoxon $p < 0.001$). For M2 (e), full HEIWA resulted in significantly lower RMSF than the ESM3 baseline (paired Wilcoxon $p < 0.001$) but significantly higher than RMSF with the reversed SASA gate (paired Wilcoxon $p < 0.001$). For both seeds (d,e), the reversed SASA gate resulted in significantly lower RMSF than the ESM3 baseline (paired Wilcoxon $p < 0.001$).

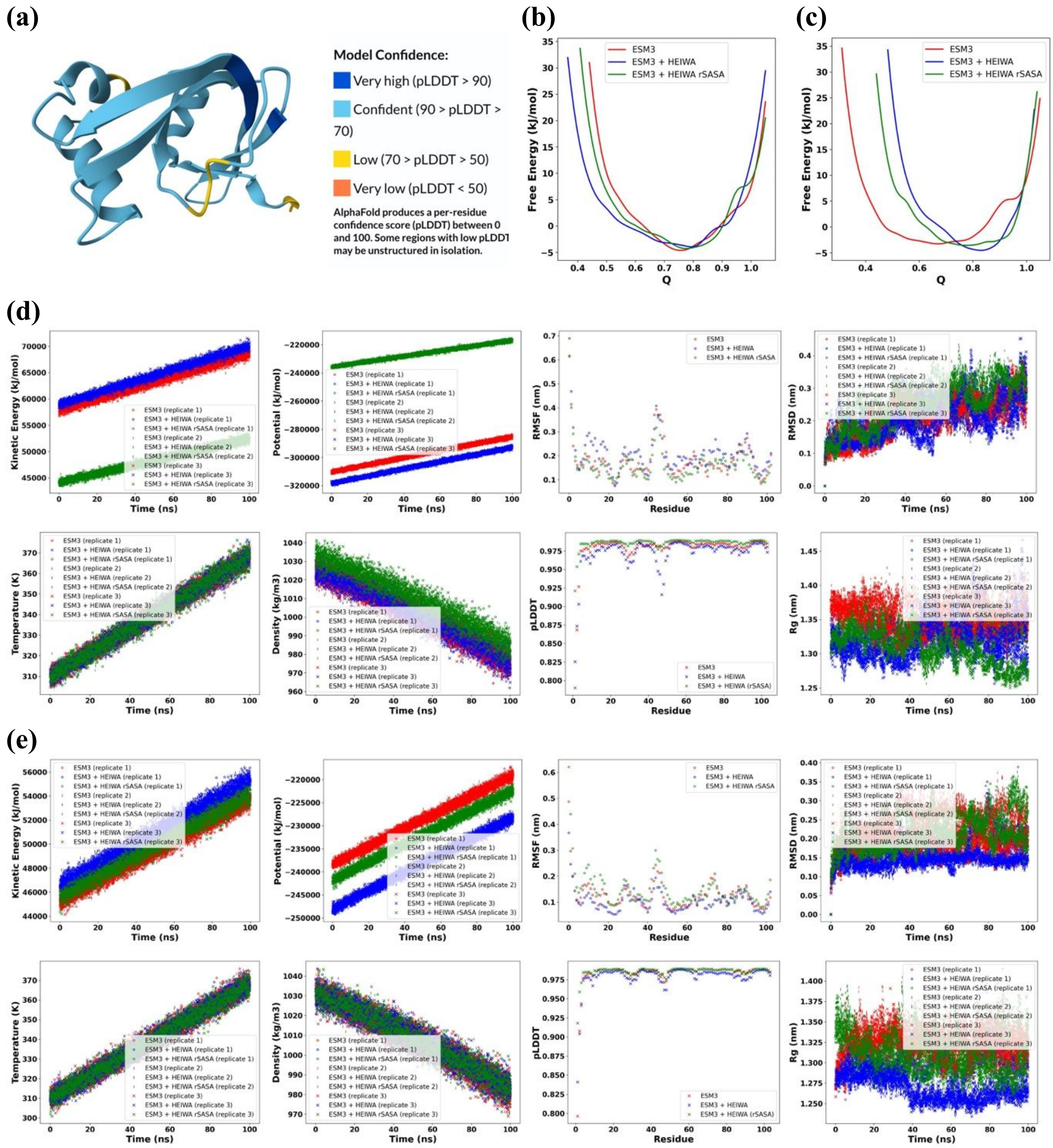

Figure S26. Gromacs molecular dynamics results for B6I733. For H4 (d), full HEIWA resulted in significantly higher RMSF than the ESM3 baseline (paired Wilcoxon $p < 0.001$) and than with the reversed SASA gate (paired Wilcoxon $p < 0.001$). The reversed SASA gate resulted in significantly lower RMSF than the ESM3 baseline (paired Wilcoxon $p < 0.001$). For M2 (e), full HEIWA resulted in significantly lower RMSF than the ESM3 baseline (paired Wilcoxon $p < 0.001$) and with the reversed SASA gate (paired Wilcoxon $p < 0.001$). The reversed SASA gate resulted in significantly higher RMSF than the ESM3 baseline (paired Wilcoxon $p < 0.001$). The first 5 residues were excluded from the paired Wilcoxon test.

Table S1. Description of filtering procedures for UniProtKB reviewed data. All keyword-based filtering are case-insensitive.

Filter target	Method
Exclude non-disulfide bond post-translational modifications	• Exclude all non-NA entries in 'Post-translational modification' unless the containing 'isulfide'.<br>• Exclude all non-NA entries in 'Cross-link', 'Lipidation', 'Glycosylation', 'Modified residue', 'Propeptide'<br>• Entries with non-canonical amino acids excluded.
Exclude entries with expected protein-protein interactions	• Exclude entries if description contains "chaperon", "heat-shock", "heat shock", "complex", "subunit", "inhibitor", "assembly", "multimer", "chain", "component" under the fields "Protein names", "Subunit structure", "Function [CC]", and all gene ontology-related fields.<br>• Entries labelled as contained interchain disulfide bonds are excluded.
Exclude uncertain signal/transit peptide	• All signal/transit peptide with unknown cleavage site with virus origin are excluded.<br>• Discard sequence if the cleavage site cannot be identified by deepTMHMM [1] or TargetP [2].<br>• Initiator methionine is removed if indicated under the field "Initiator methionine".
Ensure consistent sequence between UniProt entry and AlphaFold PDB	Sequences meeting the following criteria are retained:<br>• Identical sequences after signal/transit peptide truncation.<br>• Length of mature sequences differed by less than 15 residues and one sequence exactly starts with or ends with the other sequence.
Classification of protein according to optimal temperature, pressure, pH, and halophilicity	• Thermophilic proteins are identified by the following criteria<br>o In Bacdive database, taxid with culturing temperature over 60°C.<br>o UniProt label under "Temperature dependence" indicated either an optimal temperature over 70°C or melting temperature over 85°C.<br>o Species name contained "pyro", "therm", "geobacillus", "yellowstone".<br>• Cryophilic proteins are identified by the following criteria<br>o In Bacdive database, taxid with culturing temperature below 16°C.<br>o UniProt label under "Temperature dependence" indicated either an optimal temperature below 16°C or melting temperature below 25°C.<br>o Species name contained "cryo", "arctic", "antarctic".<br>• Piezophilic proteins are identified by the following criteria<br>o In Bacdive database, taxid isolated from deep lake, deep sea (>100 m), deep sea sediment, deep soil or groundwater (>20 m), and artesian.

	o Species name contained “piezo”, “abyss”, “artesia”, “aquifer”, “hydrotherm”.<br>• Acidophilic proteins are identified by the following criteria<br>o In Bacdive database, taxid with culturing temperature below pH 4. But species known as human pathogen are excluded.<br>o UniProt label under “pH dependence” indicated an optimal pH below 4.<br>o Species name contained “acid”.<br>• Alkaliphilic proteins are identified by the following criteria<br>o In Bacdive database, taxid with culturing temperature above pH 9.5. But species known as human pathogen are excluded<br>o Species name contained “alkali”.<br>• Halophilic proteins are identified by the following criteria<br>o In Bacdive database, taxid with culturing salinity above 5%.<br>o Species name contained “salin” or “halo”.
Exclude category with inadequate samples	• All retained SUPFAM must include both mesophilic and thermophilic proteins.<br>• Each SUPFAM family should contain up to 20 unique sequences.<br>• For SUPFAM-stratified dataset, only SUPFAM with at least 5 samples are retained.

Table S2. Summary of RMSD after relaxation, including the 30-ns pre-run.

Numbers	No HEIWA (-)		With HEIWA (+)		Reverse Gate (r)	
	H4	M2	H4	M2	H4	M2
G4YEI5	1.70	1.16	1.56	1.55	1.21	1.56
Q7SIG3	1.85	1.53	1.67	1.62	1.65	1.41
P20605	2.85	1.97	2.04	2.45	2.80	3.13
A1C603`	3.37	3.25	4.57	3.02	3.60	3.55
O32858	3.01	3.98	1.74	2.18	2.19	2.38
Q6G4Y4`	2.35	2.27	2.49	2.92	2.19	3.11
Q74K70	2.74	2.18	1.82	1.84	1.47	2.05
Q6NLQ3	2.41	3.15	1.60	2.55	2.00	2.30
Q602T2	2.44	2.90	3.82	2.57	2.41	2.50
Q81ST8	2.52	2.05	1.83	3.14	2.20	2.69
Q8X6Y0	1.58	1.61	1.72	1.39	1.71	1.66
A3LW52`	5.69	5.89	4.04	3.61	5.51	4.39
Q21YG7	0.76	0.70	0.68	0.73	0.86	0.70
Q6G5F0	1.37	1.97	1.59	2.15	2.16	2.21
B1MDP6	1.69	1.80	1.60	1.39	1.61	1.59
Q06909	1.84	2.38	10.51	6.84	18.91	6.65
P52484	2.50	2.93	1.80	1.85	3.72	2.53
Q54KN7	1.65	1.81	2.09	1.69	1.89	1.02
B6I733	1.60	1.63	1.54	1.59	1.69	1.67

RMSD was calculated using backbone atoms in PyMOL, with a 2-Å threshold and 2 iterations. The symbol ` indicates that the protein has disordered loops in addition to the head and tail. H4 and M2 refer to two random seeds. H4 is the same seed as epoch 3 of HEIWA model training, while M2 is an independent seed. For each random seed, the treatment with lowest RMSD is underlined. RMSD values greater than 2 Å are shown in red.

REFERENCES

[1] Hallgren, J.; Tsirigos, K. D.; Pedersen, M. D.; Almagro Armenteros, J. J.; Marcatili, P.; Nielsen, H.; Krogh, A.; Winther, O. DeepTMHMM predicts alpha and beta transmembrane proteins using deep neural networks. bioRxiv, April 8, 2022. DOI: 10.1101/2022.04.08.487609.

[2] Almagro Armenteros, J. J.; Salvatore, M.; Emanuelsson, O.; Winther, O.; von Heijne, G.; Elofsson, A.; Nielsen, H. Detecting sequence signals in targeting peptides using deep learning. *Life Science Alliance* 2019, 2 (5), e201900429. DOI: 10.26508/lsa.201900429.
